# Practical Guide to the Design of Granular Hydrogels for Customizing Complex Cellular Microenvironments

**DOI:** 10.1002/adhm.202501947

**Published:** 2025-07-29

**Authors:** Shuhan Feng, Kaiyang Chen, Shiqi Wang

**Affiliations:** ^1^ Drug Research Program Division of Pharmaceutical Chemistry and Technology Faculty of Pharmacy University of Helsinki Helsinki Finland; ^2^ Institute of Biotechnology Helsinki Institute of Life Science University of Helsinki Finland

**Keywords:** granular hydrogels, assembly, extracellular microenvironment, microgel

## Abstract

Granular hydrogels are a novel class of microporous platforms for cell culture and delivery, formed as macroscopic aggregates through the bottom‐up assembly of microgels. Given their flexibility and diversity, granular scaffolds have attracted extensive attention as emerging materials replicating the complex, heterogeneous environments found in natural tissues. This review outlines the design principles of granular hydrogels, highlighting critical intra‐microgel and inter‐microgel factors that determine the final physicochemical properties of the entire system for creating a biomimetic cellular microenvironment. Intra‐microgel factors represent the intrinsic properties of microgels, while inter‐microgel factors primarily focus on the interactions between microgels. A comprehensive analysis is conducted on each intra‐ and inter‐microgel factor, elaborating on their definitions, classifications, and regulation strategies. Subsequently, the final properties of granular hydrogels, such as porosity, mechanical characteristics, degradability, heterogeneity, drug loading, and cellular incorporation strategy, are discussed in detail with an emphasis on their effects on cellular behavior. Finally, the current technical challenges in granular hydrogel design are discussed along with potential opportunities for further development.

## Graphical abstract

Granular hydrogels are emerging microporous platforms for cell culture and delivery, showing great potential for replicating the complex, heterogeneous environments found in natural tissues. This review outlines the design principles of granular hydrogels, highlighting critical factors that determine the final physicochemical properties of the entire granular system, with a focus on their role in biomimetic cellular microenvironments.

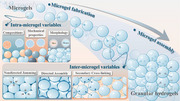

## 1 Introduction

Hydrogel networks, intricately woven from various hydrophilic polymers and possessing excellent water retention capacity, have emerged as competitive candidates for tissue repair and regeneration. They can closely mimic the physicochemical and mechanical properties of the extracellular matrix (ECM) under hydrated state by rationally controlling their chemical composition, polymer concentration, interaction strategy, and cross‐linking density.^[^ ^1^ ^]^ The selection of highly compatible polymer components and biomimetic modifications allows hydrogels to provide not only mechanical support but also adhesion targets for cells, making them highly versatile as tunable biomaterial scaffolds for two‐dimensional (2D) or three‐dimensional (3D) cell cultures. Despite these advantages, conventional bulk hydrogel networks only process molecular‐sized pores (1–10 nm).^[^ ^2^ ^]^ In contrast, natural ECM bundles with variable micrometer‐scale pores, allowing cell migration via a wide array of mechanisms. In other words, bulk hydrogel artificial ECMs impose physical constraints on micrometer‐scale cells. In these cases, cells are considered to be mechanically confined within a nanomesh structure, which is controlled by pore size and matrix degradability of hydrogel networks.^[^ ^3^ ^]^ For example, it has been found that the cross‐sectional area of​ pores as small as 7 µm2 is sufficient to act as a physical barrier to prevent tumor cell migration, as the rigid cell nuclei are unable to squeezing through such small pores.^[^ ^4^ ^]^ In this case, cells rely heavily on matrix metalloproteinase (MMP)‐dependent degradation to overcome matrix confinement and migrate via integrin‐ and actomyosin‐dependent force.^[^ ^3a^ ^]^ Although the pore size and degradability can be tuned by modifying the matrix concentration and cross‐linking density, this may also alter the subsequently transport of oxygen and nutrients as well as the excretion of cellular metabolites. Therefore, cell proliferation and intercellular communication would be hindered within bulk hydrogel microenvironments, while delaying the deposition of secreted ECM.^[^ ^5^ ^]^ To overcome the limitations of the bulk hydrogel nano‐network, the development of hydrogel scaffold systems with micrometer‐scale pores provides an indispensable future direction.

Currently, the manufacturing strategies of microporous hydrogels can be divided into two categories: i) multiscale pore engineering and ii) material‐based bottom‐up tissue engineering. The former involves advanced control of the overall porous microstructure of hydrogels by using solvent porogen leaching, freeze‐drying, gas foaming, and micro‐molding techniques, as detailed in the summary by Annabi et al. and Foudazi et al.^[^ ^2^, ^6^ ^]^ However, the above techniques are restricted to the fabrication of scaffolds in vitro due to their reliance on additives and postprocessing steps. These procedures may lack cytocompatibility and are not always suitable for their intended applications, particularly in vivo situations where injectable implantation or smaller size is required. As an alternative, bottom‐up tissue engineering is the process of fusing micrometer‐sized gel building blocks (also called hydrogel microparticles, HMPs) into larger geometric structures through self‐assembly or physicochemical interactions.^[^ ^7^ ^]^ Hydrogel scaffolds synthesized using a bottom‐up approach are commonly referred to as microporous particle hydrogels or granular hydrogels, which overcome the limitations of traditional bulk gels on the diffusion of nutrients, gases, and soluble factors, meanwhile providing cells with micrometer‐scale interconnected channels to maintain cell morphology, high vitality, and normal differentiation.^[^ ^1b^ ^]^ Compared with microporous hydrogels generated through multiscale pore engineering, the small size and shear‐thinning behavior of microgel particles within granular hydrogels allow them to be injected through small needles and catheters, enabling minimally invasive delivery of cells with perfect conformation to the shape of the target site.^[^ ^8^ ^]^ Furthermore, multiple modular factors within and between microgels (e.g., composition, morphology, mechanical strength, and assembly) influence the microscopic morphology and macroscopic physicochemical properties of final granular hydrogels, supporting researchers with a broad range of options to achieve multiscale regulation of scaffolds, and further mimicking complex microenvironments for cell culture or delivery.^[^ ^3^, ^9^ ^]^ Meanwhile, microgels can be further designed and optimized as functional carriers with a high specific surface area for exogenous cells, cytokines, or small molecules to induce cell behavior or achieve therapeutic purposes in vitro or in vivo.^[^ ^10^ ^]^

The focus of the current review is on the granular hydrogels in a jammed or crosslinked state for applications in targeted delivery, tissue engineering, medical repair since 2000. Special emphasis is placed on the design principles and parameters that determine the granular hydrogel properties as microporous ECM. Accordingly, systems in which microgel particles remain in suspension are not included in the scope of this review. In addition, two‐phase systems, wherein hydrogel microparticles are embedded within another hydrogel medium, were intentionally excluded due to the presence of an additional nanoscale polymer network.^[^ ^11^ ^]^ Although previous reviews have, to some extent, mentioned tunable parameters in the design principles when discussing granular hydrogel applications in bioprinting, bio‐fabrication, and biomedical application, a systematic review and summary of the key factors is still lacking.^[^ ^3^, ^12^ ^]^ Herein, we present updated perspectives and a comprehensive data summary on granular systems as microscale scaffolds for simulating the natural cellular microenvironment, with a primary focus on cell culture and artificial tissues. For a detailed explanation of literature survey and data collection, please refer to Section 5.

**Scheme 1:**
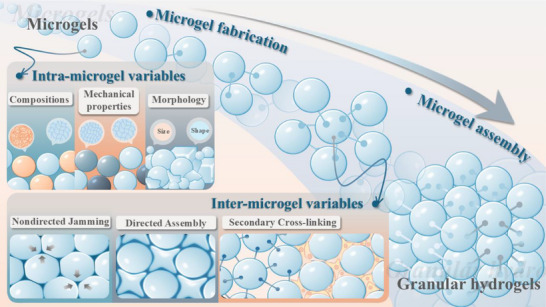
Schematic illustration of granular hydrogel design that comprises two key steps: microgel fabrication and microgel assembly. In the diagram, spheres represent individual microgels. Microgels with distinct polymer compositions are represented by blue and orange spheres, while those with varying mechanical strengths are depicted in gradients of blue—light blue, gray blue, and dark blue. This color scheme is consistently applied across all subsequent figures in this review.

Based on granular manufacturing process, we propose two categories of design considerations (**Scheme** **1**): intra‐ and inter‐microgel factors, with a focus on their definitions, classifications, and regulation strategies, and usage condition. The resulting properties of assembled granular hydrogels, such as porosity, mechanical properties, degradability, heterogeneity, and drug loading, are discussed in detail with an emphasis on their effects on cellular behavior. In the end, we highlight the great potential of granular hydrogels in customizing microenvironments for cell culture and delivery and provide critical insights into future developments of the field.

## 2 Intra‐ and Inter‐microgel Factors in Granular Hydrogel Design

Generally, the development of granular hydrogels can be divided into the two steps of microgel fabrication and assembly (Scheme 1). First, microgels are fabricated via various techniques, including emulsion, microfluidics, extrusion, mechanical fragmentation, and atomization, as reviewed in detail by Farjami and Madadlou.^[^ ^13^ ^]^ Second, different jammed or assembling strategies are introduced to transform the freely stacked microgel blocks into a granular hydrogel. Based on the two‐step granular hydrogel formation process, we classify the variables involved in the fabrication of microgel building blocks as “intra‐microgel” since they are associated with intrinsic properties of individual microgels, including composition, morphology, and mechanical characteristics. On the other hand, the variables related to the interaction and assembly of large populations of microgels described as “inter‐microgel,” including nondirectional packing, directional assembly, and secondary crosslinking strategies among microgel units. By designing and combining the above‐mentioned variable factors, along with the inclusion of different cells and/or bioactive clues, the granular hydrogels with microscale homogeneity or heterogeneity, controllable local mechanical properties, and tunable microporosity can be developed. This outlines the key intra‐microgel and inter‐microgel variables involved in the design and fabrication of granular hydrogels.

### 2.1 Intra‐microgel Factors

#### 2.1.1 Composition of Individual Microgel

**Figure 1:**
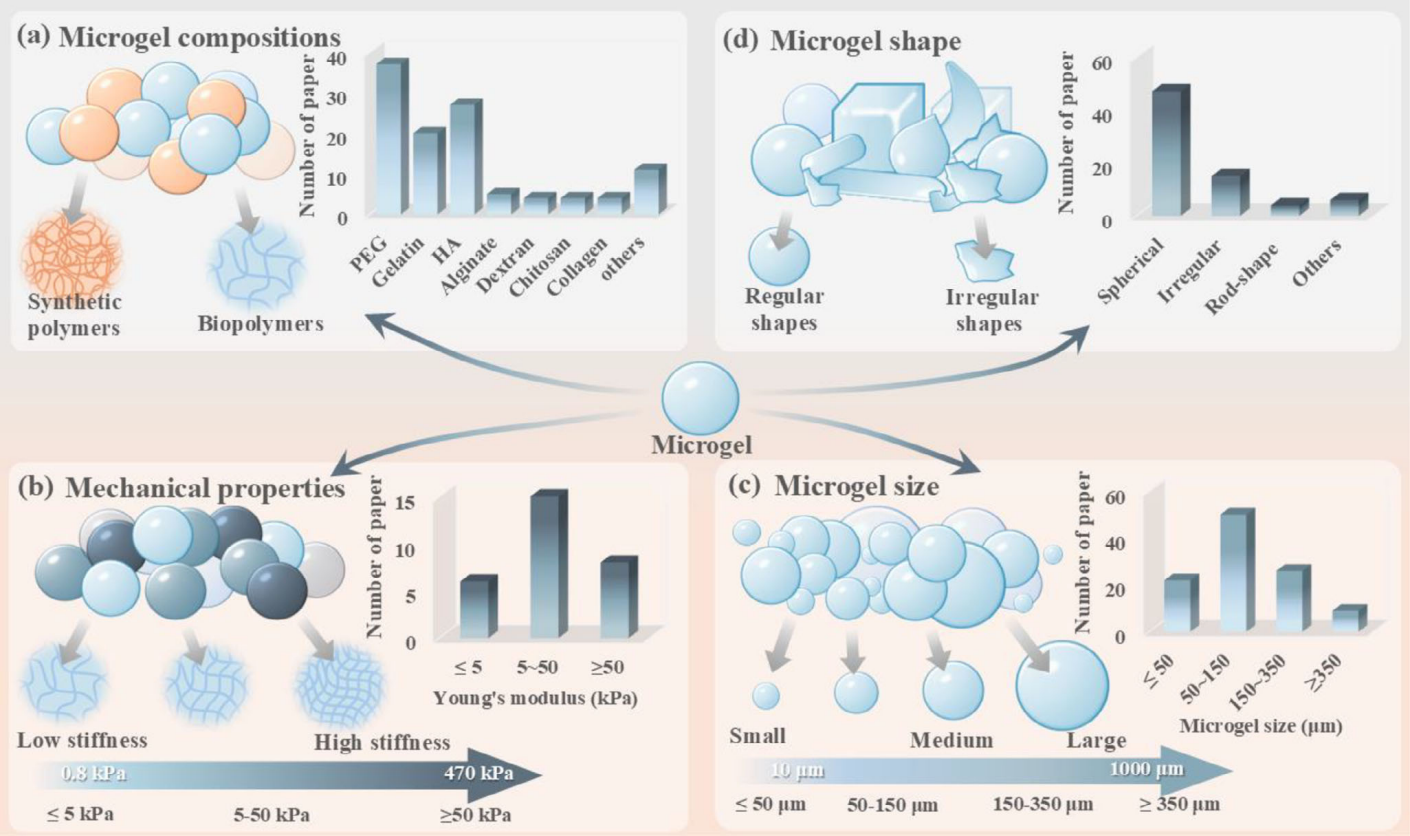
Key intra‐microgel factors of granular hydrogel design. Summaries of a) reported compositions of individual microgels, b) Young's modulus of individual microgels, c) size, and d) shape of individual microgels. The details of each paper included in the literature survey are available in Tables S1 and S2 (Supporting Information).

Currently, microgels used to assemble granular hydrogels are typically fabricated from biomaterials ranging from synthetic polymer (e.g., poly(ethylene glycol) (PEG) and poly(N‐vinylcaprolactam)) to naturally derived biopolymers (e.g., chitosan, gelatin, hyaluronic acid (HA), and alginate), as shown in **Figure** **1**a and Table S1 (Supporting Information).^[^ ^14^ ^]^ Based on their interactions with cells, these materials can be classified as bio‐inert or bio‐responsive. Widely used synthetic matrix scaffolds can be solidified through free radical polymerization of monomers, such as acrylates or methacrylates (Mw < 3 kDa), and multi‐arm covalent interaction of PEG oligomers (Mw 3–20 kDa), as well as physical or chemical cross‐linking of polymers (Mw > 20 kDa). These fully bioinert scaffolds help prevent unwanted immune response and biological side effects. In addition, functionalizing synthetic polymers with cell adhesion peptides (e.g., RGD sequences) allows them exhibit cell‐interaction properties similar to those of natural biopolymers.^[^ ^14^, ^15^ ^]^ The spatial distribution and patterning of coupled adhesion peptides further control cell integration receptor clustering and aggregation. Compared with synthetic materials, biopolymers originated from natural ECM, such as gelatin and HA, can inherently facilitate cell signaling and provide adhesion sites, thereby promoting cell proliferation and differentiation while being naturally metabolized and cleared by the body.^[^ ^16^ ^]^ However, it is worth noting that biopolymers may have limited mechanical tunability and long‐term structural stability, implying that they are more suitable for preparing microgels for rapid tissue repair. In contrast, synthetic materials, with precisely designable molecular weight (Mw), branching degree, and chemical groups, can be functionalized with various reactive groups to achieve precise regulation of mechanical properties and degradation rates of intra‐microgel within granular hydrogels. Depending on the intended application of the final system, the above two types of materials could be carefully selected or combined to allow mechanical customization, predictable degradation, and control over cell responses mimicking the natural tissue environment. Based on our summary of over 100 studies listed in Table S1 (Supporting Information), most current studies prefer using PEG, gelatin, and HA as the matrix materials for granular hydrogel scaffolds (Figure 1a) due to their availability, biocompatibility, and functionalization capability for further secondary cross‐linking between microgels.^[^ ^17^ ^]^

#### 2.1.2 Mechanical Properties of Individual Microgel

The mechanical properties of individual microgels not only determine the matrix rigidity sensed by cells but also influence the overall mechanical flexibility, deformation behavior, and degradability of assembled granular hydrogels. In this section, we focus on the design and how to control the mechanical properties of microgels in the fabrication. For a detailed discussion on how the mechanical properties of individual microgels influence the overall mechanics of granular hydrogels, please refer to Section 3.2.

The mechanical property regulation of individual microgels is theoretically similar to that of conventional bulk hydrogels, as they are originally derived from pulverized or micrometer‐scale bulk hydrogel precursors. This explains why most studies evaluate the mechanical properties of microgels by directly measuring the stiffness of the corresponding bulk hydrogels fabricated from identical precursor formulations.^[^ ^18^ ^]^ A few studies also utilized atomic force microscopy (AFM) to obtain direct mechanical data of individual microgels.^[^ ^16^ ^]^ When examining a single microgel as the unit of analysis, its primary mechanical properties include the elastic modulus (*E*, also known as Young's modulus) and viscoelastic characteristic.^[^ ^19^ ^]^ The elastic modulus is typically quantifies the stiffness of hydrogel, reflecting its resistance to an applied stress. Viscoelasticity describes the combined properties of elastic solid and viscous liquid, exhibiting in phenomena such as stress relaxation, creep, and hysteresis.^[^ ^20^ ^]^ These two mechanical dimensions simultaneously influence the interaction between the individual microgel surface and integrin receptors of cells, which in turn modulates the actin cytoskeleton and altering cell behavior. Among them, the stiffness of microgel provides a greater resistive force to promote cells to exert greater traction force, allowing the actin–myosin system inside the cell to produce stronger stress fibers, thereby enhancing the stable growth of adhesion spots and leading to increased cell spreading.^[^ ^21^ ^]^ Meanwhile, once the relaxation time of microgel is shorter than the dissociation time of adhesion sites, its viscoelasticity enables the microgel surface to provide transient but sufficient mechanical support. This mechanism allows cells to spread on soft and viscoelastic surfaces and to form strong focal adhesions and stress fibers.^[^ ^3a^ ^]^

The mechanical properties of individual microgels are determined by their cross‐linking strategy, cross‐linking density, and post‐crosslinking swelling behavior. Basically, microgel building blocks with viscoelastic properties are produced through reversible cross‐linking strategies, whereas purely elastic microgels generated via irreversible cross‐linking.^[^ ^22^ ^]^ As shown in Table S1 (Supporting Information), common intra‐microgel covalent cross‐linking strategies include rapid click reactions (e.g., for end‐group functionalized PEG, and HA), and free radical chemical polymerization (e.g., for methacrylate, GelMA, and polyacrylamide).^[^ ^16^, ^18^, ^23^ ^]^ These approaches typically produce microgel units with predominantly elastic properties. Only a few studies have employed noncovalent cross‐linking strategies to create viscoelastic microgel units, such as ionic cross‐linking, physical self‐assembly, and guest–host reaction, which are frequently used with alginate, collagen, and gelatin.^[^ ^14^, ^24^ ^]^ This indicates existing gaps in the field and highlights potential opportunities for further research in developing viscoelastic microgels building blocks.

Apart from cross‐linking strategy, regulating cross‐linking density is a more commonly employed approach to control the mechanical strength, specifically the *E*, of individual microgels. Popular approaches include varying the concentration, Mw, degree of functionalization, and crosslinker mixing ratio of the hydrogel precursors. Among them, adjusting concentration of precursors is the easiest way to control cross‐linking density. For instance, Griffin et al. had successfully prepared a range of easily achievable microgel units with *E* from ≈10 to 1000 Pa by varying the PEG weight percentage.^[^ ^14b^ ^]^ In addition, increasing the Mw of polymer components enhances *E* and slows stress relaxation, whereas reducing Mw significantly lowers *E* and accelerates stress relaxation.^[^ ^25^ ^]^ However, the effect of Mw on *E* can be counteracted by the degree of functionalization of hydrogel precursors. For example, by increasing the degree of thiolation in low Mw HA (41–65 kDa), microgel can achieve a comparable *E* value to that formed by high Mw HA (750–10 000 kDa).^[^ ^26^ ^]^ Beyond modifying Mw and functional groups, tunable stiffness of microgel building blocks can also be achieved by modifying the molar ratio of the crosslinker. For example, Tanner et al. obtained irregular microgel units with varying stiffness (*E* = 12–80 kPa) by fragmenting bulk hydrogels formed at different molar ratios of thiol groups on dithiothreitol to norbornene groups on HA‐norbornene (Norb).^[^ ^27^ ^]^ Notably, the mechanical properties of individual hydrogels must also account for their subsequent swelling behavior in the surrounding environment. For example, as observed by Recalde Phillips et al., microgel building blocks composed of low Mw PEG (5 kDa) exhibited higher *E* after swelling than those formed from high Mw PEG (20 kDa).^[^ ^28^ ^]^ Consequently, most recent studies now employ swelling‐equilibrated individual microgel units as modular elements for hydrogel assembly.

By summarizing the relevant data, the common *E* values in the preparation of individual microgels fall in the range of 0.8–470 kPa (Figure 1b). Importantly, to date, no studies specifically targeting the modulation of individual microgel viscoelasticity within granular systems have been published. Thus, the data range of viscoelastic variables in such granular systems and their impact on cellular behavior remain unknown.

#### 2.1.3 Morphologies of Individual Microgel

The morphology of microgels involves two dimensions, i.e., shape and size. Based on the analysis of over 50 studies, microgel diameters predominantly range between 15 and 1000 µm, with various shapes, primarily spherical (Figure 1c,d). These two variables collectively regulate the pore characteristics and injection extrusion behavior of the final granular hydrogels. Specifically, pore characteristics are mainly defined by the void spaces formed between stacked microgel building blocks, which are proportional to the diameter of individual microspheres.^[^ ^10^ ^]^ This result has been demonstrated by progressively larger void spaces between adjacent particles when comparing the spherical microgels with small (40 µm), medium (70 µm), and large (130 µm) diameters.^[^ ^29^ ^]^ Hereafter, formed narrow or spacious intervoid spaces will subsequently affect cell proliferation, migration, and differentiation, which will be discussed in detail in Section 3.1. In addition, the size of the microspheres also plays a crucial role in determining the injectability of jammed granular hydrogels and their extrusion properties as bio‐inks. Sufficiently small microgels can prevent syringe clogging, ensure smooth and continuous filament extrusion, and enhance printing fidelity during injection‐based processing, whether in vivo or via 3D printing in vitro. Conversely, larger microgels are more likely to cause needle clogging and produce rougher filaments, reducing the smoothness and precision of the printed structure.^[^ ^12b^ ^]^ Meanwhile, the greater shear stress required for extrusion of large microgels may affect the activity of cells within the matrix.

Achieving precise regulation of pore properties and extrusion behavior is challenging when only relying on changing the size of low‐anisotropy spherical microgels. Therefore, in recent years, building blocks of different shapes have been increasingly explored to develop a high ratio of their longest dimension to their shortest dimension. This ratio, also known as aspect ratio, indicates the shape anisotropy of microgels.^[^ ^14c^ ^]^ Granular hydrogels have been rapidly assembled (2–3 s) via amine‐epoxy addition reaction by anisometric microgels with high aspect ratios ranging from 2.2 to 4.5. These granular hydrogels have exhibited pore sizes up to 4.5 times larger (25–225 µm) than those formed from spherical microgels (aspect ratio = 1), exhibiting efficient human umbilical vein endothelial cells (HUVECs) growth and spreading, and proliferation of human fibroblasts.^[^ ^30^ ^]^ Furthermore, granular hydrogel scaffolds with broader pore size range (39–82 µm) and higher porosity (65%–90%) were produced by increasing the high aspect ratio of anisometric microgels to 20, ensuring rapid and deep cell invasion.^[^ ^31^ ^]^ Notably, when the aspect ratio is ≥6, the microgel may deform into curved microparticles while maintaining an intact morphology, depending on its mechanical strength.^[^ ^32^ ^]^ Aside from altering high aspect ratios of building blocks, Tang et al. selectively introduced gelatin or dextran as sacrificial materials within PEG microspheres to create crescent‐shaped microgels for enhancing interstitial void space, resulting in an increase in the number of myofibroblasts and leukocytes.^[^ ^33^ ^]^ Based on this, Based on this, a recent study utilized heat‐induced phase separation to form independent phases of GelMA and PEG during cooling for creating microgels with microporous structure after photo‐crosslinking and PEG removing.^[^ ^34^ ^]^

It is worth noting that beyond precise and regular shape (e.g., spherical and rod‐shaped) design via microfluidics or photolithography approach, mechanical fragmentation methods have also been widely employed to produce irregular building blocks (Figure 1). Compare with regular microgels, these irregular microgels exhibit distinct geometric properties, characterized by uneven surface curvature and variable geometries.^[^ ^35^ ^]^ Previous studies have demonstrated that MSCs exhibit distinct differentiation behaviors on adhesive islands with varying geometries while maintaining a constant surface area.^[^ ^36^ ^]^ Meanwhile, the curvature of the surface where cells reside influences cellular mechanical tension, triggering the cytoskeleton to generate counterforces through the formation of high‐tension actin filaments. Therefore, shapes with high aspect ratios and high curvature enhance cell contractility, promoting osteogenic differentiation, while low‐tension shapes favor adipogenic differentiation.^[^ ^36^ ^]^ Furthermore, even with the same curvature, cell attachment differs between convex and concave surfaces. Werner et al. demonstrated that the average migration speed of human mesenchymal stem cells (hMSCs) is higher on concave surfaces than on convex surfaces.^[^ ^37^ ^]^ Supporting this, Assoian et al. explained in their review on cell sensing of micrometer‐scale curvature that on concave surfaces the apical stress fibers of the cell form a straight line spanning the surface, reducing the cell–substrate contact area.^[^ ^38^ ^]^ Meanwhile, the basal stress fibers tend to align with the surface curvature and orient along the direction of minimum curvature, leading to increased cell mobility. In contrast, cells struggle to aggregate in areas of high positive curvature, as they have difficulty forming stable stress fibers and strong adhesion points.^[^ ^38^ ^]^ In practice, granular hydrogels assembled from irregular microgels present a more complex geometric topography, yet this aspect remains largely unexplored. Recently, a study attempted to construct a 2D silica bead array to examine cell behavior on similar structures and found that the distance of nonadhesive areas between microgels regulates cell adhesion and morphology.^[^ ^39^ ^]^ However, in the 3D case, the interplay of microgel shape, deformability, void space, and annealing strategies must be considered simultaneously.

### 2.2 Inter‐microgel Factors

After defining the intra‐microgel factors described above, the microgels must be further concentrated and assembled into granular hydrogel scaffolds. This process involves the jamming behavior of microgels in the packing state, which is closely linked to the initial physicochemical properties of the resulting granular hydrogel. To achieve this state, two aggregation methods can be employed: nondirected packing and directed assembly. Subsequently, the application of secondary cross‐linking to stabilize the assembly will ultimately determine the final properties of the granular hydrogel. This section will analyze these inter‐microgel factors in detail.

#### 2.2.1 Jamming Behavior between Microgels

Generally, prepared microgels tend to readily settle and stack randomly during packing. Under appropriate stress and temperature conditions, once the packing density (also defined as particle volume fraction, φ) of the microgels exceeds 0.58, the entire system will reach random “loose packing state” and undergoes a jamming transition. In this situation, the system gradually transforms from a “liquid‐like” disordered state to an ordered “solid‐like” state, where the internal microgels can move only if their neighboring particles also move.^[^ ^3^, ^40^ ^]^ By reducing the continuous phase volume or applying external mechanical forces (e.g., centripetal force or compression), microgels continue to pack toward a more jammed state. Once φ ≈ 0.64, the maximally jamming state (known as “random close packing”) is achieved, necessitating deformation of the microgel.^[^ ^40^ ^]^ At this stage, the particles are densely packed and fixed by physical interactions with the surrounding particles until sufficient stress is applied to overcome the packing force‐resisting motion, triggering interparticle sliding and shear response behavior. When the applied stress drops below the yield stress, the microgel particles revert to their jammed state, allowing the system to recover its solid‐like behavior with a fixed shape.

The microgel jamming governs the injectability of granular hydrogel systems and is commonly characterized by rheological tests. The viscosity of jammed granular hydrogel decreases as the shear rate increases during shear rate sweep, which is known as “shear‐thinning” or “self‐healing.” The unique shear‐thinning property allows 3D printing and in vivo injection of jammed microgels without additional processing or rearrangement of the molecular structure of the matrix material, thereby ensuring cell viability to a certain degree.^[^ ^41^ ^]^ Under syringe pressure, because the microgels are locked together, they cannot generate turbulence like normal liquids. Instead, they behave like a “plug” or “plunger,” moving forward as a cohesive unit, resulting in more controlled movement.^[^ ^3b^ ^]^ After injection, the structural stability of the granular system can be further enhanced through secondary cross‐linking between microgels, minimizing particle shedding. However, recent studies increasingly utilize dynamically crosslinked microspheres (e.g., host–guest interactions, electrostatic interactions) to eliminate the need for additional fixation at later stages.^[^ ^23^, ^42^ ^]^

Given the various shapes, sizes, and deformability of microgels, the volume fraction of actual jammed granular hydrogels is commonly between 0.58 and 0.64, or even greater than 0.64 for tighter packing. Specifically, the jammed microgels with nonspherical shapes, heterogeneous sizes, and large deformability can result in stronger interparticle friction, thus increasing φ to 0.74.^[^ ^43^ ^]^ For instance, the pore space of granular hydrogels formed by randomly packing polygonal particles created through bulk hydrogel fragmentation was only ≈8%, indicating φ greater than 0.9.^[^ ^44^ ^]^ Compared with scaffolds made of spherical particles, the pores of the granular hydrogels created by irregular shapes microgels with larger contact areas are much smaller.^[^ ^45^ ^]^ Under extreme conditions, when microgels are perfectly arranged as “stacked brick” particles, there is almost no pore volume, implying that the granular hydrogel approaches the characteristics of bulk hydrogels with φ of 1.^[^ ^46^ ^]^

It is worth noting that the inverse relationship between the φ and the void space (i.e., porosity) in granular hydrogels significantly influences both extrudability (printability) and cellular function. For instance, compared to granular hydrogels composed of spherical microgels, granular systems composed of irregularly shaped microgels with higher φ values require substantially greater extrusion forces, potentially compromising cell viability.^[^ ^46^ ^]^ Furthermore, simulations performed in software Blender to predict the jamming paths of spherical and rod‐shaped particles revealed that spherical particles were found to experience more frequent and severe blockages, whereas high‐aspect ratio particles encounter fewer obstructions and exhibit improved pore connectivity and structural anisotropy.^[^ ^47^ ^]^ Therefore, in practical applications, intra‐microgel properties must be carefully selected to ensure that the final scaffold achieves a balanced particle‐to‐pore ratio conducive to cell growth. In addition, when employing granular hydrogels as bioinks, it is crucial to maintain an appropriate φ while preserving high spatial resolution.^[^ ^12a^ ^]^ This requires fine‐tuning of extrusion characteristics, including microgel morphology and nozzle dimensions. For comprehensive discussions on the extrusion behavior, influencing factors, and biological functions of granular hydrogels as emerging bioinks, readers are referred to prior reviews.^[^ ^12^, ^48^ ^]^

#### 2.2.2 Nondirected Packing between Microgels

**Figure 2:**
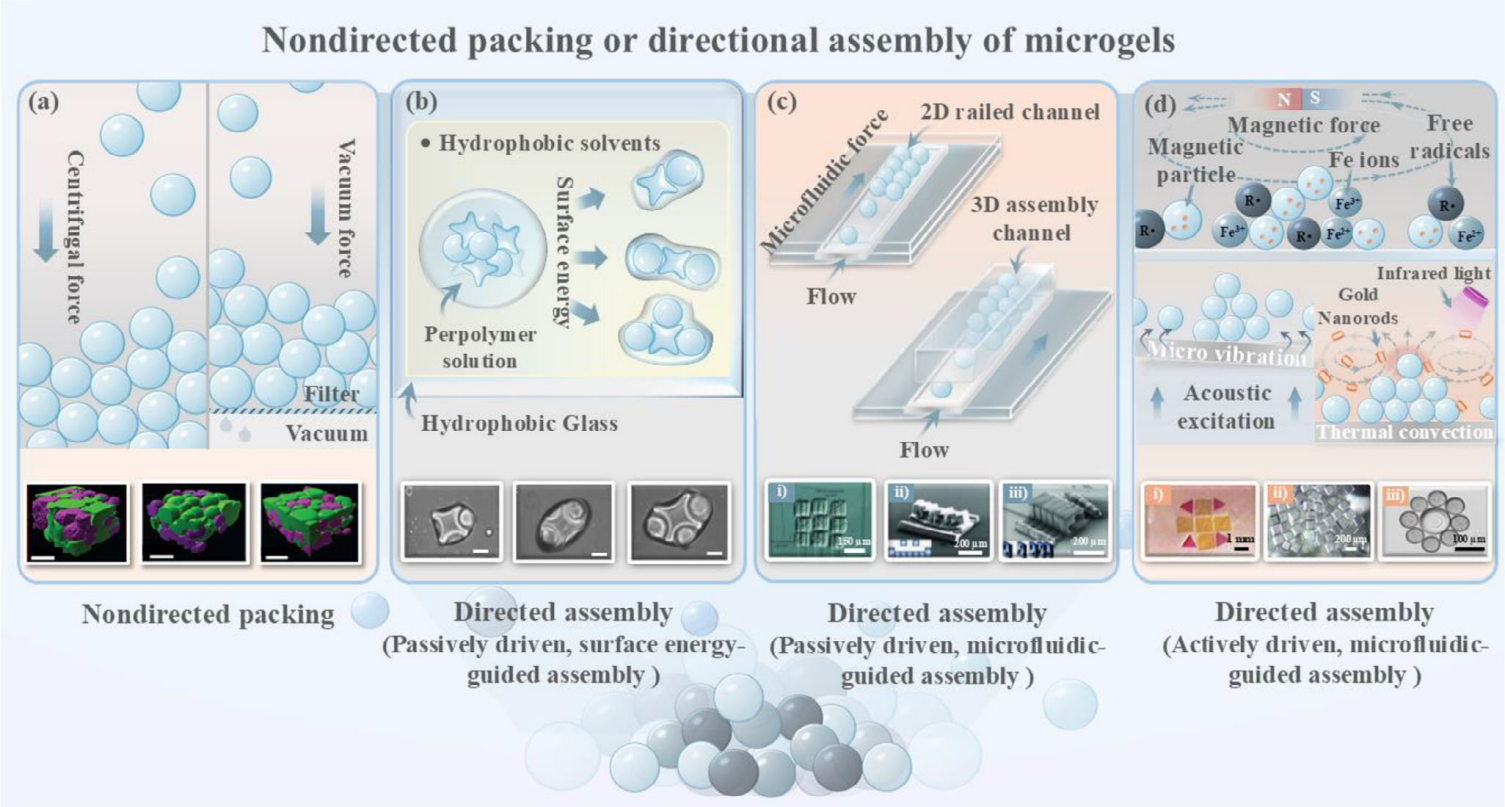
Summary of nondirectional packing and directional assembly strategies for microgels. a) Schematic illustration of nondirected packing driven by centrifugal force and vacuum force, respectively. The inset displays the confocal Z‐stack reconstruction of non‐directionally assembled granular hydrogel formed with MSC spheroids and NorHA microgels. Reproduced with permission.^[^ ^53^ ^]^ Copyright 2024, Wiley. Scale bar is 200 µm. b) Schematic of surface energy‐guided directional assembly. The inset displays lock‐and‐key assemblies incorporating one to three rods per cross driven by surface energy. Reproduced with permission.^[^ ^54^ ^]^ Copyright 2008, National Academy of Sciences. Scale bar is 200 µm. c) Schematic of microfluidic‐guided directional assembly. i) Inset shows a 3 × 3 microscale granular hydrogel incorporating two types of living cells, HeLa and HEK293. Reproduced with permission.^[^ ^55^ ^]^ Copyright 2008, Nature Portfolio. ii) Side‐stamped “SMILE” pattern demonstrating directed self‐assembly of 3D fluids. iii) Scanning electron microscopy (SEM) image of lattice assembly structure. Reproduced with permission.^[^ ^56^ ^]^ Copyright 2011, Wiley. d) Schematic of active assembly strategies utilizing magnetic fields, acoustic waves, and light. i) Inset shows magnetic force reconfiguration of gelatin methacrylate hydrogels with two shapes into complex planar constructs. Reproduced with permission.^[^ ^57^ ^]^ Copyright 2014, Nature Portfolio. ii) Images of 200 mm^2^ microgels assembled via acoustic excitation. Reproduced with permission.^[^ ^58^ ^]^ Copyright 2011, Elsevier. iii) Image of microgels with varying sizes assembled into specific structures through high‐throughput light‐based manipulation. Reproduced with permission.^[^ ^59^ ^]^ Copyright 2017, Wiley.

Nondirectional packing methods, such as natural sedimentation, centrifugation, and vacuum‐assisted removal of the continuous phase, are widely used for jammed microgels.^[^ ^44^, ^49^ ^]^ Compared with the relatively loose packing achieved through natural sedimentation, centrifugation‐driven assembly of microgels results in larger contact areas and higher packing density.^[^ ^14g^ ^]^ For example, Britchfield et al. used centrifugation at 2000×*g* for 5 min to achieve the jammed granular hydrogel with 100% packing density and subsequently diluted the samples to obtain packing density of 80% and 60%.^[^ ^45^ ^]^ Accordingly, the porosity and rheological properties of the granular hydrogel scaffold will subsequently change with φ values, thereafter interfering with cell growth behaviors. As revealed by Jaberi et al., in the same GelMA granular systems, scaffolds subjected to higher centrifugal force and longer centrifugation duration (16 000×*g* for 300 s) exhibited lower porosity and higher *E*, reduced immediate penetration and migration length of NIH/3T3 murine fibroblast cells, and decreased metabolic activity after 7 d.^[^ ^50^ ^]^ This may be attributed to the deformation and compression of soft microgels, which likely fill the collapsed interstitial spaces between particles during centrifugation, decreasing the pore space drastically. Although cells can deform and theoretically squeeze through collapsed spaces, a previous study found that D1 mouse mesenchymal cells in scaffolds with higher particle fractions occupied less total volume and exhibited lower spreading.^[^ ^51^ ^]^ As displayed in **Figure** **2**a, in addition to centrifugal packing, vacuum treatment alone is also an option to achieve jammed granular hydrogels.^[^ ^52^ ^]^ Regardless of whether centrifugation or vacuum packing is used, the resulting granular hydrogels all exhibit strain‐yielding and self‐healing behavior. Furthermore, previous studies have also employed vacuum‐centrifugation combined treatment afterwards to complete particle filling and assembly. The formed granular hydrogels support rapid cell invasion after implantation into mouse TA muscle, and the invading cells include Pax7+ satellite cells, myogenic progenitor cells, eMHC+ multinucleated regenerating muscle fibers, CD68+ macrophages, and CD31+ endothelial cells and vasculature.^[^ ^27^ ^]^

#### 2.2.3 Directed Assembly between Microgels

Although the nondirectional packing of microgels is straightforward, it lacks control over the volume fraction and pore formation. For applications requiring more precise microstructures, such as tissue engineering, regenerative medicine, or cell/drug delivery carriers, micrometer‐level directional operations are necessary to achieve top‐down assembly of microgels and attain the desired structure.^[^ ^14^, ^60^ ^]^ In this process, various external driving forces with their own advantages and limitations are commonly employed such as surface energy, fluid force, magnetic force, and acoustic force (**Table** **1**). Depending on whether the assembly process relies on external energy input or precise regulation of external fields, these strategies can be categorized as passively driven assembly and actively driven assembly. Among them, passively driven assembly mainly relies on natural physicochemical interactions (e.g., surface tension and fluid shear force), enabling spontaneous assembly without external energy input, as observed in surface energy‐mediated and microfluidic‐guided assembly (Figure 2b,c). Specifically, surface energy‐mediated assembly drives the aggregation of hydrophilic microgels via surface tension at the liquid–liquid or liquid–air interfaces, minimizing system free energy to form higher‐order structures. For example, hydrophilic PEG microgels in a hydrophobic mineral oil phase have been successfully spontaneously transformed into linear, branched, random, and offset structures while preserving the activity of NIH/3T3 fibroblasts.^[^ ^54^ ^]^ Furthermore, to overcome the size limitations and improve efficiency of this strategy, more hydrophobic and dense organic solvents (e.g., perfluorodecalin) or optimized microgel shapes (e.g., lock‐and‐key designs) have been proposed to trigger tighter and more ordered packing.^[^ ^61^ ^]^ However, random or uncontrolled assembly at the micron scale may still be unavoidable, making it challenging to fabricate granular hydrogel structures with biologically relevant length scales using interface‐mediated strategies. To address this challenge, another passively driven strategy, microfluidic‐guided assembly, was developed (Figure 2c). This strategy utilizes the fluid forces generated by flow rate and pressure gradients to guide the precise movement and positioning of microgels. Complex assembled granular hydrogels with a delicate shape have been designed by using this technology (e.g., pyramids, Greek temples, Eiffel Tower, and skeletons).^[^ ^55^, ^56^ ^]^ Notably, a unique advantage of this strategy is that it allows the heterogeneous self‐assembly of microgels with different materials and cells.^[^ ^55^ ^]^ It has successfully developed 3D array‐structured granular hydrogels carrying multiple cell types (e.g., HepG2 and 3T3 cells), while ensuring a more than 99% cell survival rate under continuous culture medium flow after 24 h.^[^ ^62^ ^]^

**Table 1:** Merits and limitations of directed assembly of microgels.

External driving force	Merits	Limitations
Surface energy^[^ ^54^, ^61^, ^66^ ^]^	Simple and efficient; no complicated equipment or chemical reactions required; cell‐friendly; large‐scale manufacturing possibilities	Poor structural stability; Surfactant interference; Difficulty in obtaining multilayer granular hydrogel scaffolds
Fluidic force^[^ ^55^, ^56^, ^62^ ^]^	Simple and efficient; high precision; heterogeneity and versatility	Complexity of microgels (specific shapes, functionalization); Microfluidic equipment and channel design required; limited stability of microfluidic parameters (such as flow rate, pressure); poor structural stability
Magnetic force^[^ ^57^, ^63^ ^]^	Simple and efficient; dynamic adjustability; noncontact operation; high precision	Device complexity and limited material selection; size restrictions on assembly; high cost; potentially cytotoxicity
Acoustics force^[^ ^58^ ^]^	Noncontact operation; cell‐friendly; large‐scale manufacturing possibilities	Complex equipment requirements; poor structural stability
Light force^[^ ^59^ ^]^	High precision and programmability; localized assembly	Complex equipment requirements; photo‐responsive material requirements; poor structural stability

Actively driven assembly typically requires the use of energy or external fields (e.g., magnetic fields, sound waves, or light) to direct the packing of microgels (Figure 2d). For most strategies utilizing magnetic field, magnetic micro/nanobeads or free radicals (e.g., 4‐amino‐TEMPO) were incorporated into microgels to create magnetic field‐responsive smart particle building blocks.^[^ ^63^ ^]^ The iron oxide, trace amounts of other elements (e.g., nickel and cobalt), and free radicals involved in this aggregation process may lead to the risk of heavy metal poisoning and negatively impact cell viability and function. However, previous studies have reported that magnetic nanobeads did not show a significant impact on NIH/3T3 cell viability within 5 d, and antioxidants like vitamin E mitigated free radical toxicity to NIH/3T3 cells and rat cardiomyocytes.^[^ ^63^, ^64^ ^]^ In addition to magnetism, acoustic fields from acoustic stimulation can also directionally move PEG microgels, and assembly time increases with shape complexity (e.g., lock, Tetris, and zigzag shapes) without negative effects on NIH/3T3 cell viability.^[^ ^58^ ^]^ Besides, Dinh et al. used photothermal convection via localized surface plasmon resonance on gold nanorods to induce the aggregation of 1000 GelMA and PEGDA microgels within 2 min.^[^ ^59^ ^]^ However, the high‐power laser of this strategy may slow down the proliferation rate of bone marrow mesenchymal stem cells (hBMSCs).

Although directed assembly enables micron‐level control over the arrangement of microgels, currently nondirected packing is still the mainstream strategy. This is because, when granular hydrogels are used as micrometer‐scale scaffolds for cell culture or in vivo cell delivery, the precise ordering of microgels is not a critical factor. However, looking ahead to future applications in organoid development and biomimetic cellular microenvironments, directed assembly is advantageous because it enhances the structured organization of building blocks. To realize its full potential, the time and cost associated with these techniques must be carefully evaluated and optimized. Strategies such as acoustic, magnetic, and surface tension‐driven assembly theoretically possess a high potential for the large‐scale industrial production of granular hydrogels. Nevertheless, due to the inherently probabilistic nature of self‐assembly, these approaches generally exhibit limited precision and low yield. At present, millimeter‐scale aggregates (≈3–10 mm) can be formed within ≈5 s to 2 min using these low‐precision strategies.^[^ ^54^, ^64^, ^65^ ^]^ Importantly, parameters such as the secondary cross‐linking strategy and the size of the microgel building blocks must also be carefully considered when estimating assembly timescale and the resulting scaffold architecture. Different from low‐precision strategies, high‐precision guided self‐assembly approaches (e.g., microfluidic and microrobotic) offer improved spatial control but require specialized equipment, expert operation, and custom‐designed platforms.^[^ ^56^, ^57^ ^]^ These methods also depend on the large‐scale fabrication of chip‐based microcomponents or magnetic elements, which significantly increases manufacturing costs. Moreover, the number of microgels that can be manipulated simultaneously remains limited, falling well short of ensemble techniques capable of arranging millions of building blocks in parallel. This hinders their scalability for industrial applications. Currently, the spatially controlled assembly volume achievable by such precision techniques is typically restricted to below a few mm^3^, with overall operation time governed by the sequential positioning of individual microgels.^[^ ^56^ ^]^

#### 2.2.4 Secondary Cross‐linking between Microgels

**Figure 3:**
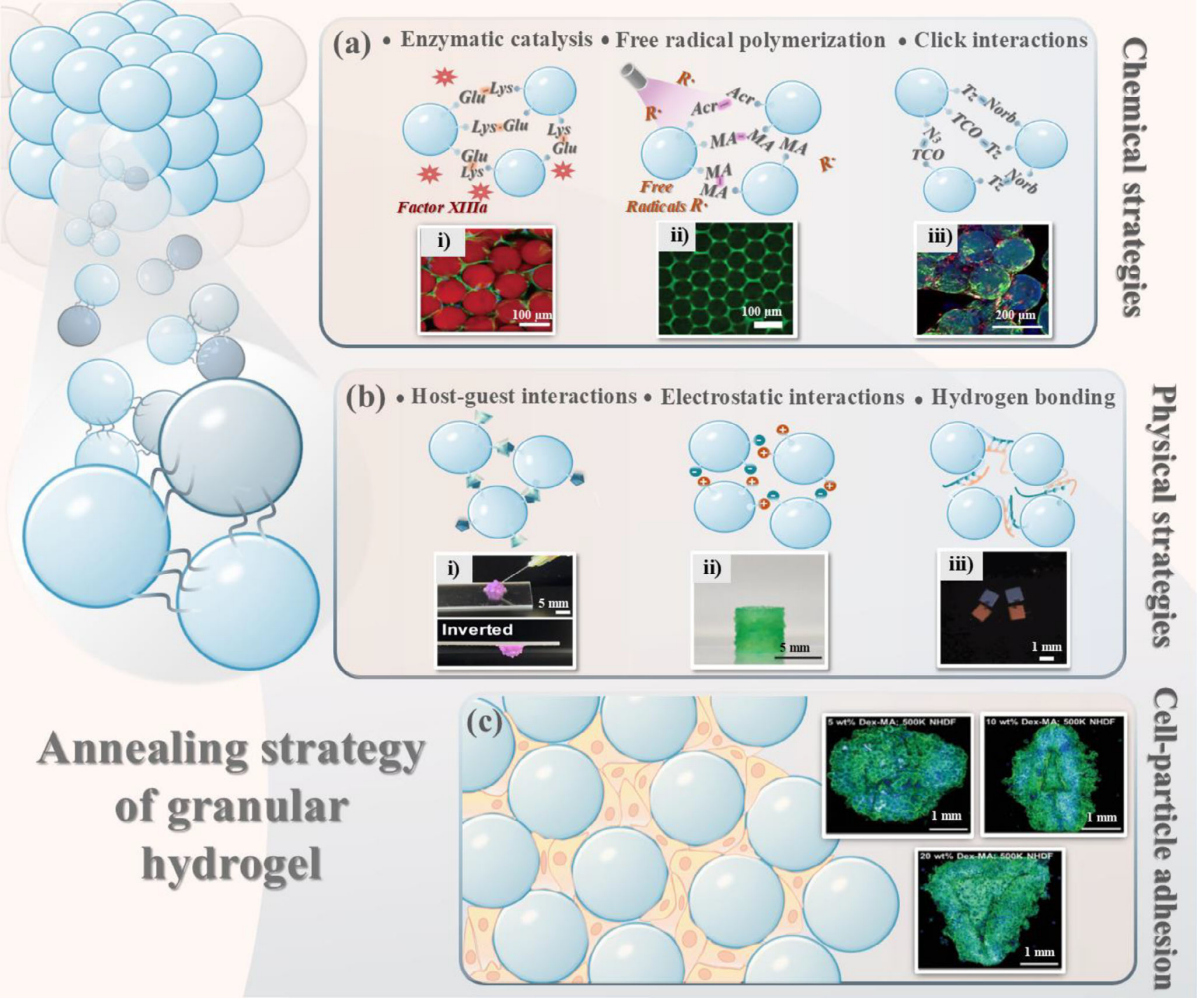
Overview of annealing strategies for granular hydrogels. a) Representative chemical annealing strategies, including enzyme catalysis, free radical polymerization, and click chemistry‐based interactions. i) Inset depicts enzymatic covalent bonding of microgels into cell‐laden scaffolds. Blue: nuclei; green: actin; red: PEG microspheres. Reproduced with permission.^[^ ^23a^ ^]^ Copyright 2019, Wiley. ii) Fluorescence image of FITC‐dextran‐labeled voids in a GelMA granular hydrogel formed via free radical annealing. Reproduced with permission.^[^ ^50^ ^]^ Copyright 2024, Wiley. iii) Click‐assembled gelatin–norbornene–carbohydrazide granular hydrogel for hMSC culture. Blue: nuclei; red: collagen I; green: fibronectin. Reproduced with permission.^[^ ^18a^ ^]^ Copyright 2024, Royal Society of Chemistry. b) Representative physical annealing strategies, including host–guest interactions, electrostatic interactions, and hydrogen bonding. i) Injectable granular hydrogels formed via β‐cyclodextrin‐adamantane complexation. Reproduced with permission.^[^ ^77^ ^]^ Copyright 2024, American Chemical Society. ii) 3D‐printed GelMA granular cylinders (5 mm) stabilized by electrostatic annealing using charged silicate nanoparticles. Reproduced with permission.^[^ ^23c^ ^]^ Copyright 2022, Wiley. iii) Red and blue hydrogel microcubes self‐assemble through complementary giant DNA strands. Reproduced with permission.^[^ ^73^ ^]^ Copyright 2013, Nature Portfolio. c) Schematic of cell‐adhesive granular hydrogels. Inset shows CLSM images of scaffolds formed with fixed microgel concentration with varying polymer concentrations (5, 10, and 20 wt%) after human dermal fibroblasts (NHDFs) culture. Reproduced with permission.^[^ ^76^ ^]^ Copyright 2024, Wiley.

Regardless of nondirected packing or directed assembly, only weak physical interactions exist between microgels, implying the scaffold structure of the formed granular hydrogel is typically unstable and easy to collapse over time. In this case, to obtain a stable interconnected structure, a secondary cross‐linking between microgels is necessary for fixation to ensure higher mechanical stability. This kind of granular hydrogel scaffold, formed by interconnected microgels, is commonly termed microporous annealed particle (MAP), where the “annealing” refers to the secondary cross‐linking process.^[^ ^14a^ ^]^ Based on the interaction forces, secondary cross‐linking strategies can be categorized into chemical reactions, physical interactions, and cell–particle adhesion (**Figure** **3**).

Chemical reaction‐driven HMP annealing relies on covalent bond formation between adjacent microgels. Such reactions often require functional group modifications on biomaterial molecular chains or on the surface of microgels. According to the functional modification, we basically categorize the popular chemical annealing strategy into enzyme‐catalyzed reactions, free radical polymerization, and click reactions (Figure 3a). The enzyme‐catalyzed annealing strategy commonly employs transglutaminase factor XIIIa to catalyze the covalent cross‐linking between transglutaminase peptides to complete the interconnection between microgels. Specifically, the formation of amide bonds occurs between lysine in K‐peptide and glutamine in Q‐peptide. Therefore, this strategy requires the modification of biomaterials with special peptides (K‐ and Q‐), introduced via thiol–ene click reaction (Table S1, Supporting Information). Mild annealing conditions, i.e., neutral pH and physiological temperature, allow the enzyme‐catalyzed method to meet the requirement for the co‐assembly of microgels and living cells. Another common annealing strategy is free radical polymerization, which requires unsaturated double bonds on the microgel materials and free radical initiators. Currently, the most widely used reactive double bond groups include methacrylate groups, acrylate groups, and maleimide groups, which have been successfully applied to the cross‐linking of PEG, HA, gelatin, and alginate microgels.^[^ ^14^, ^24^, ^41^, ^67^ ^]^ To further enhance the annealing efficiency of free radical polymerization, synthetic biomaterials with a higher density of unsaturated double bonds or multi‐arm structures (e.g., 4‐arm or 8‐arm PEG) can be selected to enhance cross‐linking sites.^[^ ^68^ ^]^ Although the free radical polymerization strategy possesses advantages of shorter reaction times and higher spatiotemporal resolution, the potential negative impact of free radicals generated during the process on cell activity cannot be overlooked. Another common chemical annealing strategy for microgels is the conduction of highly selective and high‐yield click chemistry reactions, including thiol–ene click chemistry, azide–alkyne cycloaddition (AAC), inverse electron demand Diels–Alder (iEDDA) reactions, and vinylsulfone–amine click chemistry.^[^ ^14^, ^68^, ^69^ ^]^ Therefore, the functional groups that trigger click reaction, such as azide, Norb, *trans*‐cyclooctene (TCO), and tetrazine (Tz) groups, must be pre‐grafted onto the biomaterials. As shown in Table S1 (Supporting Information), most functional groups are initially employed to conduct the covalent gelation of microgels, and the remaining unreacted groups on the microgel surface are subsequently utilized for annealing. In certain cases, some studies have also focused on the separate preparation of two types of microspheres or crosslinker, each grafted with click‐chemistry reactive groups and their corresponding pairing groups.^[^ ^14a,g^ ^]^ For example, Hu et al. demonstrated the annealing of granular hydrogels by mixing alginate microspheres modified with Tz with those modified with Norb or TCO, revealing a higher assembly efficiency of Tz‐TCO click reaction.^[^ ^14g^ ^]^

In contrast to the rapid, highly selective, and irreversible chemically induced annealing strategies, most physical annealing strategies are more spontaneous and dynamically reversible, supporting the unique properties of MAP, such as self‐healing, injectability, and truncation. As demonstrated in Figure 3b, popular physical annealing strategies currently include host‐guest interactions, electrostatic interactions, and hydrogen bonding.^[^ ^14^, ^70^ ^]^ Specifically, the assembly process initiated by host–guest interactions involves the grafting of host groups (e.g., α‐, β‐, γ‐cyclodextrin, calixarene) and guest groups (e.g., adamantane, linear alkyl chains) onto the surface of different microgels. By adjusting the size and shape of the host hydrophobic cavity and the guest ligand, supramolecular host–guest assortment between microgels can be selectively performed to obtain centimeter‐scale granular hydrogels.^[^ ^23b^ ^]^ Given the weak and unstable nature of host–guest interactions, some studies have chosen to coordinate this technique with other chemical annealing strategies to facilitate secondary cross‐linking.^[^ ^71^ ^]^ Unlike host–guest interactions, which rely on steric matching and chemical compatibility, electrostatic interactions are primarily driven by mutual attraction between opposite charges. Most strategies are based on the simple mixing of different microgels carrying positive and negative charges to obtain annealing through electrostatic adsorption.^[^ ^23^, ^72^ ^]^ By carefully selecting the relative sizes of microgels, the well‐defined core–satellite structures of annealed microgel can be assembled via electrostatic actuation.^[^ ^70b^ ^]^ Furthermore, the surface charge of microgels has been confirmed to have no significant effect on the viability of Schwann cells (SCs), human adipose‐derived stem cells (hADSCs), or fibroblasts, while supporting their migration and proliferation.^[^ ^18b^ ^]^ For higher biocompatibility, milder hydrogen bond induction strategies can be considered, which are mostly dependent on DNA base complementarity and peptide self‐assembly. For example, by modifying the surface of cubic microgels with short DNA primers and subsequently pairing giant DNA by in situ rolling circle amplification, Qi et al. successfully induced the annealing of microgels across scales.^[^ ^73^ ^]^ Subsequently, Merindol et al. prepared a series of all‐DNA particles and further created either dense structural scaffolds or loose core‐satellite scaffolds by varying the mass concentration and the ratio of particles with complementary sequence.^[^ ^74^ ^]^ As expected, during the DNA hybridization‐guided annealing, both 3T3 fibroblasts and A549 lung adenocarcinoma cells exhibited high survival rates (>90%) within the microgels, where 3T3 cells demonstrated expansion and adhesion, while the A549 cells formed 3D tumor spheres.^[^ ^74^ ^]^

Although physical annealing is more flexible and reversible, the crosslinked granular scaffolds still process a relatively static structure, lacking the dynamic and self‐organization capacity in natural ECM. To overcome this limitation, microgel aggregation driven by cell–cell and cell–matrix interactions has been proposed and has gained widespread popularity in organoid culture. The cell‐driven annealing strategy favors bio‐responsive materials, such as collagen, chitosan, HA, and gelatin, because they provide sufficient adhesion sites for cell attachment and proliferation.^[^ ^24^, ^75^ ^]^ Recent studies have also employed dextran functionalized with RGD peptides to produce microgels, and physically adhered them together through cell migration and stretching.^[^ ^76^ ^]^ Obviously, the assembly of the cell–particle adhesion strategy depends largely on cell type and seeding density, as well as on microgel shape and rigidity, therefore lacking the efficiency and precision offered by chemical or physical strategies.^[^ ^24^, ^76^ ^]^ Furthermore, the cell‐to‐microgel ratio significantly influences the shape of the final granular hydrogel. As shown in Figure 3c, a lower cell concentration tends to result in a flatter, oval‐shaped 3D structure, whereas a higher cell concentration leads to a triangular 3D structure, with cells beginning to stack and grow upward.^[^ ^76^ ^]^ However, since this strategy is driven spontaneously by cell adhesion and does not require additional chemical functionalization or annealing treatments (e.g., UV exposure, free radicals, or organic reagents), it possesses high biocompatibility and minimizes toxicity.

## 3 Effects of Overall Granular Hydrogel Properties on Cell Fate

Given the broad application of assembled granular hydrogels in cell loading and tissue engineering, it is crucial to identify how the diverse physicochemical properties of overall granular hydrogels affect the cell growth microenvironments, and eventually, the cell fate. These final physicochemical properties include porosity, mechanical properties, degradability, heterogeneity, and drug loading, which coordinately determine the fate of cells within the granular matrix. In addition, the cell loading strategy, although not an intrinsic property of aggregated granular hydrogel, also affects the subsequent cell growth and behavior. In this section, we elaborate on how to fine‐tune these properties of granular hydrogels to create an optimal cellular environment.

### 3.1 Porosity

The porosity of granular hydrogels is a complex property synergistically determined by the morphology and mechanical characteristics of microgels as well as by the packing density and external forces between microgels. In certain in vivo conditions, porosity may also change due to the swelling of the particulate hydrogel upon exposure to interstitial fluid. Currently, common strategies for regulating porosity include altering microgel size and aspect ratios, adjusting microgel rigidity, modifying centrifugal force in the jamming process, tuning annealing strategy, and changing the concentration of the annealing agent.^[^ ^33^, ^42^, ^44^, ^79^ ^]^ As mentioned in Section 2.2.2, low porosity resulting from high packing density can inhibit cell migration and metabolic activity. Theoretically, cells may still be able to squeeze through crowded inter‐void spaces of microgels. However, a recent study demonstrates that MDA‐MB‐231 cells within compact pore spaces adopt a highly deformed, narrow, and elongated morphology, characteristic of cell behavior in confined microenvironments.^[^ ^50^ ^]^ This suggests that as the pore size of the granular hydrogel decreased, cell spreading mechanism transitions from 2D surface‐like diffusion to 3D encapsulation‐like diffusion, emphasizing the control of porosity on cell fate in granular hydrogel systems.

**Figure 4:**
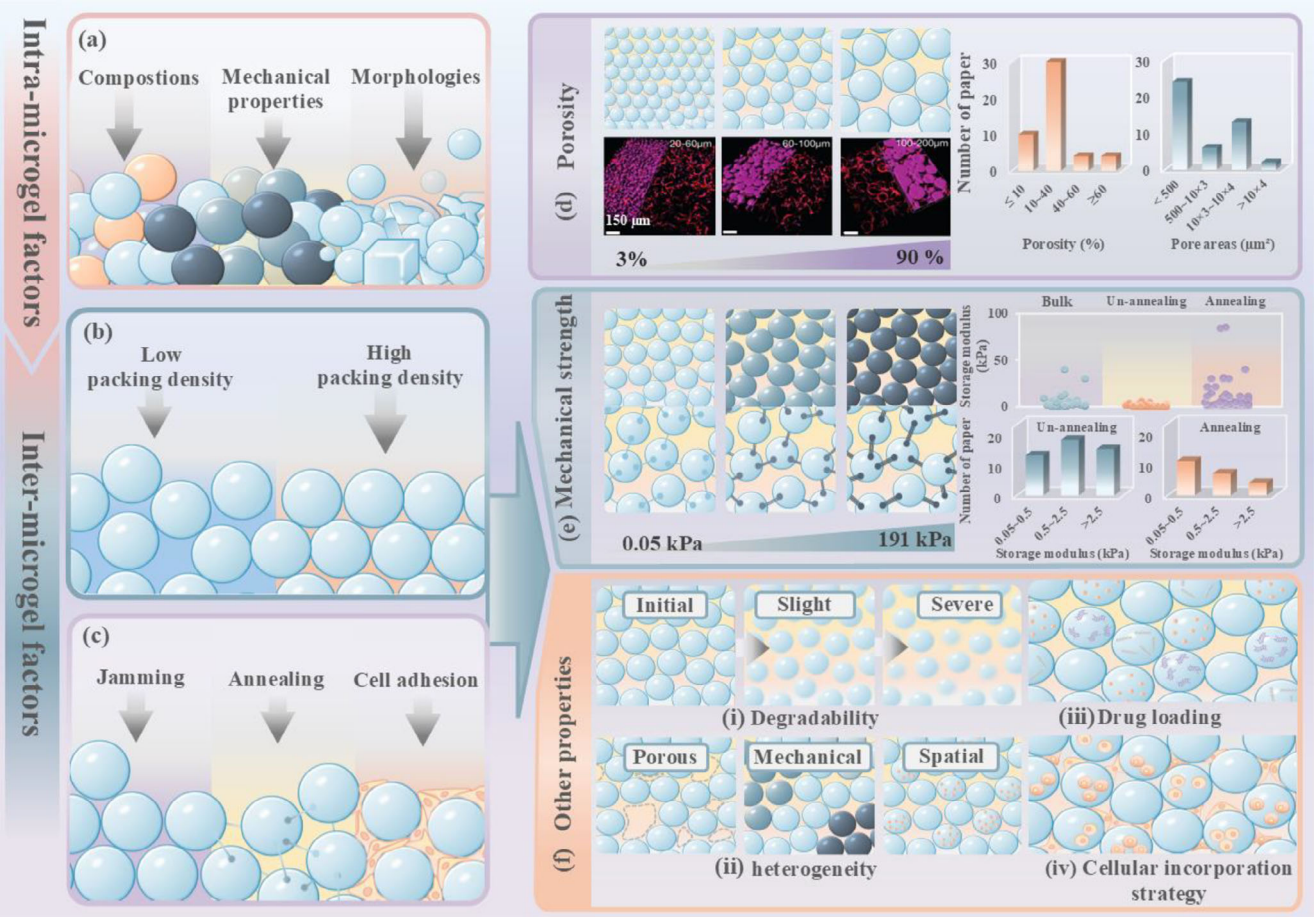
Intra‐/inter‐microgel factors collectively determine granular hydrogel properties. Schematic of a) intra‐microgel factors and b,c) inter‐microgel factors influencing hydrogel properties. d) Visual representation of small, medium, and large microgels with corresponding pore size ranges. Inset: 3D rendering of human dermal fibroblasts (HDFs) spreading on granular hydrogels with varying microgel sizes. Pink: microgels, Red: actin, Blue: nuclei; scale bar = 150 µm. Reproduced with permission.^[^ ^78^ ^]^ Copyright 2019, Elsevier. Bar charts summarize porosity and pore areas in studies over the past 5 years (Table S3, Supporting Information). e) Schematic of tuning granular hydrogel mechanics by assembling microgels with varying stiffness or by secondary cross‐linking, with pore size ranges based on data from Table S4 (Supporting Information). Scatter plot shows storage moduli of bulk, unannealed, and annealed granular hydrogels reported over the past 5 years. Bar chart summarizes studies using granular hydrogels (with/without annealing) across storage modulus ranges: 0.05–0.5, 0.5–2.5, and >2.5 kPa. f) Schematic diagrams of other granular hydrogel properties: i) Progressive degradation over time, from slight to severe. ii) Illustrations of pore, mechanical, and spatial heterogeneity. iii) Functionalization of granular hydrogels with various biological cues (gray: proteins; orange: small molecules; purple: genetic materials). iv) Strategies for incorporating different cell types (varied colors represent distinct cell types) into the granular hydrogel matrix.

By reviewing over 20 studies from the past 2 years that provided porosity data, we found that most studies prefer to use microgels with a size of 40–90 µm to prepare granular hydrogel scaffolds with a porosity of 3%–50% (**Figure** **4**a). The pore space area is concentrated between 100 and 1000 µm^2^, which is directly related to the microgel size. Widener et al. recently classified granular hydrogels into small‐pore (1–22 µm), medium‐pore (22–54 µm), and large‐pore (>54 µm) systems based on their average pore size, identifying their specific effects on immune cellular responses.^[^ ^10^ ^]^ Based on their findings, we further summarize the porosity thresholds that drive the transition of cell fate from 2D‐ to 3D‐like behavior. Based on our summary, when the porosity is less than 10%, corresponding to an empty area of less than 100 µm^2^, the migration of 3T3 cells and primary human chondrocytes is significantly restricted, leading to sparse cell distribution and an inability to form an interconnected network.^[^ ^50^, ^80^ ^]^ HUVECs have limited proliferation due to insufficient pores and mainly exhibit a compact spherical morphology with limited cell extension and flattening.^[^ ^81^ ^]^ Similarly, hMSCs exhibit a more rounded morphology with elongated ciliary processes, while their cytoskeleton displays a diffuse actin distribution and lacks distinct fibrous structures.^[^ ^69a^ ^]^ At this porosity, macrophages become more compact, with reduced surface area and volume, and exhibit a tendency toward M1 polarization, resembling their behavior in nanoscale hydrogel 3D culture systems.^[^ ^82^ ^]^ When porosity increases to the range of 10%–40%, cells can gradually migrate into the scaffold, forming smaller‐scale networks, as observed with MSCs, mouse L929 cells, and human dermal fibroblasts (HDFs).^[^ ^31^, ^79^ ^]^ The enhanced pore connectivity supports the efficient spreading and high‐density distribution of hBMSCs, particularly when supplemented with RGD peptides and growth factors such as PDGF‐BB and TGF‐β3.^[^ ^83^ ^]^ HUVECs cultured in granular hydrogels with 25% porosity exhibited significantly enhanced vascular sprouting, forming longer and denser capillary networks.^[^ ^47^ ^]^ Meanwhile, macrophages exhibit mixed M1/M2 polarization characteristics, with increased CD206 expression, and demonstrate a notable capacity to promote tissue repair.^[^ ^29^ ^]^ This observation suggests that porosity in the 10%–40% range may represent the transition zone between 3D‐ and 2D‐like cellular behavior. When porosity increases further to 40%–60%, it optimally supports cell expansion, proliferation, and matrix secretion in primary human chondrocytes, enhancing the secretion of glycosaminoglycans (GAGs) and collagen.^[^ ^60a^ ^]^ Macrophages were able to stretch and move freely, predominantly exhibiting M2 polarization, while showing low expression of antigen presentation markers such as CD11c, indicating a complete transformation of 2D‐like behaviors.^[^ ^83^ ^]^ When porosity exceeded 60%, endothelial cells and fibroblasts were widely distributed throughout the scaffold, exhibiting deep penetration and effective cell–cell interactions, while the frequency of material–cell interactions decreased.^[^ ^31^, ^42^ ^]^ Overall, an appropriately small porosity (10%–40%) ensures that the pores in the scaffold remain relatively uniform and interconnected, facilitating rapid cell migration and quickly forming a 3D network. In contrast, when the granular structure is too loose (porosity >40%), cells may only interact with a few individual microgel surfaces, rather than receiving stimulation from all spatial directions simultaneously. This implies that material‐cell interactions within the system are limited, causing cells to aggregate into larger clusters, which hinders uniform cell distribution.

### 3.2 Mechanical Properties

The mechanical strengths of jammed and annealed granular hydrogels, along with bulk hydrogels of identical formulation, are compared in Figure 4e. As expected, jammed granular hydrogels exhibit lower storage modulus (*G*′) values, typically ranging from 0.5 to 8.0 kPa. In jammed state, the mechanical properties of granular hydrogels are primarily influenced by the stiffness and contact area of individual microgels. Regarding stiffness, increasing the *E* of individual microgels generally enhances the overall stiffness of the granular scaffold. However, this relationship is not strictly linear.^[^ ^84^ ^]^ Under large deformations, microgels with high *E* exhibit limited deformation and lower energy dissipation, resulting in a lower overall *G*′ of formed granular hydrogels compared with that of hydrogels composed of medium‐hard microgels.^[^ ^84a^ ^]^ In addition to stiffness, the contact area, which is governed by microgel packing density and morphology, also plays a critical role in determining the mechanical strength of granular hydrogels in jammed state. It has been reported that high packing density significantly increases the *G′* of granular hydrogels, eventually reaching a saturation point at an ultra‐high filling state.^[^ ^50^, ^84^ ^]^ In addition, when the *E* of the microgel exceeds 120 kPa, the contact area between microspheres increases with microgel size, enhancing the *G′* of granular hydrogels. Conversely, relatively soft microgels (*E* = 20 kPa) undergo greater deformation during filling, altering particle contact modes and offsetting the effect of microsphere size.^[^ ^84b^ ^]^

As illustrated in Figure 4e, after annealing treatment, the mechanical strength of loosely packed granular systems generally increases beyond the original *G*′ of the jammed microgels, ranging from 0.15 to 39.00 kPa. The annealed granular scaffolds exhibited a *G*′ distribution comparable to that of bulk hydrogels. This indicates that, despite the presence of micrometer‐scale pores, the strong inter‐microgel interaction forces can effectively offset the adverse effects of porosity on the overall mechanical strength. Different annealing strategies may also lead to different stiffness in the final granular hydrogel. For example, with the same PEG (Mw = 5 kDa) microgels, MAPs annealed via the iEDDA reaction exhibited a 1.9‐fold higher *G*′ than scaffolds annealed using the thiol–ene click reaction.^[^ ^28^ ^]^ This is because the Tz‐Norb reaction not only provides primary cross‐linking points but also introduces secondary interactions (e.g., π–π stacking, hydrogen bonding, or van der Waals forces) of dihydropyrazine rings, further enhancing the mechanical properties of the granular systems. More details on various annealing strategies used to improve the mechanical properties of MAP can be seen in the review by Charlet et al.^[^ ^85^ ^]^

According to Figure 4, we categorized the *G*′ of annealed granular scaffolds into three ranges: low modulus (<1 kPa), medium modulus (1–2 kPa), and high modulus (>2 kPa). Different cell types exhibit varying requirements for the mechanical properties of granular hydrogel scaffolds. Specifically, when *G*′ < 1 kPa, hMSCs and C3H/10T1/2 fibroblasts exhibit lower adhesion and proliferation abilities, but this range is more suitable for HDFs to spread, proliferate, and reshape the local environment.^[^ ^28^, ^86^ ^]^ At this range, macrophages tend to polarize toward the M1 phenotype, secreting pro‐inflammatory factors such as TNF‐α, while the osteogenic differentiation of BMSCs is unfavorable.^[^ ^87^ ^]^ When *G′* rises to 1–2 kPa, the higher rigidity of the scaffold inhibits the spreading and proliferation of HDFs, but hMSCs were stretched and secreted more ECM components (including collagen and glycosaminoglycans).^[^ ^28^, ^86^, ^88^ ^]^ Individually, some studies have also shown that hMSCs exhibit low adhesion ability in granular systems ˂2 kPa, with round cell morphology and limited ECM secretion and migration.^[^ ^18a^ ^]^ When the *G'* exceeded 2 kPa, hMSCs and BMSCs exhibited enhanced proliferation, migration, and functional differentiation, along with increased expression of osteogenic differentiation markers such as ALP activity and BMP‐2 secretion.^[^ ^28^, ^87^ ^]^ Meanwhile, macrophages were driven toward M2 polarization, leading to increased secretion of IL‐10 and BMP‐2 while reducing neurotoxic inflammation.^[^ ^60b^ ^]^ The above results suggest that a medium modulus (1–2 kPa) is better suited for dynamic tissue remodeling, whereas a higher modulus (>2 kPa) is more effective in promoting specific functional differentiation of cells.

It is worth noting that the above behavioral characterizations are based on cases where cells are seeded in the gaps between microgels. They are susceptible to mechanical stimulation on multiple scales, not only from the nearest signal microsphere but also adjacent microgels. Combined with the pore size range summarized in Table S3 (Supporting Information) (≈5–380 µm), cells in granular scaffolds should theoretically be able to sense stimuli across all spatial direction, similar to 3D cultures, but at the micrometer level. Thus, the overall mechanical property of the annealed granular hydrogel plays a dominant role rather than the local mechanical property of the single microgel. However, as previously mentioned, if the pore area is too large, cells may only experience mechanical stimulation from the nearest microsphere, resembling a 2D culture system. In addition, the case where cells are encapsulated in microgels aligns more closely with a 3D culture system, and therefore, we will not elaborate on this aspect here.

### 3.3 Degradability

The degradability of granular hydrogels has a significant impact on cell infiltration and remodeling and drug release behavior within the matrix. The degradation behavior of granular hydrogels primarily depends on the composition of the microgel, the cross‐linking network characteristics within the microgels, and the annealing cross‐linking applied between the microgels. Commonly used biomaterials, such as HA and gelatin, possess endogenous enzyme sensitivity, allowing their degradation rates to be easily regulated by enzymes (e.g., hyaluronidase and collagenase) in the biological environment.^[^ ^18^, ^89^ ^]^ For nondegradable synthetic materials like PEG, many studies introduce enzyme‐sensitive peptides, such as MMP‐sensitive peptide sequences, to regulate the degradation rate of granular hydrogels via the cleavage of specific peptide segments by MMPs secreted by cells. For example, Recalde Phillips et al. achieved both fast and slow degradation of MAP annealed via the iEDDA reaction by grafting MMP‐sensitive peptides with different sensitivities (e.g., KCGPQGIWGQCK and CGPQGPAGQGCR) onto PEG.^[^ ^28^ ^]^ Interestingly, Shi et al. created MAP with a degradation gradient by adjusting the ratio of MMP‐sensitive crosslinkers to nondegradable PEG‐dithiol crosslinkers.^[^ ^49^ ^]^ They found that when the proportion of MMP‐sensitive peptides was at or above 50%, hMSCs exhibited enhanced expansion and network formation capabilities. Below this ratio, the degradation performance of the hydrogel decreased significantly, limiting the range of cell activity and expansion behavior, showing an isolated distribution. Furthermore, the rate of enzymatic degradation is naturally dependent on enzyme concentration. Under high enzyme concentration conditions, the degree of degradation (mass loss) is positively correlated with time, and the degradation‐induced pore enlargement provides more space for the migration and expansion of hMSCs and significantly enhances the sprout‐like invasion behavior of HUVECs.^[^ ^90^ ^]^ In addition to enzyme‐sensitive degradation, scaffolds can also degrade through hydrolysis. For instance, the degradation rate of the system can be accelerated by increasing the number of ester‐containing crosslinks in PEG‐maleimide microgels.^[^ ^91^ ^]^

The cross‐linking density of microspheres is another key factor influencing the degradation time of granular hydrogel scaffolds, implying that the degradation behavior of the matrix is closely linked to its mechanical properties. In other words, the hardness of the microgels determines its degradation rate, and the degradation process, in turn, softens the microgels, leading a coordinated mechanical‐dependent immune response and tissue reorganization. For example, MAP annealed from low‐concentration GelMA (5%) microgels with a *G'* of 20 ± 3 kPa was completely degraded within 7 d, while MAP composed of high‐concentration GelMA (20%) microgels with a *G'* of 39 ± 9 kPa exhibited an extended degradation time of over 14 d.^[^ ^86b^ ^]^ During the degradation induced by collagenase, when the mass loss of granular hydrogels containing MMP‐sensitive peptide sequence crosslinkers reached 33%, the *G'* decreased by ≈90%. The significant decrease in *G'* during rapid degradation further increased the number of hMSCs by about fourfold within 8 d, induced the cells to secrete higher levels of ECM proteins (e.g., collagen and fibronectin), and increased the secretion of vascular endothelial growth factor (e.g., VEGF) and bone morphogenetic protein (e.g., BMP‐2).^[^ ^92^ ^]^ In contrast, softened granular hydrogels after degradation (reduced from 20 to 8 kPa) inhibited the polarization of pro‐inflammatory macrophages (M1) and promoted the anti‐inflammatory macrophage (M2) phenotype, while reducing the recruitment of CD4+ T cells and the secretion of inflammation‐related cytokines (IFN‐γ and IL‐2) in vivo.^[^ ^91^ ^]^ Interestingly, a recent study regulated the mechanical compression process and cellular behavior of HA microgel‐MSC spheroid granular hydrogels by controlling the microgel degradation rate. The results revealed that rapidly degrading microgels enhanced ECM deposition, which serves as a key driving force for cell compression.^[^ ^93^ ^]^ In addition to mechanical strength, the degradation behavior of granular hydrogels is also influenced by the annealing strategy. For example, Recalde Phillips et al. found that PEG scaffolds annealed via the iEDDA reaction exhibited greater resistance to degradation than those annealed using the thiol–ene click reaction.^[^ ^28^ ^]^ Under collagenase treatment, the latter completely degraded after 105 min, whereas the former retained 50% of their mass.

### 3.4 Heterogeneity

Cellular growth behavior during development, homeostasis, and injury response can be controlled by combinations of increased inter‐granular heterogeneity. As discussed in Section 2, individual microgels can achieve heterogeneity in various aspects, including hydrogel formulation, chemical modification, morphology, and mechanical properties. During the manufacturing process of granular hydrogels, macroscopic anisotropy, including mechanical, pore, and spatial heterogeneity, can be achieved by mixing and assembling multiple microgel populations in either layer‐by‐layer or random distributions. For mechanical heterogeneity, microgel units with varying mechanical properties (hard and soft) have been layered before annealing to create an MAP system with a stable stiffness gradient, and hMSCs have been found to show continuous changes along the stiffness gradient.^[^ ^23a^ ^]^ The same strategy can be applied to produce MAP hydrogels with a degradation gradient.^[^ ^49^ ^]^ Regarding pore heterogeneity, a common approach involves mixing sacrificial microgels with nonsacrificial microgels, and the porosity is then adjusted by selectively dissolving the sacrificial microgels, thereby controlling conditions for cell growth. For example, Seymour et al. created scaffolds with tunable porosity and uniform cell distribution by randomly mixing structural GelMA microgels with sacrificial, cell‐encapsulating oxidized alginate microgels.^[^ ^81^ ^]^ Of course, porosity heterogeneity can also be achieved through the gradient assembly of microgels with small, medium, and large particle sizes, which induce different appearance and expansion of adipose‐derived stem cells (ADSCs).^[^ ^18c^ ^]^ For spatial tissue heterogeneity, previous studies have preferred to mix microgels carrying different bioactive molecules in specific proportions to create heterogeneous scaffolds, effectively guiding cells to grow along defined paths. Specifically, Pruett et al. designed a heterogeneous MAP scaffold containing heparin microislands grounded in agent‐based modeling (Hybrid ABM).^[^ ^94^ ^]^ In this system, heparin confined within the microgels guided the migration and angiogenesis of endothelial cells by adsorbing and releasing growth factors (such as VEGF). The optimal angiogenic response was achieved at a heparin microisland ratio of 26%. A similar strategy was employed to design sulfated hyaluronic acid methacrylate microislands, which used the negative charge of sulfate to capture positively charged growth factors (such as TGF‐β3 and PDGF‐BB), enabling the directional migration of hMSCs as well as promoting chondrogenesis.^[^ ^83^ ^]^ Interestingly, Emiroglu et al. recently took advantage of the modular properties of granular hydrogel by combining IL‐6 chelating microgels with VEGF‐releasing microgels to achieve capture of inflammatory cytokines and release of pro‐regenerative factors simultaneously, effectively promoting angiogenic activity.^[^ ^60c^ ^]^ Such spatial heterogeneity by separating functional and nonfunctional regions will achieve spatial control of cell migration and provide more possibilities for fine regulation in tissue engineering and regenerative medicine.

### 3.5 Drug Loading

Microgels have been designed for controlled drug delivery, providing temporal and spatial control over the release of various therapeutic ingredients or biological cues. Subsequently, the random or intentional assembly of microgel units loaded with different biological signals or functional components can generate functional drug‐loaded granular scaffolds. One loading strategy of microgels is to use straightforward noncovalent interactions without the requirement of complex chemical modifications or linkages, including physical entanglement and electrostatic interactions.^[^ ^79^, ^83^, ^88^, ^89^, ^95^ ^]^ In this case, the release rates of these biological cues can be regulated by modifying the composition, concentration, and cross‐linking density of the matrix precursor within the microgels.^[^ ^9^ ^]^ Recently, D'Elia et al. achieved controllable loading of bovine serum albumin (BSA) and cytokines (IL‐4, IL‐10, and IFNγ) onto β‐cyclodextrin granular hydrogel scaffolds through guest–host complexation by attaching adamantane groups to the biomolecules.^[^ ^77^ ^]^ The affinity of the adamantane conjugates can be directly adjusted by varying the number of attached guest groups, resulting in a more than twofold reduction in IL‐4 and IL‐10 release over 14 d, meanwhile sustaining modulation of macrophage phenotype by inhibiting pro‐inflammatory markers (Nos2) and promoting the expression of anti‐inflammatory markers (Arg1). Another incorporation strategy of microgels involves covalent cross‐linking, achieved through specific click chemistry reactions to ensure stoichiometric control of the functional ingredients attached to the surface or interior of microgels such as thiol–ene chemistry, strain‐promoted AAC, and the iEDDA reaction.^[^ ^60^, ^68^, ^96^ ^]^ With covalent cross‐linking, the release behavior of the cargo can be precisely controlled by regulating the density of reactive groups and the number of affinity ligands (e.g., heparin–thiol) incorporated into the microgel composition.

As shown in **Table** **2**, the functional biological cues incorporated into the scaffold matrix mainly include proteins, peptides, small molecules, genetic materials, and various nanoparticles.^[^ ^68^, ^70^, ^77^, ^87^, ^96^, ^97^ ^]^ Among them, protein‐based biological signals can be basically divided into cell growth factors and inflammatory modulation molecules. Specifically, growth and differentiation regulatory factors, such as VEGF, transforming growth factor β (TGF‐β), and recombinant human bone morphogenetic protein 2 (rhBMP‐2), are commonly employed to stimulate MSC proliferation and induce their differentiation into the phenotypes required for regeneration. Thereafter, the capacity of granular hydrogels to support blood vessel formation is enhanced, promoting bone tissue development in tissue repair applications.^[^ ^28^, ^60^, ^83^, ^89^ ^]^ Meanwhile, neurotrophic factors, such as glial cell‐derived neurotrophic factor (GDNF), brain‐derived neurotrophic factor (BDNF), and nerve growth factor (NGF), are generally applied to regulate the neural differentiation of MSCs or neural progenitor cells (NPCs), improving the outgrowth and length of the neurite and enhancing the efficiency of neural network reconstruction.^[^ ^18^, ^97^ ^]^ Some laminin‐derived peptides (e.g., IKVAV and YIGSR) can also affect the differentiation behavior of NPCs.^[^ ^96a^ ^]^ Unlike cell growth factors, inflammatory modulation molecules regulate immune cell behavior such as the polarization of macrophages. These inflammatory cytokines can alter the phenotypes of macrophages, induce desired gene expression responses, and further promote local tissue repair.^[^ ^68^, ^77^, ^96^ ^]^ In addition, previous studies have also driven the directional migration of responding cells and enhanced the migration ability of cells by regulating the concentration gradient of these biological signals (e.g., CXCL1, VEGF‐A).^[^ ^60^, ^79^ ^]^

**Table 2:** Summary of drug‐loaded granular hydrogels over the past 5 years.

Category	Drugs loaded	Cells loaded	Biomedical applications	Ref.
Proteins and peptides	Human platelet lysate (PL) proteins	Human mesenchymal stem cells (hMSCs)	Microporous scaffolds with sustained delivery of PL proteins for functional tissue repair	[89]
	Glial cell‐derived neurotrophic factor (GDNF); Brain‐derived neurotrophic factor (BDNF)	Neural progenitor cells (NPCs)	Granular hydrogels supporting long‐term neural culture with extensive neurite outgrowth	[97a]
	Nerve growth factor (NGF)	Schwann cells (SCs); human adipose‐derived mesenchymal stem cells (hADSCs); Fibroblasts	An injectable granular matrix with interconnected pores and propagates gradient NGF for spontaneous assembly into a complex shape	[18b]
	Recombinant human bone morphogenetic protein 2 (rhBMP‐2)	None	Implantable granular scaffolds loaded with rhBMP‐2 for repairing skull defects in mice	[28]
	Chemokine	Human dendritic cells (DCs);Human gastric organoids (HGOs)	Granular hydrogel scaffolds providing mechanical support for the culture of HGOs and the co‐culture of DCs and HGOs	[79b]
	Bovine serum albumin (BSA) and cytokines	Bone marrow‐derived macrophages (BMDMs)	Affinity‐based protein delivery system for sustained local presentation of biomolecules	[77]
	Interleukin‐33 (IL‐33)	Murine macrophage line RAW 264.7	Bioactive material based on IL‐33 coupled to a granular scaffold for stimulating macrophages in vitro and regulating immune cell recruitment to the implant site in vivo	[68b]
	Interleukin‐10 (IL‐10)	THP‐1 cells; hMSCs	IL‐10‐modified microgel scaffolds for macrophage culture, promoting tissue regeneration and wound repair	[96b]
	Positively charged growth factors	Human bone marrow mesenchymal stem cells (hBMSCs); Chondrocytes	Injectable, growth factor‐loaded heterogeneous granular hydrogels serving as scaffolds for cartilage extracellular matrix deposition	[83]
	Antigens	None	Novel antigen granular delivery platform designed for sustained and prolonged antigen release	[99]
	Laminin‐derived peptides	NPC	Microporous annealed particle scaffolds incorporating neurogenic peptides for the culture of NPC in stroke treatment	[96a]
Genetic material	Cytosine guanosine oligonucleotide	None	Granular hydrogel system encapsulating liquid nitrogen‐inactivated tumor cells and immunostimulants used to continuously recruit various immune cells for postoperative cancer treatment	[97d]
Proteins and genetic material	Vascular endothelial growth factor A (VEGF‐A); Interleukin‐6 (IL‐6) aptamers	None	Granular systems functionalized with VEGF‐A and IL‐6 to locally modulate the relative concentrations of pro‐ and anti‐inflammatory factors for chronic wound regeneration	[60c]
Small molecules	Thiolated heparin	Human dermal microvascular endothelial cells (HDMVECs)	Heparin‐modified heterogeneous granular hydrogel for guiding endothelial cell migration	[94]
	Thiolated heparin	None	Injectable granular hydrogel material with heparin micro‐islands for vocal cord augmentation	[97c]
	Aminooxyacetic acid (AOA)	H9C2 cardiomyocytes; T cells	ROS‐responsive granular hydrogel releasing the prodrug AOA to restore cardiac function and significantly reduce myocardial fibrosis in vivo	[70a]
	Simvastatin (SIM)	BMSCs	Injectable simvastatin‐loaded cartilage acellular matrix granular system for microfracture articular cartilage regeneration	[100]
Nanoparticle	Poly(lactic‐*co*‐glycolic acid) nanoparticle (PLNA) loaded with forskolin and repSox	None	Injectable granular scaffold encapsulated with PLNA nanoparticles for the treatment of myocardial infarction	[101]
	Heparin nanoparticles	Human endothelial colony‐forming cells (hECFCs)	Heparin nanoparticle‐loaded microporous annealed particle scaffolds guide angiogenesis in vitro and modulate vascularization in vivo	[97b]
	Hydroxyapatite methacrylate nanoparticles	None	Injectable granular scaffold assembled from nanocomposite microgel building blocks for efficient bone regeneration	[87]
	Zinc‐doped hydroxyapatite nanoparticles	L929 fibroblasts; RAW264.7 macrophages	Granular hydrogels assembled in situ from Zn^2^⁺‐functionalized nanocomposite microgels for efficient chronic wound care	[102]
	mRNA‐lipid‐based nanoparticles	NIH/3T3 murine fibroblasts	MAP scaffold as a novel mRNA release matrix to produce desired therapeutic proteins	[86b]
	Polyethylenimine/DNA (PEI/DNA) nanoparticles	Human embryonic kidney 293 (HEK293); Chinese hamster ovary (CHO) cell line	Granular scaffolds for gene delivery enabled sustained production of active antibodies and viruses for 30 d	[95]
	PEI/DNA nanoparticles	Mouse mesenchymal stem cells (MSCs); human dermal fibroblasts (HDFs); primary neural progenitor cells (NPCs)	Injectable microporous granular hydrogel scaffolds combined with freeze‐dried nanoparticle formulations enable efficient and long‐term nucleic acid delivery	[103]

Genetic drugs represent another common therapeutic payload encapsulated in granular hydrogels. Given the hydrophilicity, genetic drugs can be loaded directly in the hydrogel or encapsulated within nanocarriers for controlled release. Compared with protein‐based cargos, genetic drugs offer advantages such as long‐term expression, high specificity, and gene editing capabilities. However, they still face limitations in terms of stability, effectiveness, cellular uptake efficiency, and targeted delivery to specific tissues.^[^ ^98^ ^]^ For granular systems, Kuang et al. directly embedded cytosine guanosine oligonucleotide (CpG ODN) and liquid nitrogen‐inactivated 4T1 breast cancer cells into GelMA microgels to promote the aggregation and activation of dendritic cells (CD11c+), macrophages (F4/80+), CD3+ T cells, B cells (CD19+), and natural killer cells (CD49b+).^[^ ^97d^ ^]^ This approach significantly enhanced the antitumor immune response efficiency of the granular hydrogel. Kurt et al. chose linear polyethyleneimine (PEI)/DNA nanoparticles with HA coating to establish a long‐acting gene delivery platform via MMP‐induced hydrogel degradation, yielding higher transfection expression levels and a duration upwards of one month in D1 MSCs and HDFs.^[^ ^95^ ^]^ In addition to protein and genetic drugs, small molecules within granular hydrogels could be delivered in a localized and controlled manner to minimize off‐target effects and drug toxicity. Hence, Wang et al. designed aminooxyacetic acid (AOA) as a PEG‐TK‐AOA prodrug and utilized the high ROS concentration in the myocardial infarction (MI) microenvironment to control its targeted release from granular scaffolds.^[^ ^70a^ ^]^ This strategy enhanced Treg differentiation efficiency and cell secretion (e.g., IL‐10 and IGF‐2), while reducing oxidative and inflammatory damage.

### 3.6 Cell Incorporation Strategy

To take advantage of the microporous properties of granular hydrogel scaffolds, the common cellular incorporation strategy is to seed cells on the surface and the interstitial spaces of microgels, which is also the main consideration in the above discussion. The specific procedure is straightforward, requiring only the mixing of the cell solution with the microgel either before or after annealing or the pipetting of cells onto the surface of the granular hydrogel scaffolds, followed by incubation.^[^ ^50^, ^90^ ^]^ This means that the limitation of biocompatibility and toxicity of microgel manufacturing and gelation process is reduced. Currently, this cellular incorporation strategy has been successfully applied to muscle repair, corneal tissue regeneration, cartilage repair, spinal cord injury recovery, heart function recovery, wound healing, and traumatic injury therapy.^[^ ^14^, ^27^, ^60^, ^70^, ^71^, ^75^, ^104^ ^]^ Although seeding cells on microgel surfaces offers many advantages, another widely used cellular incorporation strategy that should not be overlooked is encapsulating cells within microgels. In theory, cells encapsulated within microgel building blocks respond to the surrounding microenvironment in a similar way to those in bulk hydrogels, but the higher surface‐area‐to‐volume ratio of microgels will prevent low nutrient diffusion within the hydrogel core region, while the interstitial space between microgels offers a larger area for cellular expansion during culture.^[^ ^3b^ ^]^ Encapsulation strategy typically requires mixing cells with the gel precursor solution, followed by cell loading through subsequent microgel fabrication and annealing processes. This implies that mild microgel fabrication methods and cell‐friendly assembly strategies should be selected. After encapsulation, loaded cells are protected from shear force damage through the relative movement between microgels during the injection or printing of granular hydrogels, thereby improving their viability within the system post‐extrusion.^[^ ^12a^ ^]^ In support of this, Highley et al. demonstrated that NorHA microspheres loaded with NIH/3T3 fibroblasts could maintain the same cell viability (70%) as the initial encapsulation treatment after 3D printing onto the surface and secondary crosslinking.^[^ ^41a^ ^]^ Furthermore, a previous study has revealed that this cellular incorporation strategy also enables the cryopreservation of hMSCs, which allows long‐term storage and ready availability, and the thawed hMSCs exhibit the same functions as freshly processed stem cells.^[^ ^24a^ ^]^ Notably, this cell loading method also supports single‐cell compartmentalization, i.e., each microgel contains only one cell surrounded by a hydrogel matrix. This implies that granular hydrogel scaffolds can be utilized to investigate phenotypic heterogeneity within and between cell populations and to examine single‐cell behaviors such as proliferation, differentiation, and metabolism. In fact, earlier studies have shown that different encapsulation densities will affect the differentiation trend and secretion characteristics of MSCs.^[^ ^9^, ^105^ ^]^ However, the current mainstream single‐cell embedding method still relies on droplet microfluidics technology and requires precise control of the droplet volume to cell concentration ratio to ensure accurate encapsulation.^[^ ^106^ ^]^ The process typically follows a Poisson distribution, meaning that microgels have a 37% probability of containing a single cell, while most remain empty or contain multiple cells.^[^ ^107^ ^]^ In other words, subsequent sorting is required, and the yield is limited. For more details on single‐cell microgel encapsulation, readers are encouraged to refer to the progress report by Dubay et al.^[^ ^108^ ^]^ To date, granular hydrogel scaffolds loaded with cells by microgel encapsulation have been successfully applied to cartilage tissue regeneration, new blood vessel formation, and skin wound healing, among others.^[^ ^100^, ^109^ ^]^

## 4 Interplay of Granular Hydrogels and Cells: toward Artificial Tissues

In summary, granular hydrogels have shown great potential and flexibility for customizing complex cellular microenvironments in vitro. Based on the fast development and growing interests in this field, it is believed that eventually we can make artificial tissues with granular hydrogels and cells. From the literature reviewed in the previous sections, it is evident that the highly tunable granular hydrogel systems provide both physical and biochemical signals, similar to the microenvironment in natural tissues.^[^ ^110^ ^]^ However, the cooperative interplay of multiple factors (such as porosity, mechanical properties, degradability, and drug release) in the granular hydrogel is complex, as these factors are often interdependent. Furthermore, the live cells in the granular hydrogels actively shape the microenvironment, making it dynamically changeable over time. It is thus difficult to draw a simple conclusion about how to design the granular hydrogel systems toward artificial tissues. Nevertheless, we briefly delve into this topic in the following paragraphs and highlight some future directions in this field.

**Figure 5:**
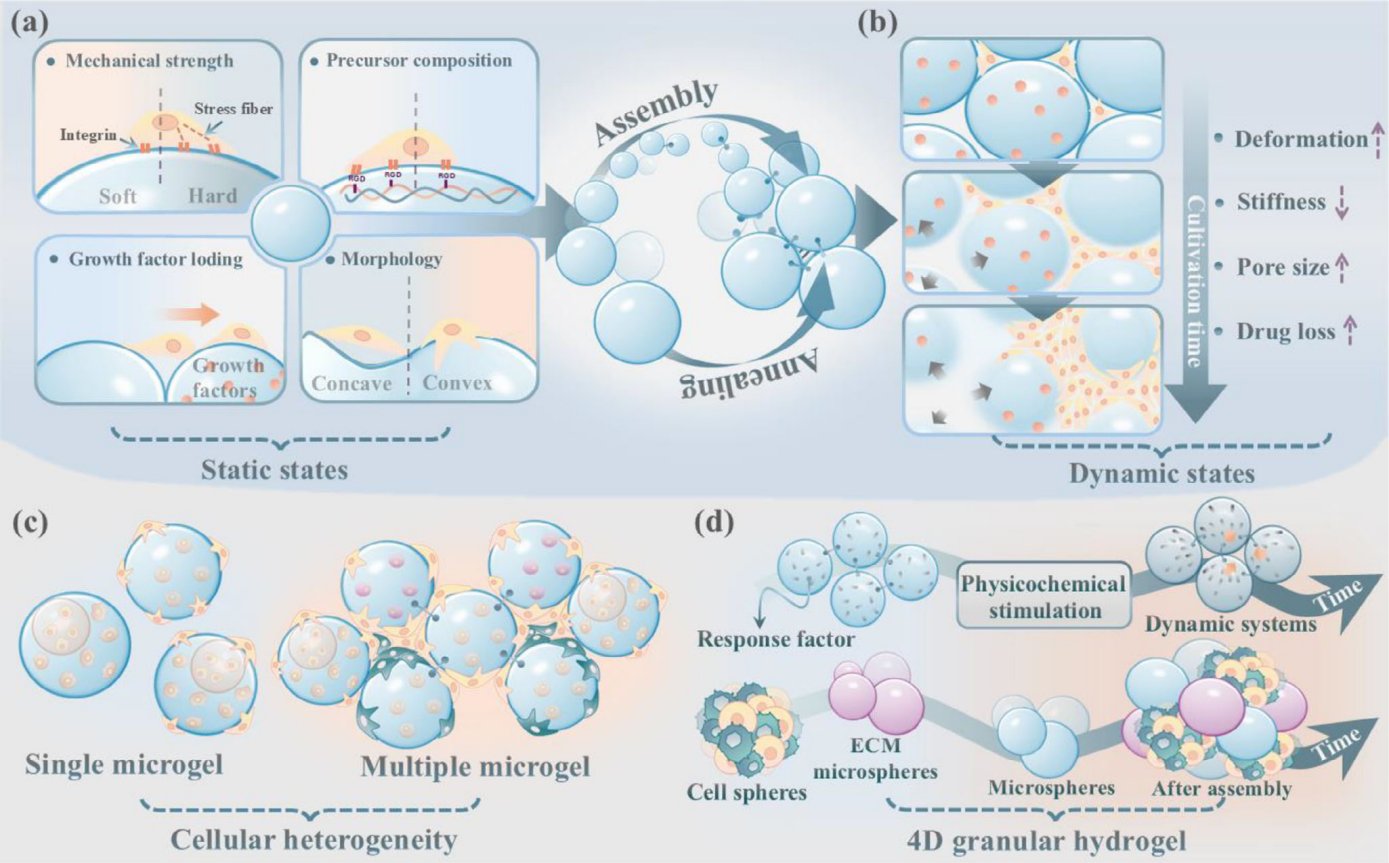
Visualization of granular hydrogel–cell interactions and future research perspectives. a) Schematic illustrating individual microgels providing biochemical cues, mechanical stimuli, and 2D topographical features essential for cell growth in static environments. b) Dynamic interactions between degrading microgels and resulting physicochemical changes such as stiffness reduction, curvature deformation, pore expansion, and drug release. Orange circles indicate encapsulated drugs; gray arrows represent microgel degradation from exterior to interior. c) Conceptual vision for designing single or multiple microgels to induce cellular heterogeneity in granular hydrogels. d) Conceptual design of 4D granular hydrogel biomaterials integrating cell spheroids, extracellular matrix (ECM) microspheres, and stimuli‐responsive microgels.

To simplify the interdependent factors in granular hydrogel design, we analyze the system by distinguishing between static and dynamic states (**Figure** **5**). The static state refers to the multifactor interplay within the granular scaffold without considering the time scale, whereas the dynamic state incorporates the time scale into the analysis.

Typically, in the static state, biochemical factors, mechanical stimulation, and 2D topographical features essential for cell growth can be achieved by designing the precursor composition, mechanical strength, size, shape, growth factor presentation, and functional modifications of individual microgels (Figure 5a). This aligns with the single‐factor regulation previously discussed in this review. However, when microgels are assembled or annealed, certain final properties of granular systems are inherently coupled with multiple intra‐/inter‐microgel factors. For example, without annealing, modifying microgel morphology or packing density inevitably alters both the porosity and mechanical strength of the overall granular hydrogel at the same time. This occurs due to changes in the contact area and the intervoid spaces between microgels. Introducing annealing appears to be an effective strategy to decouple porosity and mechanical strength of granular systems. Nevertheless, the annealing also restricts the ability to adjust microgel packing density and porosity. Furthermore, it is possible to regulate the mechanical properties of the entire system by modifying the stiffness of individual microgels. However, it is important to consider that microgel mechanical strength is inherently linked to its deformability and degradation rate, which further influence subsequent dynamic changes.

Regarding the dynamic state, as the granular hydrogel degrades during culturing, it inevitably leads to stiffness reduction, curvature deformation, pore expansion, and drug release from microgels (Figure 5b). These phenomena cannot be decoupled, meaning that cell behavior becomes even more unpredictable when the time dimension is considered. Therefore, the degradation behavior of the granular system must be carefully tailored to the research purpose. For instance, gradual degradation is essential for granular scaffolds to promote endogenous repair in vivo, facilitating rapid host cell infiltration and vascular network formation. When granular scaffolds are used to simulate extracellular matrices in vitro, degradation may introduce additional variables that may complicate analysis.

The heterogeneity of granular hydrogels adds another dimension of complexity but also enhances the ability of granular systems to mimic the natural cellular microenvironment. We anticipate that future work will focus on cellular heterogeneity by co‐culturing multiple cell types within granular hydrogels and on compartmentalized assembly of microgel units to better mimic tissue‐like complexity, eventually leading to native organ reconstruction in vitro (Figure 5c). Cellular heterogeneity can be analyzed from two perspectives: within a single microgel or across multiple microgels. For an individual microgel, the conventional co‐culture approach involves encapsulating one cell type within the microgel, while coating another on its outer surface (e.g., adMSCs and HUVECs).^[^ ^111^ ^]^ More recently, He et al. used aqueous two‐phase emulsion systems to prepare controllable multichamber hydrogel microcarriers to achieve a multicell co‐culture model within a single microgel (e.g., Caco‐2 and HepG2 cells).^[^ ^112^ ^]^ Beyond individual microgels, cellular heterogeneity can also be achieved at the multi‐microgel level. By strategically assembling microgels with predefined cellular cargos, granular hydrogel systems with heterogeneous cell distributions could be created. For example, Ribezzi et al. developed a novel embedded extrusion‐volumetric printing technology, enabling microgels loaded with various cells (e.g., insulin‐producing β‐cells and hMSCs) to achieve precise local arrangement and patterning at high cell density.^[^ ^113^ ^]^ They demonstrated that local high‐density, multicell type arrangements effectively simulate various tissue microenvironments (e.g., blood vessels, nerves, and pancreatic islet tissue). Based on these inspiring studies, we anticipate more future work will focus on creating cellular heterogeneity granular hydrogels mimicking disease models (e.g., diabetes, cancers) and regenerative medicine (e.g., soft tissue transplantation).

Besides cellular heterogeneity, by preprogramming microgels to respond to stimuli, such as light, heat, and chemical signals, granular hydrogels can theoretically undergo dynamic changes with precise spatiotemporal control after packing or assembly. This would enable future 4D biomaterials to mimic the dynamic processes of tissue development or even mimic the changes of cellular microenvironments during certain disease development (Figure 5d).^[^ ^114^ ^]^ The potential of 4D granular culture platforms can be improved by incorporating special dynamically changing building blocks, such as magnetic microgels, cell spheres, and ECM microspheres, enhancing the dynamic functionality, responsiveness, and biomimicry of the entire granular scaffold.^[^ ^53^, ^93^, ^115^ ^]^

Overall, granular hydrogels provide new solutions for in vitro cell culture and in vivo tissue engineering. Although the current granular hydrogel design still faces numerous challenges, with continuous material engineering and development, we are approaching unprecedented complexity and dynamic control, which will eventually lead to natural tissue mimics for versatile biomedical applications.

## 5 Literature Survey and Data Collection Methodology

The literature survey underlying the analysis presented herein was conducted using a systematic approach. Relevant publications were identified through comprehensive searches in Google Scholar, PubMed, Web of Science, and ScienceDirect databases. The search was performed using the following keywords: *microgels*, *microspheres*, *hydrogel particle*, *granular hydrogel*, *bottom‐up hydrogels*, *building blocks*, and *microporous annealed particles*. The search period spanned from January 2000 to June 2025. After excluding studies focused solely on bulk hydrogels or microgel synthesis, ≈200 articles were initially screened. From these, we manually excluded publications related to metal adsorption, water treatment, or two‐phase systems in which microgel particles were embedded within another hydrogel matrix. Ultimately, we identified over 100 articles specifically addressing granular hydrogel systems for applications in targeted delivery, tissue engineering, and ECM mimicry that aligned with the scope of this review.

Therefore, over 100 papers were carefully selected to extract and summarize granular hydrogel parameters, detailed in Table S1 (Supporting Information). These parameters include microgel composition, functional modifications, intra‐microgel crosslinking strategies, and inter‐microgel secondary crosslinking approaches. Available data regarding detailed intra‐ and inter‐microgel characteristics were further organized into Tables S2–S4 (Supporting Information). Specifically, Table S2 (Supporting Information) collects intra‐microgel attributes such as size, shape, and stiffness of individual microgels, whereas Tables S3 and S4 (Supporting Information) summarize collective properties of aggregated granular hydrogels, including porosity, pore area, and storage modulus.

Porosity values were either directly obtained from the main text of the selected studies or estimated from graphical data and tables. When only void volume fractions were reported, these were converted to percentage void space for consistency. Mechanical properties were obtained from values explicitly reported in the text or inferred from figures and tables. Due to variations in measurement techniques across studies, intra‐microgel stiffness was recorded as either Young's modulus or storage modulus, as specified in each case.

## Conflict of Interest

The authors declare no conflicts of interest.

## Author Contributions

S.F. conceptualized the idea, conducted the literature review, curated data from the selected studies, drafted the initial manuscript, and prepared all figures and tables. K.C. contributed to the generation and organization of tables. S.W. also contributed to the idea conceptualization, provided project funding and oversight, writing support, and critical revisions of the manuscript. All authors read and approved the final manuscript.

## Supporting information



Supporting Information

## References

[1] a) S. Caliari , J. Burdick , Nat. Methods 2016, 13, 405; 27123816 10.1038/nmeth.3839 PMC5800304

[2] a) R. Foudazi , R. Zowada , I. Manas‐Zloczower , D. Feke , Langmuir 2023, 39, 2092; 36719086 10.1021/acs.langmuir.2c02253

[3] a) O. Chaudhuri , J. Cooper‐White , P. Janmey , D. Mooney , V. Shenoy , Nature 2020, 584, 535; 32848221 10.1038/s41586-020-2612-2 PMC7676152

[4] A. Agrawal , Y. Javanmardi , S. A. Watson , B. Serwinski , B. Djordjevic , W. Li , A. R. Aref , R. W. Jenkins , E. Moeendarbary , npj Biol. Phys. Mech. 2025, 2, 3. 39917412 10.1038/s44341-024-00007-x PMC11794153

[5] a) S. Lien , L. Ko , T. Huang , Acta Biomater. 2009, 5, 670; 18951858 10.1016/j.actbio.2008.09.020

[6] N. Annabi , J. W. Nichol , X. Zhong , C. Ji , S. Koshy , A. Khademhosseini , F. Dehghani , Tissue Eng., Part B 2010, 16, 371. 10.1089/ten.teb.2009.0639 PMC2946907 20121414

[7] A. Leferink , D. Schipper , E. Arts , E. Vrij , N. Rivron , M. Karperien , K. Mittmann , C. van Blitterswijk , L. Moroni , R. Truckenmüller , Adv. Mater. 2014, 26, 2592. 24395427 10.1002/adma.201304539

[8] C. Bektas , Y. Mao , Gels 2023, 10, 28. 38247752 10.3390/gels10010028 PMC10815488

[9] A. Caldwell , B. Aguado , K. Anseth , Adv. Funct. Mater. 2020, 30, 1907670. 33841061 10.1002/adfm.201907670 PMC8026140

[10] A. Widener , A. Roberts , E. Phelps , Adv. Healthcare Mater. 2024, 13, 2303005. 10.1002/adhm.202303005 PMC11196388 38145369

[11] V. G. Muir , S. Weintraub , A. P. Dhand , H. Fallahi , L. Han , J. A. Burdick , Adv. Sci. 2023, 10, 2206117. 10.1002/advs.202206117 PMC10074081 36717272

[12] a) C. Tuftee , E. Alsberg , I. Ozbolat , M. Rizwan , Trends Biotechnol. 2024, 42, 339; 37852853 10.1016/j.tibtech.2023.09.007 PMC10939978

[13] T. Farjami , A. Madadlou , Food Hydrocolloids 2017, 62, 262.

[14] a) T. J. Tigner , G. Dampf , A. Tucker , Y.‐C. Huang , V. Jagrit , A. J. Clevenger , A. Mohapatra , S. A. Raghavan , J. N. Dulin , D. L. Alge , Adv. Healthcare Mater. 2024, 13, 2303912; 10.1002/adhm.202303912 PMC11390979 38470994

[15] S. Xin , D. Chimene , J. Garza , A. Gaharwar , D. Alge , Biomater. Sci. 2019, 7, 1179. 30656307 10.1039/c8bm01286e PMC9179007

[16] E. Sideris , D. Griffin , Y. Ding , S. Li , W. M. Weaver , D. D. Carlo , T. Hsiai , T. Segura , ACS Biomater. Sci. Eng. 2016, 2, 2034. 33440539 10.1021/acsbiomaterials.6b00444

[17] Y. Zhu , Y. Sun , B. Rui , J. Lin , J. Shen , H. Xiao , X. Liu , Y. Chai , J. Xu , Y. Yang , ACS Appl. Mater. Interfaces 2022, 14, 40674. 36052731 10.1021/acsami.2c11956

[18] a) C. Chang , H. Nguyen , E. Frahm , K. Kolaczyk , C. Lin , RSC Appl. Polym. 2024, 2, 656; 39035826 10.1039/d3lp00249g PMC11255916

[19] J. Lou , D. Mooney , Nat. Rev. Chem. 2022, 6, 726. 37117490 10.1038/s41570-022-00420-7

[20] O. Chaudhuri , Biomater. Sci. 2017, 5, 1480. 28584885 10.1039/c7bm00261k

[21] L. Smith , S. Cho , D. Discher , Physiology 2018, 33, 16. 29212889 10.1152/physiol.00026.2017 PMC5866410

[22] K. Vining , A. Stafford , D. Mooney , Biomaterials 2019, 188, 187. 30366219 10.1016/j.biomaterials.2018.10.013 PMC6279497

[23] a) J. de Rutte , J. Koh , D. Di Carlo , Adv. Funct. Mater. 2019, 29, 1900071;

[24] a) O. B. L. Jeon , Y. Hinton , T. Feinberg , A. E. Alsberg , Mater. Today Chem. 2019, 12, 61; 30778400 10.1016/j.mtchem.2018.11.009 PMC6377241

[25] O. Chaudhuri , L. Gu , D. Klumpers , M. Darnell , S. A. Bencherif , J. C. Weaver , N. Huebsch , H.‐P. Lee , E. Lippens , G. N. Duda , D. J. Mooney , Nat. Mater. 2016, 15, 326. 26618884 10.1038/nmat4489 PMC4767627

[26] J. Karam , B. Singer , H. Miwa , L. H. Chen , K. Maran , M. Hasani , S. Garza , B. Onyekwere , H. Yeh , S. Li , D. D. Carlo , S. K. Seidlits , Acta Biomater. 2023, 169, 228. 37572983 10.1016/j.actbio.2023.08.001 PMC11729822

[27] G. Tanner , L. Schiltz , N. Narra , M. Figueiredo , T. Qazi , Adv. Healthcare Mater. 2024, 13, 2303576. 10.1002/adhm.202303576 38329892

[28] S. Y. Recalde Phillips , K. D. Perez‐Ponce , E. Ruben , T. Baig , E. Poux , C. A. Gregory , D. L. Alge , Biomacromolecules 2024, 25, 5798. 39190621 10.1021/acs.biomac.4c00465 PMC11388458

[29] Y. Liu , A. Suarez‐Arnedo , E. Caston , L. Riley , M. Schneider , T. Segura , Adv. Mater. 2023, 35, 2304049. 10.1002/adma.202304049 PMC10874253 37721722

[30] D. Rommel , M. Mork , S. Vedaraman , C. Bastard , L. Guerzoni , Y. Kittel , R. Vinokur , N. Born , T. Haraszti , L. D. Laporte , Adv. Sci. 2022, 9, 2103554. 10.1002/advs.202103554 PMC8981485 35032119

[31] A. Suturin , A. Kruger , K. Neidig , N. Klos , N. Dolfen , M. Bund , T. Gronemann , R. Sebars , A. Manukanc , G. Yazdani , Y. Kittel , D. Rommel , T. Harasti , J. Köhler , Adv. Healthcare Mater. 2022, 11, 2200989. 10.1002/adhm.202200989 PMC11469137 36100464

[32] D. Stornello , J. Kim , Z. Chen , K. Heaton , T. Qazi , ACS Biomater. Sci. Eng. 2025, 11, 1242. 39788546 10.1021/acsbiomaterials.4c02102 PMC11817678

[33] R. Tang , L. Shang , P. Scumpia , D. Di Carlo , Adv. Healthcare Mater. 2024, 13, 2302477. 10.1002/adhm.202302477 PMC11102933 37985462

[34] A. Kedzierski , S. Kheirabadi , A. Jaberi , Z. Ataie , C. L. Mojazza , M. L. Williamson , A. M. Hjaltason , A. Risbud , Y. Xiang , A. Sheikhi , Adv. Funct. Mater. 2025, 35, 2417704.

[35] M. Mirbagheri , V. Adibnia , B. R. Hughes , S. D. Waldman , X. Banquy , D. K. Hwang , Mater. Horiz. 2019, 6, 45.

[36] K. Kilian , B. Bugarija , B. Lahn , M. Mrksich , Proc. Natl. Acad. Sci. USA 2010, 107, 4872. 20194780 10.1073/pnas.0903269107 PMC2841932

[37] M. Werner , S. Blanquer , S. Haimi , G. Korus , J. Dunlop , G. Duda , D. Grijpma , A. Petersen , Adv. Sci. 2017, 4, 1600347. 10.1002/advs.201600347 PMC5323878 28251054

[38] R. Assoian , N. Bade , C. Cameron , K. Stebe , Open Biol. 2019, 9, 190155. 31640476 10.1098/rsob.190155 PMC6833222

[39] Y. Park , Y. Choi , J. Lee , Biomater. Sci. 2025, 13, 1849. 40012335 10.1039/d4bm01659a

[40] L. Riley , L. Schirmer , T. Segura , Curr. Opin. Biotechnol. 2019, 60, 1. 30481603 10.1016/j.copbio.2018.11.001 PMC6534490

[41] a) C. Highley , K. Song , A. Daly , J. Burdick , Adv. Sci. 2019, 6, 1801076; 10.1002/advs.201801076 PMC6325587 30643716

[42] H.‐P. Lee , R. Davis Jr. , T.‐C. Wang , K. A. Deo , K. X. Cai , D. L. Alge , T. P. Lele , A. K. Gaharwar , ACS Appl. Bio Mater. 2023, 6, 3683. 10.1021/acsabm.3c00337 PMC10863386 37584641

[43] M. van Hecke , J. Phys.: Condens. Matter 2010, 22, 033101. 21386274 10.1088/0953-8984/22/3/033101

[44] V. Muir , T. Qazi , J. Shan , J. Groll , J. Burdick , ACS Biomater. Sci. Eng. 2021, 7, 4269. 33591726 10.1021/acsbiomaterials.0c01612 PMC8966052

[45] G. Britchfield , A. Daly , Biofabrication 2025, 17, 025004. 10.1088/1758-5090/adaa22 39808934

[46] H. Du , A. Cont , M. Steinacher , E. Amstad , Langmuir 2018, 34, 3459. 29489377 10.1021/acs.langmuir.7b04163

[47] T. Qazi , J. Wu , V. Muir , S. Weintraub , S. Gullbrand , D. Lee , D. Issadore , J. Burdick , Adv. Mater. 2022, 34, 2109194. 10.1002/adma.202109194 PMC8957565 34932833

[48] a) L. Ribeiro , V. Gaspar , R. Sobreiro‐Almeida , E. Camargo , J. Mano , Adv. Mater. Technol. 2023, 8, 202300209;

[49] S. Xin , J. Dai , C. Gregory , A. Han , D. Alge , Adv. Funct. Mater. 2020, 30, 1907102. 38213754 10.1002/adfm.201907102 PMC10783553

[50] A. Jaberi , A. Kedzierski , S. Kheirabadi , Y. Tagay , Z. Ataie , S. Zavari , M. Naghashnejad , O. Waldron , D. Adhikari , G. Lester , C. Gallagher , A. Borhan , D. Ravnic , E. Tabdanov , A. Sheikhi , Adv. Healthcare Mater. 2024, 13, 2402489. 10.1002/adhm.202402489 PMC11828485 39152936

[51] A. Anderson , E. Nicklow , T. Segura , Acta Biomater. 2022, 150, 111. 35917913 10.1016/j.actbio.2022.07.051 PMC10329855

[52] M. Hirsch , A. Charlet , E. Amstad , Adv. Funct. Mater. 2020, 31, 2005929.

[53] N. Caprio , M. Davidson , A. Daly , J. Burdick , Adv. Mater. 2024, 36, 2312226. 10.1002/adma.202312226 PMC10994732 38178647

[54] Y. Du , E. Lo , S. Ali , A. Khademhosseini , Proc. Natl. Acad. Sci. USA 2008, 105, 9522. 18599452 10.1073/pnas.0801866105 PMC2474514

[55] S. Chung , W. Park , S. Shin , S. A. Lee , S. Kwon , Nat. Mater. 2008, 7, 581. 18552850 10.1038/nmat2208

[56] S. Chung , Y. Jung , S. Kwon , Small 2011, 7, 796. 21322106 10.1002/smll.201001806

[57] S. Tasoglu , E. Diller , S. Guven , M. Sitti , U. Demirci , Nat. Commun. 2014, 5, 3124. 24469115 10.1038/ncomms4124 PMC3947548

[58] F. Xu , T. D. Finley , M. Turkaydin , Y. Sung , U. A. Gurkan , A. S. Yavuz , R. O. Guldiken , U. Demirci , Biomaterials 2011, 32, 7847. 21820734 10.1016/j.biomaterials.2011.07.010 PMC3244142

[59] N. Dinh , R. Luo , M. Christine , W. N. Lin , W. Shin , J. C. Goh , C. Chen , Small 2017, 13, 1700684. 10.1002/smll.201700684 28481437

[60] a) M. S. Asadikorayem , F. Weber , P. Weber , D. M. Zenobi‐Wong , Adv. Healthcare Mater. 2024, 13, 2301831; 10.1002/adhm.202401623 39007282

[61] a) Q. Feng , D. Li , Q. Li , X. Cao , H. Dong , Bioact. Mater. 2022, 9, 105; 34820559 10.1016/j.bioactmat.2021.07.020 PMC8586262

[62] D. Bruzewicz , A. McGuigan , G. Whitesides , Lab Chip 2008, 8, 663. 18432334 10.1039/b719806j

[63] a) E. Alsberg , E. Feinstein , M. Joy , M. Prentiss , D. Ingber , Tissue Eng. 2006, 12, 3247; 17518638 10.1089/ten.2006.12.3247

[64] F. Xu , C.‐A. M. Wu , V. Rengarajan , T. D. Finley , H. O. Keles , Y. Sung , B. Li , U. A. Gurkan , U. Demirci , Adv. Mater. 2011, 23, 4254. 21830240 10.1002/adma.201101962 PMC3534971

[65] a) R. Tognato , R. Parolini , S. Jahangir , J. Ma , S. Florczak , R. G. Richards , R. Levato , M. Alini , T. Serra , Mater. Today Bio 2023, 22, 100775; 10.1016/j.mtbio.2023.100775 PMC10477805 37674778

[66] Y. Du , M. Ghodousi , E. Lo , M. Vidula , O. Emiroglu , A. Khademhosseini , Biotechnol. Bioeng. 2009, 105, 655. 10.1002/bit.22552 PMC2833321 19777588

[67] C. M. Dumont , M. A. Carlson , M. K. Munsell , A. J. Ciciriello , K. Strnadova , J. Park , B. J. Cummings , A. J. Anderson , L. D. Shea , Acta Biomater. 2019, 86, 312. 30610918 10.1016/j.actbio.2018.12.052 PMC6369008

[68] a) A. Milani , A. Freemont , J. Hoyland , D. Adlam , B. Saunders , Biomacromolecules 2012, 13, 2793; 22877136 10.1021/bm3007727

[69] a) A. Caldwell , G. Campbell , K. Shekiro , K. Anseth , Adv. Healthcare Mater. 2017, 6, 1700254; 10.1002/adhm.201700254 PMC5550331 28485127

[70] a) S. Wang , K. Wang , W. Cao , L. Song , S. Li , Z. Zhai , L. Shen , Y. Zhu , W. Liu , C. Gao , Chem. Eng. J. 2024, 497, 154933;

[71] A. J. Feliciano , Y. A. Selsouli , P. Habibovic , Z. N. T. Birgani , L. Moroni , M. B. Baker , Biomater. Sci. 2024, 12, 4993. 39169887 10.1039/d4bm00409d

[72] S. K. Nair , S. Basu , B. Sen , M.‐H. Lin , A. N. Kumar , Y. Yuan , P. J. Cullen , D. Sarkar , Sci. Rep. 2019, 9, 1072. 30705322 10.1038/s41598-018-37788-w PMC6355882

[73] H. Qi , M. Ghodousi , Y. Du , C. Grun , H. Bae , P. Yin , A. Khademhosseini , Nat. Commun. 2013, 4, 2275. 24013352 10.1038/ncomms3275 PMC3768014

[74] R. Merindol , S. Loescher , A. Samanta , A. Walther , Nat. Nanotechnol. 2018, 13, 730. 29941888 10.1038/s41565-018-0168-1 PMC6082344

[75] a) M. T. Tedesco , D. Di Lisa , P. Massobrio , N. Colistra , M. Pesce , T. Catelani , E. Dellacasa , R. Raiteri , S. Martinoia , L. Pastorino , Biomaterials 2018, 156, 159; 29197747 10.1016/j.biomaterials.2017.11.043

[76] S. Bulut , D. Gunther , M. Bund , C. Haats , T. Bissing , C. Bastard , M. Wessling , L. D. Laporte , A. Pich , Adv. Healthcare Mater. 2024, 13, 2302957. 10.1002/adhm.202302957 37988182

[77] A. D'Elia , O. Jones , G. Canziani , B. Sarkar , I. Chaiken , C. Rodell , ACS Biomater. Sci. Eng. 2024, 10, 1577. 38357739 10.1021/acsbiomaterials.3c01906 PMC10934254

[78] N. F. Truong , E. Kurt , N. Tahmizyan , S. C. Lesher‐Pérez , M. Chen , N. J. Darling , W. Xi , T. Segura , Acta Biomater. 2019, 94, 160. 31154058 10.1016/j.actbio.2019.02.054 PMC7444265

[79] a) L. A. Krattiger , D. B. Emiroglu , S. Pravato , L. O. Moser , O. A. Bachmann , S. Y. La Cioppa , G. J. R. Rivera , J. A. Burdick , A. J. deMello , M. W. Tibbitt , M. Ehrbar , Adv. Funct. Mater. 2024, 34, 2310507;

[80] M. Asadikorayem , L. Brunel , P. Weber , S. Heilshorn , M. Zenobi‐Wong , Biomater. Sci. 2024, 12, 5504. 39347711 10.1039/d4bm00233d PMC11441418

[81] A. Seymour , D. Kilian , R. Navarro , S. Hull , S. Heilshorn , Biomater. Sci. 2023, 11, 7598. 37824082 10.1039/d3bm00721a PMC10842430

[82] Y. Liu , A. Suarez‐Arnedo , L. Riley , T. Miley , J. Xia , T. Segura , Adv. Healthcare Mater. 2023, 12, 2300823. 10.1002/adhm.202300823 PMC10592513 37165945

[83] A. Puiggali‐Jou , M. Asadikorayem , K. Maniura‐Weber , M. Zenobi‐Wong , Acta Biomater. 2023, 166, 69. 37030622 10.1016/j.actbio.2023.03.045

[84] a) N. Hen , E. Josef , M. Davidovich‐Pinhas , S. Levenberg , H. Bianco‐Peled , ACS Biomater. Sci. Eng. 2024, 10, 6734; 39344029 10.1021/acsbiomaterials.4c01136

[85] A. Charlet , F. Bono , E. Amstad , Chem. Sci. 2022, 13, 3082. 35414870 10.1039/d1sc06231j PMC8926196

[86] a) N. Darling , W. Xi , E. Sideris , A. Anderson , C. Pong , S. T. Carmichael , T. Segura , Adv. Healthcare Mater. 2020, 9, 1901391; 10.1002/adhm.201901391 PMC7340246 32329234

[87] T. Song , F. Zhao , L. Yan , P. Liu , J. Yang , C. Ruan , D. Li , Y. Xiao , X. Zhang , Biomaterials 2024, 309, 122601. 38713973 10.1016/j.biomaterials.2024.122601

[88] B. Pfaff , L. Pruett , N. Cornell , J. D. Rutte , D. D. Carlo , C. Highley , D. Griffin , ACS Biomater. Sci. Eng. 2021, 7, 422. 33423459 10.1021/acsbiomaterials.0c01580 PMC8408836

[89] B. B. Mendes , A. C. Daly , R. L. Reis , R. M. A. Domingues , M. E. Gomes , J. A. Burdick , Acta Biomater. 2021, 119, 101. 33130309 10.1016/j.actbio.2020.10.040

[90] T. Qazi , V. Muir , J. Burdick , ACS Biomater. Sci. Eng. 2022, 8, 1427. 35330993 10.1021/acsbiomaterials.1c01440 PMC10994272

[91] M. M. Coronel , K. E. Martin , M. D. Hunckler , P. Kalelkar , R. M. Shah , A. J. García , Small 2022, 18, 2106896. 10.1002/smll.202106896 PMC10288386 35274457

[92] S. Xin , C. A. Gregory , D. L. Alge , Acta Biomater. 2020, 101, 227. 31711899 10.1016/j.actbio.2019.11.009 PMC6960331

[93] N. Di Caprio , A. Hughes , J. Burdick , Sci. Adv. 2025, 11, adq5011. 10.1126/sciadv.adq5011 PMC11740954 39823334

[94] L. Pruett , A. Taing , N. Singh , S. Peirce , D. Griffin , Acta Biomater. 2022, 148, 171. 35660016 10.1016/j.actbio.2022.05.049 PMC10297728

[95] E. Kurt , G. Devlin , A. Asokan , T. Segura , Small 2024, 20, 2309911. 10.1002/smll.202309911 PMC11294003 38462954

[96] a) K. Wilson , S. Perez , M. Naffaa , S. Kelly , T. Segura , Adv. Mater. 2022, 34, 2201921; 10.1002/adma.202201921 PMC9645378 35731241

[97] a) C.‐C. Hsu , J. H. George , S. Waller , C. Besnard , D. A. Nagel , E. J. Hill , M. D. Coleman , A. M. Korsunsky , Z. Cui , H. Ye , Bioact. Mater. 2022, 9, 358; 34820576 10.1016/j.bioactmat.2021.07.008 PMC8586009

[98] X. Bian , L. Zhou , Z. Luo , G. Liu , Z. Hang , H. Li , F. Li , Y. Wen , ACS Nano 2025, 19, 4039. 39834294 10.1021/acsnano.4c11858

[99] D. P. Mayer , M. E. Nelson , D. Andriyanova , R. B. Filler , A. Ökten , O. Q. Antao , J. S. Chen , P. O. Scumpia , W. M. Weaver , C. B. Wilen , S. Deshayes , J. S. Weinstein , J. Controlled Release 2024, 370, 570. 10.1016/j.jconrel.2024.05.008 PMC11665867 38734312

[100] J. Chen , Q. Li , H. Li , C. Lv , H. Yu , Q. Feng , H. Dong , Bioact. Mater. 2025, 44, 220. 39497706 10.1016/j.bioactmat.2024.10.013 PMC11533518

[101] J. Fang , J. Koh , Q. Fang , H. Qiu , M. Archang , M. M. Hasani‐Sadrabadi , H. Miwa , X. Zhong , R. Sievers , D. Gao , R. Lee , D. D. Carlo , S. Li , Adv. Funct. Mater. 2020, 30, 2004307. 33708028 10.1002/adfm.202004307 PMC7942842

[102] Z. Yuan , Z. Wan , Z. Tian , Y. Han , X. Huang , Y. Feng , W. Xie , X. Duan , S. Huang , X. Liu , J. Huang , Chem. Eng. J. 2022, 450, 138076.

[103] E. Kurt , T. Segura , Adv. Healthcare Mater. 2022, 11, 2101867. 10.1002/adhm.202101867 PMC8810690 34742164

[104] a) F. A. Surman , M. Weber , P. Weber , D. M. Zenobi‐Wong , Biofabrication 2024, 16, 025004; 10.1088/1758-5090/ad1b1f 38176081

[105] A. S. Mao , J.‐W. Shin , S. Utech , H. Wang , O. Uzun , W. Li , M. Cooper , Y. Hu , L. Zhang , D. A. Weitz , D. J. Mooney , Nat. Mater. 2017, 16, 236. 27798621 10.1038/nmat4781 PMC5372217

[106] B. Tiemeijer , J. Tel , Front. Bioeng. Biotechnol. 2022, 10, 891461. 35782502 10.3389/fbioe.2022.891461 PMC9247248

[107] T. Kamperman , M. Karperien , S. Le Gac , J. Leijten , Trends Biotechnol. 2018, 36, 850. 29656795 10.1016/j.tibtech.2018.03.001

[108] R. Dubay , J. Urban , E. Darling , Adv. Funct. Mater. 2021, 31, 2009946. 36329867 10.1002/adfm.202009946 PMC9629779

[109] a) K. Flegeau , A. Puiggali‐Jou , M. Zenobi‐Wong , Biofabrication 2022, 14, 034105; 10.1088/1758-5090/ac6b58 35483326

[110] S. Cosson , E. Otte , H. Hezaveh , J. Cooper‐White , Stem Cells Transl. Med. 2015, 4, 156. 25575526 10.5966/sctm.2014-0203 PMC4303362

[111] R. Mahou , A. Vlahos , A. Shulman , M. Sefton , ACS Biomater. Sci. Eng. 2018, 4, 3704. 33429609 10.1021/acsbiomaterials.7b00356

[112] H. He , M. Hong , F. Yang , G. Wang , Y. Wang , M. Yang , D. Huang , H. Liu , Y. Wang , Biomacromolecules 2024, 25, 4469. 38877974 10.1021/acs.biomac.4c00516

[113] D. Ribezzi , M. Gueye , S. Florczak , F. Dusi , D. D. Vos , F. Manente , A. Hierholzer , M. Fussenegger , M. Caiazzo , T. Blunk , J. Malda , R. Levato , Adv. Mater. 2023, 35, 2301673. 10.1002/adma.202301673 37269532

[114] a) M. Sheikhi , S. Vakili , N. Karimi , F. Rafiemanzelat , A. Maleki , A. Taheri , Z. Mohamadnia , A. Ramazani , ACS Appl. Polym. Mater 2025, 7, 1717;

[115] R. Shaik , J. Brown , J. Xu , R. Lamichhane , Y. Wang , Y. Hong , G. Zhang , ACS Appl. Mater. Interfaces 2024, 16, 58346. 39413287 10.1021/acsami.4c12871 PMC11542188

