# Supplementary material for: Practical Guide to the Design of Granular Hydrogels for Customizing Complex Cellular Microenvironments
Source: Adv Healthc Mater. 2025 Jul 29;14(27):e01947. doi: 10.1002/adhm.202501947 (PMC12538541; doi:10.1002/adhm.202501947)
Supplement: Supplementary file 1 — Supporting Information [file ADHM-14-0-s001.docx]

**Supporting Information for “A Practical Guide to the Design of Granular Hydrogels for Customizing Complex Cellular Microenvironments”**

Shuhan Feng^1^, Kaiyang Chen^1^, Shiqi Wang^1, 2^ *

1. Drug Research Program, Division of Pharmaceutical Chemistry and Technology, Faculty of Pharmacy, University of Helsinki, Helsinki, Finland
2. Institute of Biotechnology, Helsinki Institute of Life Science, University of Helsinki, Finland

* E-mail: [shiqi.wang@helsinki.fi](mailto:shiqi.wang@helsinki.fi)

**Table S1** Microgel composition, functional modification, and crosslinking strategies of various assemble granular hydrogels.

| Composition of microgel | Chemical modification | Other functional modification | Internal crosslinking condition | Internal crosslinking strategies | Secondary Crosslinker | Secondary crosslinking strategies | Ref. |
| --- | --- | --- | --- | --- | --- | --- | --- |
| Poly(ethylene glycol) (PEG) | 8-arm PEG vinyl sulfone (8-arm PEG-VS) | RGD, Q-peptide and K-peptide | Dithiol MMP-sensitive peptide | Thiol-ene click reaction | Factor XIIIa | Enzymatic catalysis | ^[1]^ |
|  |  | RGD, Q-peptide and K-peptide | Dithiothreitol (DTT), Dithiol MMP-sensitive peptide | Thiol-ene click reaction | Factor XIIIa | Enzymatic catalysis | ^[2]^ |
|  |  | RGD | Poly(ethylene glycol) dithiol (PEG-DT) | Thiol-ene click reaction | VA-086+UV | Thiol-ene click reaction | ^[3]^ |
|  |  | RGD | PEG-DT | Thiol-ene click reaction | VA-086+UV | Thiol-ene click reaction | ^[4]^ |
|  | 8-arm PEG-VS and 8-arm PEG azide | Laminin Mouse Protein | 8-arm PEG-amine, plasmin-sensitive peptide | Vinylsulfone-amine click reaction; Azide/alkyne click reaction | None | Vinylsulfone-amine click reaction; Azide/alkyne click reaction | ^[5]^ |
|  | 8-arm PEG-mal | None | Plasmin-Sensitive YKND Crosslinking Peptide | Thiol-ene click reaction | Irgacure 2959+UV | Free radical polymerization | ^[6]^ |
|  | 8-arm PEG-norbornene (8-arm PEG-Norb) | CGRGDS peptide | PEG-DT | Thiol-ene click reaction | LAP +UV | Thiol-ene click reaction | ^[7]^ |
|  |  | CRGDS | LAP+DTT+UV | Free radical polymerization | None | Microfluidics-driven jamming | ^[8]^ |
|  |  | IL-6 aptamer  Heparin | LAP+DTT+UV | Free radical polymerization | None | Centrifugation-drive jamming | ^[9]^ |
|  |  | None | DTT+LAP+UV | Thiol-ene click reaction | None | Centrifugation-drive jamming | ^[10]^ |
|  | 8-arm PEG-Norb, PEG-DT | CGGGRGDS, CGGGRGDSK-FITC | α-cyclodextrin+LAP+UV | Thiol-ene click reaction | Heat treatment (50°C, 15 min) | Host-guest interactions | ^[11]^ |
|  | 4-arm PEG-VS | RGD, Q-peptide and K-peptide | Dithiol MMP-sensitive peptide | Thiol-ene click reaction | Factor XIIIa | Enzymatic catalysis | ^[12]^ |
|  |  | RGD, Q-peptide and K-peptide | Dithiol MMP-sensitive peptide, PEG-DT | Thiol-ene click reaction | Factor XIIIa | Enzymatic catalysis | ^[13]^ |
|  |  | RGD, Q-peptide and K-peptide | Dithiol MMP-sensitive peptide | Thiol-ene click reaction | PEG-DT | Thiol-ene click reaction | ^[14]^ |
|  |  | RGD, Q-peptide and K-peptide | Dithiol MMP-sensitive peptide, PEG-DT | Thiol-ene click reaction | PEG-DT | Thiol-ene click reaction | ^[15]^ |
|  | 4-arm PEG azide | RGD-azide | 8-arm-PEG- Dibenzocyclooctyne | Azide/alkyne click reaction | None | Azide/alkyne click reaction | ^[16]^ |
|  | 4-arm PEG-maleimide (4-arm PEG-mal) | RGD | 4-arm PEG-thiol (4-arm PEG-SH) | Thiol-ene click reaction | Eosin-Y+UV | Free radical polymerization | ^[17]^ |
|  |  | β-cyclodextrin-PEG, Adamantane-PEG | 4-arm PEG-SH | Thiol-ene click reaction; Guest-host reaction | None | Host-guest interactions | ^[18]^ |
|  |  | RGD | 4-arm PEG-SH, Dithiol MMP-sensitive peptide | Thiol-ene click reaction | Eosin-Y+UV | Free radical polymerization | ^[19]^ |
|  |  | RGD | Plasmin-Sensitive YKND Crosslinking Peptide | Thiol-ene click reaction | Irgacure 2959+UV | Free radical polymerization | ^[20]^ |
|  |  | RGD, Thiolated heparin | CGPQGIAGQDGCG | Thiol-ene click reaction | LAP+UV | Free radical polymerization | ^[21]^ |
|  |  | β-cyclodextrin-PEG-mal, Adamantane-PEG-mal | 4-arm PEG-SH | Thiol-ene click reaction | None | Host-guest interactions | ^[22]^ |
|  |  | RGD | Dithiol MMP-sensitive peptide, LAP+UV | Thiol-ene click reaction | LAP or Eosin Y+UV | Free radical polymerization | ^[23]^ |
|  | 4-arm PEG-mal, 4-arm PEG-VS, Methacrylamide 4-arm PEG macromer | RGD | 4-arm PEG-VS | Thiol-ene click reaction | LAP or Eosin Y+UV | Free radical polymerization | ^[24]^ |
|  | 4-arm PEG-acrylate | RGD | 4-arm PEG-SH | Thiol-ene click reaction | None | Centrifugation-drive jamming | ^[25]^ |
|  | 4-arm PEG-amide-norbornene | CGRGDS | KCGPQGIAGQCK, LAP+UV | Thiol-ene click reaction | PEG-di-tetrazine | iEDDA reaction | ^[26]^ |
|  | 4-arm PEG-amide-tetra-norbornene,  PEG-DT, PEG-ditetrazine | CGRGDS | KCGPQGIWGQCK, LAP+UV | Thiol-norb click reaction;  iEDDA reaction | LAP +UV or None | Free radical polymerization  iEDDA reaction | ^[27]^ |
|  | PEG-norbornene, PEG-DT | RGDS, α5β1 integrin-specific peptide | KCGPQGIWGQCK, CGPQGPAGQGCR | Thiol-ene click reaction | LAP +UV | Thiol-ene click reaction | ^[28]^ |
|  | PEG-diacrylate (PEG-DA) | DNA | I2959+UV | Free radical polymerization | None | physically driven self-assembly | ^[29]^ |
|  |  | None | I2959+UV | Free radical polymerization | I2959+UV | Free radical polymerization | ^[30]^ |
|  |  | Streptavidin; RGDS | I2959+UV | Free radical polymerization | None | physically driven self-assembly | ^[31]^ |
|  | PEG-tetra-norbornene, PEG-di-tetrazine | CGRGDS | KCGPQGIWGQCK, thiol-norbornene, LAP+UV | Thiol-ene click reaction | None | iEDDA reaction | ^[32]^ |
| Poly(N-vinylcaprolactam) | None | None | N,N′-Methylene bisacrylamide, 70°C | Free radical polymerization | Tannic acid | physically driven self-assembly | ^[33]^ |
| Acrylamide | 6-acrylamido-β- cyclodextrin, 6-Acrylamido-α-cyclodextrin, N-(1-Adamantyl)-acrylamide | None | N, N'-methylenebisacrylamide (MBA) | Free radical polymerization | None | Host-guest interactions | ^[34]^ |
| 2-acrylamido-2-methylpropane sulfonic acid (AMPS) | None | None | MBA, PI+UV | Free radical polymerization | PI+UV | Vacuum drive jamming | ^[35]^ |
| Poly(methyl methacrylate/methacrylic acid/ethylene glycol dimethacrylate) | Gglycidyl methacrylated poly(MMA/MAA/EGD) | None | APS | Free radical polymerization | APS+NaOH+TEMED | Free radical polymerization | ^[36]^ |
| Hyaluronic acid (HA) | None | None | 1,4-butanediol diglycidyl ether | Nucleophilic ring-opening addition reaction | None | Centrifugation-drive jamming | ^[37]^ |
|  | Thiolated hyaluronic acid (HA-SH) | RGD | PEG-VS | Thiol-ene click reaction | None | Thiol-ene click reaction | ^[38]^ |
|  | Methacrylated hyaluronic acid (HAMA) | RGD, Q-peptide and K-peptide | Dithiol MMP-sensitive peptide | Thiol-ene click reaction | Factor XIIIa | Enzymatic catalysis | ^[39]^ |
|  | Sulfated HAMA | None | LAP+UV | Free radical polymerization | Factor XIIIa | Enzymatic catalysis | ^[40]^ |
|  | Acrylated HA (HA-Ac) | RGD, Q-peptide and K-peptide | Dithiol MMP-sensitive peptide | Thiol-ene click reaction | Factor XIIIa | Enzymatic catalysis | ^[41]^ |
|  | Norbornene-hyaluronic acid (HA-Norb) | None | DL-Dithiothreitol, LAP+ UV | Thiol-ene click reaction | None | Vacuum drive jamming | ^[42]^ |
|  | HA-Norb | None | DTT, UV+LAP | Thiol-ene click reaction | None | Vacuum drive jamming | ^[43]^ |
|  | HA-Norb | None | DTT, UV+LAP | Thiol-ene click reaction | None | Centrifugation-drive jamming | ^[44]^ |
|  | HA-Norb | GCGYGRGDSPG | DTT, UV+LAP | Thiol-ene click reaction | None | Centrifugation-drive jamming | ^[45]^ |
|  | HA-Norb | GCGYGRGDSPG | DTT, I2959+UV | Thiol-ene click reaction | None | Centrifugation-drive jamming | ^[46]^ |
|  | HA-Norb | RGD | DTT, LAP+UV | Thiol-ene click reaction | UV | Thiol-ene click reaction | ^[47]^ |
|  | HA-Norb | - | DTT, Dithiol-pNIPAM, LAP +UV | Thiol-ene click reaction | tetra-PEG-thiol; LAP +UV | Thiol-ene click reaction | ^[48]^ |
|  | HA-Norb, mesenchymal stromal cell spheroids | None | DTT, I2959+UV | Thiol-ene click reaction | None | Cell-particle adhesion | ^[49]^ |
|  | HA-Norb, NA- cyclodextrin | None | DTT, Dithiol MMP-sensitive peptide, UV | Thiol-ene click reaction | UV | Host-guest interactions | ^[50]^ |
|  | HA-Norb, NA- Tetrazine | RGD | Dithiol MMP-sensitive peptide, LAP+UV | Thiol-ene click reaction | None | iEDDA reaction | ^[51]^ |
|  | HA-Norb, HA-pentenoates, HAMA | None | DTT, I2959+UV | Thiol-ene click reaction | DTT | Thiol-ene click reaction | ^[52]^ |
|  | Hydrazide-HA-Norb, Aldehyde- HA-Norb | None | DTT, I2959+UV | Thiol-ene click reaction | None | Hydrazide-aldehyde condensation Reaction | ^[53]^ |
| Collagen | None | None | None | Heat-induced physical crosslinking | NIH 3T3 cells, HepG2 cells, HUVECs, *etc.* | Cell-particle adhesion | ^[54]^ |
|  | None | None | EDC/NHS | Amide coupling reaction | Tannic acid, hydroxyapatite nanoparticles | physically driven self-assembly | ^[55]^ |
|  | None | None | EDC/NHS | Amide coupling reaction | Tannic acid | physically driven self-assembly | ^[56]^ |
| Gelatin | Gelatin B | None | None | Heat-induced physical crosslinking | Glyceraldehyde | Schiff base reaction | ^[57]^ |
|  | Gelatin A, gelatin B | None | Glutaraldehyde | Schiff base reaction | None | Electrostatic interactions | ^[58]^ |
|  | Methacrylated Gelatin (GelMA) | None | LAP+UV | Free radical polymerization | LAP+UV | Free radical polymerization | ^[59]^ |
|  | GelMA | Silicate Nanoparticles | LAP+UV | Free radical polymerization | None | Electrostatic interactions | ^[60]^ |
|  | GelMA | Lipofectamine MessengerMAX | I2959+UV | Free radical polymerization | I2959+UV | Free radical polymerization | ^[61]^ |
|  | GelMA | None | None | Heat-induced physical crosslinking | LAP+UV | Free radical polymerization | ^[62]^ |
|  | GelMA | None | 4 °C | Heat-induced physical crosslinking | Irgacure 2959 + UV | Free radical polymerization | ^[63]^ |
|  | GelMA | None | LAP+UV | Free radical polymerization | LAP+UV | Free radical polymerization | ^[64]^ |
|  | Gelatin-norbornene-carbohydrazide | None | 4-arm PEG-SH+LAP+UV, Oxidized dextran | Thiol-ene click reaction; iEDDA reaction; aldehyde-bearing reaction | 4-arm PEG- tetrazine | iEDDA reaction | ^[65]^ |
| Chitosan | O-Carboxymethyl Chitosan | None | Glutaraldehyde | Schiff base reaction | None | Electrostatic interactions | ^[66]^ |
|  | None | None | Ethanol+NaOH | Physical crosslinking | Neurons | Cell-particle adhesion | ^[67]^ |
| Alginate | Methacrylated alginate (AlgMA) | None | Ca^2+^ | Ionic crosslinking | LAP +UV | Free radical polymerization | ^[68]^ |
|  | Tetrazine-alginate,  Trans-cyclooctene-algiante, Norbornene-alginate | PEG3-Biotin | Ca^2+^ | Ionic crosslinking | None | iEDDA reaction | ^[69]^ |
|  | AlgMA | Carboxybetaine acrylamide; sulfobetaine methacrylate | Ca^2+^, LAP+UV | Ionic crosslinking; Free-radical polymerization | LAP+UV | Electrostatic interactions; Free-radical polymerization | ^[70]^ |
| Dextran | Hydroxyethyl methacrylate dextran (dex-HEMA) | Methacrylic acid (MAA), N, N-dimethyl aminoethyl methacrylate (DMAEMA) | HEMA groups | Free radical polymerization | None | Electrostatic interactions | ^[71]^ |
|  | Methacrylate dextran (DexMA) | RGD | LAP+UV | Free radical polymerization | Normal human dermal fibroblasts (NHDFs) | Cell-particle adhesion | ^[72]^ |
| Agarose | None | None | Heating (85°C) + Cooling (22°C) | Physical crosslinking | None | Centrifugation-drive jamming | ^[73]^ |
| κ-Carrageenan | None | None | K⁺ | Physical crosslinking | None | Centrifugation-drive jamming | ^[74]^ |
| Linear ssDNA | Adenine-rich sequences (A20-i), self-complementary sequences (p(A20-i-XL) | None | Heating+Cooling | Thermally induced phase separation; ssDNA base hybridization | Heating and Cooling | Thermally induced phase separation; ssDNA base hybridization | ^[75]^ |
| Decellularized extracellular matrix | None | None | pH=9, EDC/NHS | Physical crosslinking;  Amidation Reaction | HA-catechol | Schiff Base Reaction | ^[76]^ |
| Cartilage acellular matrix | None | None | EDC/NHS, Oxidized sodium alginate | Schiff Base Reaction; Amidation Reaction | None | Schiff Base Reaction | ^[77]^ |
| Chondroitin Sulfate (CS) | Aldehyde-methacrylated-CS, Hydrazide-methacrylated-CS | None | I2959+UV | Free radical polymerization | None | Hydrazide-aldehyde condensation Reaction | ^[78]^ |
| PEG and methacrylates | PEG-DA,  2-Aminoethyl methacrylate (AEMA) | GRGDSPC | LAP+UV | Free radical polymerization | 6-armed PEG-epoxy | Nucleophilic substitution reaction | ^[79]^ |
| PEG and Gelatin | Gelatin-norbornene, | None | 4-arm PEG-SH, LAP+UV | Free radical polymerization | LAP+UV | Thiol-ene click reaction | ^[80]^ |
|  | GelMA, PEG- cyclodextrin, PEG- Adamantane | None | LAP+UV | Free radical polymerization; | None | Host-guest interactions | ^[81]^ |
| HA and PEG | HA-Norb, 4arm-PEG- tetrazine | RGD | Dithiol MMP-sensitive peptide, LAP+UV | Thiol-ene click reaction | None | iEDDA reaction | ^[82]^ |
|  | HA-Norb, 4arm-PEG- tetrazine | RGD | DDT+LAP+UV | Thiol-ene click reaction | None | iEDDA reaction | ^[83]^ |
| HA and alginate | MeHA, 3-aminophenylboronic acid modified sodium alginate (SABA) | None | UV; OH⁻ | Free radical polymerization  Boron-oxygen dynamic crosslinking | OH⁻ | Boron-oxygen dynamic crosslinking | ^[84]^ |
| HA and gelatin | Methyl Furan Modified HA (MFHA), GelMA | None | Dual Maleimide Endcapped PEG (Mal-PEG-Mal), I2959+UV | Diels-Alder reaction | I2959+UV | Diels-Alder reaction | ^[85]^ |
|  | Thiolated gelatin (Gel-SH); vinyl sulfonated hyaluronic acid (HA-VS) | None | None | Thiol-Michael click reaction | Bone Mesenchymal Stem Cells | Cell-particle adhesion | ^[86]^ |
| HA and Chitosan | HAMA; Chitosan methacrylate (CHIMA) | None | LAP +UV | Free radical polymerization; Electrostatic interactions | None | Centrifugation drive jamming | ^[87]^ |
| HA and type I collagen | HA-Norb | None | Divinyl sulfone | Thiol-ene click reaction | None | Centrifugation drive jamming | ^[88]^ |
| HA and poly(β-hydrazide esters) (HB- PBHEs) | HA-SH | RGD | None | Thiol-Michael click reaction | None | Centrifugation drive jamming | ^[89]^ |
|  | HA-SH | GRGDSPC | None | Thiol-Michael click reaction | None | Centrifugation drive jamming | ^[90]^ |
|  | HA-SH | GRGDSPC | None | Thiol-Michael click reaction | SH-HA | Thiol-Michael click reaction; Centrifugation drive jamming | ^[91]^ |
| Gelatin and cardiac ECM | GelMA | None | I2959+UV | Free radical polymerization | Human Mesenchymal Stem Cells | Cell-particle adhesion | ^[92]^ |
| Gelatin, siRNA/PEI and bioglass | GelMA | None | LAP +UV | Free radical polymerization | LAP +UV | Free radical polymerization | ^[93]^ |
| Gelatin and PEG (phase separation inducer) | GelMA | None | 4 °C, LAP +UV | Heat-induced physical crosslinking; Free radical polymerization | LAP +UV | Free radical polymerization | ^[94]^ |
| Dextran, β-Cyclodextrin | DexMA, Methacrylated β-Cyclodextrin (MeCD) | Bovine serum albumin-adamantane | LAP+UV | Free radical polymerization | LAP+UV | Free radical polymerization; Host-guest interactions | ^[95]^ |
| HA, poly(ethylene glycol) diacrylate (PEGDA) and agarose | HA-Norb | None | LAP +UV | Thiol-Michael reaction;  Photoinitiated free radical polymerization; Heat-induced physical crosslinking | DDT + Irgacure 2959+UV | Centrifugation or vacuum-drive jamming; Free radical polymerization | ^[96]^ |
| Gelatin and chitosan | GelMA, chitosan oligmer-methacrylate (ChitoMA) | None | UV | Free radical polymerization | None | Electrostatic interactions | ^[97]^ |
| Gelatin and Hydroxyapatite | GelMA, Methacrylated hydroxyapatite (HAp-MA) | None | LAP+UV | Free radical polymerization | LAP+UV | Free radical polymerization | ^[98]^ |
| N-Isopropylacrylamide and N-Isopropylacrylamide | None | None | N, N′-Methylenebis(acrylamide) (BIS), Potassium persulfate (KPS) | Free radical polymerization | None | Electrostatic interactions | ^[99]^ |
| Carboxybetaine acrylamide (CBAA), sulfobetaine methacrylate (SBMA), GelMA, Tyramine acrylamide | None | None | LAP+UV, HRP, H₂O₂ | Free radical polymerization; Enzymatic catalysis | HRP+ H₂O₂ | Enzymatic catalysis | ^[100]^ |
| Acrylamide; Acrylate Alendronate and GelMA | None | None | LAP+UV | Free radical polymerization; Metal ion complexation | Mesoporous Zinc-doped Hydroxyapatite | Electrostatic interactions | ^[101]^ |

**Table S2** Statistics of microsphere characteristics within 5 years

| Composition | Storage modulus or Young's modulus (kPa) | Size | Shape | Ref. |
| --- | --- | --- | --- | --- |
| PEG | 2.5 kPa-165 kPa (Young's modulus) | 25-100 µm | Spherical | ^[10]^ |
| PEG | 6-40 kPa  (Storage modulus) | ≈75 μm | Spherical | ^[9]^ |
| PEG | - | ~50-~100 µm (average 65-70 µm) | Spherical | ^[23]^ |
| PEG | - | 15 µm | Spherical | ^[26]^ |
| PEG | - | ≈150 μm | Spherical | ^[102]^ |
| PEG | ~7-11.35 ± 1.18 kPa (Young's modulus) | 86.26 ± 14.97 µm | Spherical | ^[103]^ |
| PEG | 0.5 kPa; 5.5 kPa; 13.5 kPa  (Storage modulus) | 50 µm; 90 µm | Spherical | ^[2]^ |
| PEG | 9.58 ± 2.30 kPa; 2.37 ± 0.20 kPa  (Storage modulus) | 199.81 ± 95.24 µm  279.31 ± 139.58 µm​ | Spherical | ^[27]^ |
| PEG | 10-82 kPa  (Young's modulus) | 47.9 ± 4.1-146.1 ± 2.7 μm | Spherical | ^[3]^ |
| PEG | 0.096-7.23 kPa  (Storage modulus) | 460-579 μm; 235 μm | Spherical | ^[11]^ |
| PEG | 0.5-2.5 kPa  (Storage modulus) | ~50-100 μm | Spherical | ^[13]^ |
| PEG | 20-22 kPa  (Storage modulus) | 219-270 µm | Spherical | ^[104]^ |
| PEG | ≈28 kPa  (Young's modulus) | ≈135 μm | Spherical | ^[4]^ |
| PEG | 10-40 kPa  (Young's modulus) | 200-600 μm | Spherical | ^[7]^ |
| PEG | - | 355 ± 9 µm | Spherical | ^[105]^ |
| PEG | 1.3 kPa; 7 kPa; 38 kPa  (Storage modulus) | ≈70 μm | Spherical | ^[8]^ |
| PEG | 46 kPa  (Young's modulus) | ∼80 μm | Spherical | ^[24]^ |
| PEG | - | 50 μm | Spherical and crescent-Shaped | ^[15]^ |
| PEG |  | 40-120 μm | Hexagonal-prismatic shape | ^[30]^ |
| PEG | 65 kPa-470 kPa (Young's modulus) | 150 μm | rod-shape | ^[79a]^ |
| Poly(lactic-co-glycolic acid) | 0.55-0.65 kPa  (Storage modulus) | 45-120 µm | Spherical | ^[12]^ |
| Poly(N-vinyl caprolactam) | - | ~96-103 μm | Buckled, hollow, and full supraballs | ^[33]^ |
| Carbomer acrylamide and sulfobetaine methacrylate | - | 98 ± 40 µm; 140 ± 82 µm | Irregular | ^[70]^ |
| Polyethylene glycol diacrylate | - | 250 µm | Cube | ^[29]^ |
| Polyethylene glycol dimethacrylate | - | 200-1000 µm | Hexagons, squares and triangles | ^[106]^ |
| Tyramine Acrylamide, Carboxybetaine Acrylamide, Sulfobetaine Methacrylate | 0.8 kPa; 2 kPa; 3 kPa (Young's modulus) | 120 µm | Irregular | ^[107]^ |
| Matrigel | - | 100-250 µm | Spherical | ^[108]^ |
| Decellularized extracellular matrix | - | 100 µm; 150 µm; 250 µm | Spherical | ^[76]^ |
| Hyaluronic Acid (HA) | - | 207.9 ± 48.21 μm;125.8 ± 35.51 μm; 76.59 ± 20.39 μm | Spherical | ^[82]^ |
| HA | - | 65 µm; 120 µm; 200 µm | Spherical | ^[43]^ |
| HA | 0.882 ± 0.144 kPa (Young's modulus) | 63-82 µm | Spherical | ^[39]^ |
| HA | - | 100 µm | Spherical | ^[109]^ |
| HA | 5-~30 kPa  (Young's modulus) | ~150 μm | Spherical | ^[110]^ |
| HA | 1.810 kPa; 2.591 kPa; 3.646 kPa  (Storage modulus) | 54.25 ± 29.11 μm, 86.04 ± 20.25 μm, 150.76 ± 44.14 μm | Spherical | ^[83]^ |
| HA | - | ~120-300 μm | Spherical | ^[46]^ |
| HA | ~5-30 kPa  (Young's modulus) | ~150 μm | Spherical | ^[47]^ |
| HA | 10-20kPa  (Storage modulus) | ∼100 μm | Spherical, irregular | ^[52]^ |
| HA | 58.3 ± 30.2 kPa (Young's modulus) | 40 µm; 100 µm; 500 µm | Irregular | ^[111]^ |
| HA | 12-80 kPa  (Young's modulus) | 30-100 µm | Irregular | ^[42]^ |
| HA | 11.8 ± 1.4 kPa  (Young's modulus) | 144 ± 94 μm; 48± 20 μm | Irregular | ^[40]^ |
| HA | ~5 kPa  (Young's modulus) | about 40, 70, or 100 μm | Irregular | ^[112]^ |
| HA | 30 kPa  (Young's modulus) | ≈10-300 µm | Irregular | ^[53]^ |
| HA | 17.2 ± 0.9 kPa (Young's modulus) | 261 ± 4 μm; 522 ± 5 μm; 781 ± 9 μm; 1043 ± 7 μm; 1323 ± 32 μm | Rod-shape | ^[45]^ |
| GelMA | ≈ 200 kPa  (Young's modulus) | 80 ± 3 µm | Spherical | ^[60]^ |
| GelMA | - | 100 µm | Spherical | ^[113]^ |
| GelMA | - | 90 µm | Spherical | ^[62]^ |
| GelMA | - | 29 ± 3; 81 ± 4; 173 ± 11 μm | Spherical | ^[64]^ |
| GelMA | 25-107 kPa  (Young's modulus) | ∼70-120 μm | Spherical | ^[114]^ |
| GelMA | 25 kPa  (Young's modulus) | 207.95 μm; 201.58 μm; 183.60 μm | Spherical | ^[115]^ |
| GelMA | 50 kPa  (Young's modulus) | 260 ± 150 μm; 83 ± 31 μm; 48 ± 25 μm | Spherical | ^[59]^ |
| GelMA | - | 187 ± 10 μm | Spherical | ^[94]^ |
| Gelatin | 0.216 Pa-1.257 kPa (Storage modulus) | 100 ± 10 µm | Spherical | ^[86]^ |
| Gelatin | ~90-400 kPa  (Young's modulus) | 1-10 μm | Spherical | ^[56]^ |
| Gelatin | ∼1.5 kPa - ∼7.5 kPa (Storage modulus) | 195.7 ± 1.2 µm; 158.1 ± 1.7 µm; 132.2 ± 1.1 µm; 128.8 ± 1.1 µm | Spherical | ^[65]^ |
| GelMA | - | 150-300 μm | Irregular | ^[93]^ |
| κ-Carrageenan | 7.1-46.3 kPa  (Young's modulus) | 20 μm | Spherical | ^[74]^ |
| Type I collagen | - | ~15 μm; ~62 μm | Spherical | ^[55]^ |
| Chondroitin Sulfate | ∼400 Pa  (Storage modulus) | 153 ± 47 μm; 152 ± 40 μm; 119 ± 30 μm | Irregular | ^[78]^ |
| PEG and Gelatin | - | 200 µm; 400 µm; 1 mm | Spherical, cube, dome, *etc.* | ^[116]^ |
| PEG and Gelatin | 5-10 kPa  (Young's modulus) | 220 μm | Spherical | ^[81]^ |
| PEG and Methacrylates | 1kPa-50 kPa  (Young's modulus) | 304 ± 14 µm | Rod-shape | ^[79b]^ |
| HA and hyperbranched poly(β-hydrazide esters) | 0.1-0.8 kPa  (Storage modulus) | ≈ 80 μm | Irregular | ^[89]^ |
| HA and Type I collagen; | 0.2-1.5 kPa  (Storage modulus) | ≈150 μm | Irregular | ^[88]^ |
| GelMA and Cardiac ECM | - | 77.44- 501.14 μm | Irregular | ^[92]^ |
| GelMA and chitosan oligmer-methacrylate | 1.2 kPa; 3.6 kPa; 4.5 kPa; 12 kPa  (Storage modulus) | 50-310 µm | Spherical | ^[97]^ |
| GelMA and hydroxyapat | 21.62 ± 1.70 kPa; 27.86 ± 2.79 kPa; 36.65 ± 2.24 kPa  (Storage modulus) | 50-200 µm | Spherical | ^[98]^ |
| GelMA, carboxybetaine acrylamide, sulfobetaine methacrylate, and tyramine acrylamide | 5.20 ± 0.25 kPa  5.64 ± 0.33 kPa (Young's modulus) | 59 ± 44 µm; 130 ± 100 µm | Irregular | ^[117]^ |
| GelMA, acrylate alendronate (ALNAc) and acrylamide (AAm) | - | Rod: 399.1 ± 10.6 µm  Spherical: ~400 μm | Rod-shape, Spherical | ^[101]^ |
| CBAA and SBMA | - | 59 ± 45 µm; 129 ± 91 µm | Irregular | ^[100]^ |
| Dextran and β-cyclodextrin | 2.2 kPa; 29.1 kPa (Storage modulus) | 215.4 ± 109.7 μm; 34.1 ± 17.5 μm; 32.4 ± 16.4 μm | Irregular | ^[95]^ |

**Table S3** Statistics of pore characteristics of granular hydrogel over past 5 years

| Size of microgels | Porosity (%) | Pore area (µm²) | Pore size (μm) | Ref. |
| --- | --- | --- | --- | --- |
| 47 ± 4 µm; 49 ± 6 µm; 43 ± 5 µm | 15%-18% | ~100 µm² | ∼10 µm | ^[61]^ |
| 50 μm | 40-48% | 10-10000 um^2^ | - | ^[15]^ |
| 52 ± 14 µm | ∼18-22% | - | ∼20 μm | ^[113]^ |
| ≈70 μm | 34%-46% | - | 19-36 μm | ^[8]^ |
| 75 μm | 34%-46% | - | 19 μm; 23 μm; 36 μm | ^[8]^ |
| 80 ± 3 μm | 25-30% | - | ≈21-23 µm | ^[60]^ |
| 90 ± 3 µm | 7 ± 0.02 %-19 ± 0.01 % | 100-200 µm^2^ | 16.5 ± 0.5 μm; 14.6 ± 0.7 μm; 15.1 ± 1.1 μm; 9.2 ± 1.8 μm | ^[62]^ |
| 95 µm | - | 162 µm^2^ | 14.4 µm | ^[17]^ |
| 110 ± 23.9 μm | - | 100-2500 μm² | 10-50 μm | ^[14]^ |
| 118 ± 1.00 μm | 8-33% | - | - | ^[118]^ |
| 120 µm | 5-40% | - | - | ^[107]^ |
| ≈140μm | ≈20%-30% | ~750-3000 μm^2^ | - | ^[49]^ |
| ~150 μm | ~8-14% | - | - | ^[47]^ |
| 187 ± 10 μm | 18 ± 2%-49 ± 1% | - | 16 ± 1-33 ± 2 μm | ^[94]^ |
| 209.7 ± 106.6 μm | 3± 2%; 13 ± 4%; 29 ± 7%; 35± 7%; 33± 7% | - | - | ^[119]^ |
| 220 µm | 25-30% | 2500-3500 µm² | - | ^[81]^ |
| ≈10-50 μm (Average 20 μm) | ≈20-40 % | - | - | ^[74]^ |
| ≈10-300 µm | 12% | - | 10-100 µm | ^[53]^ |
| 14.8 ± 0.22 μm; 132.6 ± 0.31 μm | ~12-~30% | - | 36.63 μm; 9.7 μm | ^[22]^ |
| 15-16 µm; 120-130 µm | 12% ± 2%;  29% ± 3% | 20-3300 µm² | 6.1-120 µm | ^[16]^ |
| ~15 μm; ~62 μm | 26.6% ± 5.8%; 15.7% ± 3.9% | - | - | ^[55]^ |
| 22 ± 10 μm; 34 ± 15 μm | 4.8%-12.2% | - | - | ^[87]^ |
| 20.8 μm; 40.7 μm ; 58.4 μm | 26%-32% | 70 µm²;120 µm²;180 µm² | - | ^[20]^ |
| 29 ± 3; 81 ± 4; 173 ± 11 μm | ~20-25% | - | 12 ± 2, 20 ± 2, or 44 ± 3 μm | ^[64]^ |
| 40 μm; 70 μm; 100 μm | 10% -35% | - | - | ^[112]^ |
| 40 μm;70 μm;130 μm | 25%-35% | ~10-80 µm² |  | ^[120]^ |
| 40 µm;100 µm; 500 µm | 21.5 % ± 6.8%; 14.3% ± 4.1%; 9.2% ± 3.8% | 175.7 ± 289.8 µm²; 2671.5 ± 9700.9 µm²;3416.8 ± 6562.2 µm² | - | ^[111]^ |
| 45-120 μm | 15% | - | ≈20 µm | ^[12]^ |
| 48 ± 20µm; 144 ± 94 μm | 29 ± 3%  36 ± 5% | - | - | ^[40]^ |
| 50 μm; 100 μm; 150 μm; | ~17-22% | - | ~40-90 μm | ^[91]^ |
| 50 μm; 80 μm; 200 μm | - | ~20-300 µm² | 8.5 pL; 26.4 pL; 523 pL | ^[51]^ |
| 50-200 µm | - | 25-3600 μm² | 5-60 μm | ^[98]^ |
| 54.25 ± 29.11μm; 86.04 ± 20.25μm; 150.76 ± 44.14μm | 22-25% | - | - | ^[83]^ |
| 59 ± 45 µm;129 ± 91 µm | 13% ± 6%-20% ± 6% | 1282 ± 784 µm²; 2167 ± 1281 µm² (≈300 - 4000 µm²) | - | ^[100]^ |
| ≈59 ± 44 µm; ≈130 ± 100 µm | 13.99 ± 4.60 %; 14.51 ± 3.99%; 19.81 ± 5.08%; 21.18 ± 4.74% | 400-900 µm² | ≈20-30 μm | ^[117]^ |
| ∼65 μm; ∼120 μm;∼200 μm | ~2% - ~ 40% | ~100-~10^5^ µm² | - | ^[43]^ |
| ∼70-120 μm | ~14-~16% | ~440 µm² | ~22 μm | ^[114]^ |
| ≈77.44-501.14 μm | 77.55 ± 3.70% - 43.03 ± 1.35% | - | - | ^[92]^ |
| 98 ± 40 µm; 140 ± 82 µm | 15 ± 2%- 20±1.4% | - | - | ^[70]^ |
| <100 µm;  >100 µm (≈100-300 µm) | ≈ 5%- ≈ 20% | ≈ 10^1^-10^4^ µm² | - | ^[42]^ |
| 100 µm; 150 µm; 100×220 µm | ≈15% -≈25% | ≈10^1^-10^5^ µm² | - | ^[44]^ |
| 100 μm;150 μm; 200 μm; 250μm | 75%-64% | - | - | ^[108]^ |
| ∼ 100 µm; 20-200 μm | ~3%-~8% | ~10-10000 µm² | - | ^[52]^ |
| 119 ± 30 µm; 153 ± 47; 152 ± 40 μm | ~9%; ~21% | - | ~150 μm; ~380 μm | ^[78]^ |
| ≈120-300 μm | ~10%-28% | ~600-10000 μm² | - | ^[46]^ |
| 126 ± 31-155 ± 37 μm | - | 611-3290 μm^2^ | - | ^[48]^ |
| 128.8 µm ± 1.1 µm; 158.1 ± 1.7 µm; 132.2 ± 1.1 µm; 195.7 µm ± 1.2 µm | 20-40% | - | - | ^[65]^ |
| 146.1 ± 2.7 μm; 47.9 ± 4.1 μm | - | 200-800 µm² | - | ^[3]^ |
| 199.81 ± 95.24 μm ~ 279.31 ± 139.58 μm | 42.4 ± 10.1%; 39.7 ± 2.80%; 41.5 ± 4.50%; 47.4 ± 2.75% | 2690± 6730-1630 ± 5210 μm^2^  2320 ± 8230-1310 ± 1900 μm^2^ | - | ^[27]^ |
| 261 ± 4 μm; 522 ± 5 μm; 781 ± 9 μm; 1043 ± 7 μm; 1323 ± 32 μm | ~13%-16% | ~900-1200 μm^2^ | - | ^[45]^ |
| 10 × 10 × 50 μm^3^; 10 × 10 × 100 μm^3^; 10 × 10 × 200 μm^3^ | **65%; 70%; 90%** | - | 39± 7 μm; 50 ± 2 μm; 82 ± 2 μm | ^[79a]^ |
| 143 ± 8 μm ×123 ± 4 μm  154 ± 4 μm × 109 ± 3 μm  304 ± 14 μm | 20% -50%  40% -70% | - | ≈ 30-150 μm  ≈10 - 55 μm | ^[79b]^ |

**Table S4** Statistics of mechanical properties of granular hydrogel over past 5 years

| Composition | Secondary crosslinking | Degradability | Storage modulus | Stiffness variables | Ref. |
| --- | --- | --- | --- | --- | --- |
| PEG | Host-guest interactions | Alkaline conditions (pH8-13) | Bulk: 84-7230 Pa  MAP: 55-115 Pa | Cross-linking group concentration and condition; | ^[11]^ |
| PEG | Enzymatic catalysis | MMP-triggered degradability | MAP: 500-2500 Pa | MMP-cleavable crosslinkers concentration | ^[13]^ |
| PEG | Thiol-ene click reaction | MMP-triggered degradability | JGH: 150 Pa  MAP (spherical): 550 Pa  MAP (crescent): 580 Pa | Microgel shape | ^[15]^ |
| PEG | Thiol-ene click reaction | MMP-triggered degradable | MAP: 600 Pa | Degradable peptide crosslinker | ^[28]^ |
| PEG | iEDDA reaction and thiol-ene click reaction | MMP-triggered degradable | JGH: 1160 Pa  MAP: 1970 Pa | Tetrazine -crosslinkers concentration | ^[32]^ |
| PEG | iEDDA reaction and thiol-ene click reaction | MMP-triggered degradable | MAP (5 kDa;thiol-ene): 1370 ± 200 Pa  MAP (5 kDa;iEDDA): 2620 ± 560 Pa  MAP (20 kDa;thiol-ene): 640 ± 80 Pa  MAP (20 kDa;iEDDA): 700 ± 200 Pa | Annealing strategy;  Mw of PEG | ^[27]^ |
| PEG | Guest - host reaction | - | Inter-MAP: 1330 Pa  MAP: 1330 Pa  Intra-MAP: 530 Pa | Cross-linking group position | ^[18]^ |
| PEG | Thiol-ene click reaction | - | JGH: 1000 Pa  MAP: 1600 Pa | Annealing | ^[7]^ |
| PEG | Free radical polymerization | - | MAP (Mw 5): 1400-1600 Pa  MAP (Mw 20): 200 Pa | Mw of PEG  Linker and initiator concentrations | ^[121]^ |
| PEG | Thiol-ene click reaction | MMP-triggered degradable | JGH: ~1200 Pa  MAP: ~4000 Pa | Annealing | ^[14]^ |
| PEG | iEDDA reaction | MMP-triggered degradable | JGH: ~175 Pa  MAP: ~438 Pa | Annealing | ^[26]^ |
| PEG | Thiol-ene click reaction | Plasmin- triggered degradable | MAP (20 μm): 30,000 Pa  MAP (40 μm): 84,100 Pa  MAP (60 μm): 191,0000 Pa | Microgel size | ^[20]^ |
| HA | iEDDA reaction | MMP-triggered degradable | MAP: 161-5846 Pa | Cross-linking group ratio | ^[122]^ |
| HA | Hydrazide- aldehyde condensation Reaction | - | JGH: 2000 Pa  MAP: 9000 Pa | Annealing | ^[53]^ |
| HA | Enzymatic catalysis | MMP-triggered degradable | JGH: ~400 Pa  MAP: ~500-600 Pa | Annealing | ^[112]^ |
| HA | Thiol-ene click reaction | - | JGH: 1200-1600 Pa  MAP: 1300 -2200 Pa | Annealing | ^[3]^ |
| HA | Enzymatic Crosslinking | Enzymatic degradation | MAP (20 μm):3924 ± 210 Pa  MAP (150 μm):3156 ± 68 Pa | Microgel size | ^[40]^ |
| HA | Thiol-ene click reaction | - | MAP (spherical): 75-175 Pa  MAP (irregular): 1000-3000 Pa | Microgel shape | ^[52]^ |
| GelMA | Free radical polymerization | - | MAP (5%): 20,000 ± 300 Pa  MAP (10%): 29,000 ± 600 Pa MAP (20%): 39,000 ± 900 Pa | GelMA concentration | ^[61]^ |
| GelMA | Free radical polymerization | - | Bulk: 1000-5000 Pa  MAP: 100-1000 Pa | Cross-linking time | ^[114]^ |
| GelMA | Free radical polymerization | - | MAP (3000×g-15s): 6600 ± 600 Pa  MAP (16000×g-300s): 12100 ± 1300 Pa | Packing density | ^[62]^ |
| GelMA | Free radical polymerization | - | MAP (nonporous): ~20,000 Pa  MAP (small-pore): ~11,000 Pa  MAP (large-pore): ~16,000 Pa | Microgel pore size | ^[94]^ |
| GelMA | Electrostatic interactions | - | Bulk: ≈ 30,000 Pa  MAP: ≈ 10,000 Pa | - | ^[60]^ |
| GelMA | Free radical polymerization | - | Bulk: ≈ 40,000 Pa;  MAP (small and medium): ≈ 40,000 Pa;  MAP (large) : ≈ 10,000 Pa; | Annealing and microgel size | ^[64]^ |
| GelMA | Free radical polymerization | - | JGH: 50-100 Pa; MAP: 2000-4000 Pa | Annealing | ^[59]^ |
| Type I collagen | physically driven self-assembly | - | MAP (10% TA@nHA): ≈ 8000 Pa  MAP (20% TA@nHA): ≈ 15,000 Pa  MAP (30% TA@nHA): ≈ 22,000 Pa | Linker concentrations | ^[55]^ |
| PEG and HA | iEDDA reaction | - | MAP: 278-2016 Pa | Cross-linking group ratio | ^[82]^ |
| PEG and HA | iEDDA reaction | - | Bulk: 1810-3646 Pa  MAP: 222-970 Pa | Microgel size; Cross-linking group ratio | ^[83]^ |
| PEG and HA | Thiol-ene click reaction | MMP-triggered degradable | PEG MAP: 395 Pa; 410 Pa and 744 Pa  HA MAP: 181 Pa;724 Pa and 1030 Pa | Cross-linking group ratio | ^[118]^ |
| Gelatin and acrylamide | Metal-ligand interactions | - | MAP (spherical): 250 Pa;  MAP (rod) : 600 Pa ;  MAP (mesoporous Zinc-doped hydroxyapatite,mZH) : 1750 Pa;  MAP (Catalase-loaded mZH) : 1250 Pa | Microgel shape and adhesion medium | ^[101]^ |
| GelMA and Alginate | Free radical polymerization | - | MAP (GelMA:AlgOx 100): 483 ± 129 Pa;  MAP (GelMA:AlgOx 50): 1405 ± 452 Pa | Alginate concentration | ^[119]^ |
| GelMA and hydroxyapat | Free radical polymerization | - | JGH: 80 Pa  MAP: 20,000 Pa | Annealing | ^[98]^ |
| HA and poly(β-hydrazide esters) | Thiol–Michael click reaction | - | JGH: 600 Pa  MAP: 3400 Pa | Annealing | ^[91]^ |
| HA and mesenchymal stromal cell spheroids | Cell-particle adhesion | - | JGH: ≈1000 Pa  Jammed MSC spheroids:  ≈100 Pa  Granular hydrogel with MSC spheroids: ≈800-1000 Pa | MSC spheroids and microgels ratio | ^[49]^ |
| PEG | No | - | 420 Pa | - | ^[25]^ |
| PEG | No | - | JGH (soft): 80 Pa  JGH (stiff): 3650 Pa  JGH (25 μm): 1800 Pa  JGH (100 μm): 5500 Pa | Microgel stiffness and packing density | ^[10]^ |
| HA | No | - | JGH (spheres): 250-270 Pa  JGH (rods): 590 Pa | Packing density | ^[44]^ |
| HA | No | - | High packing: 1500-2500 Pa  Medium packing: 1000-2000 Pa  Low packing: 1000 Pa | Packing density | ^[42]^ |
| HA | No | Enzymatic degradation | JGH (0.5%): ~80 Pa  JGH (0.8%): ~200 Pa  JGH (1.0%): ~500 Pa  JGH (1.2%): ~800-1000 Pa | Linker concentrations | ^[37]^ |
| HA | No | - | JGH (Low packing;120 μm): 80 Pa  JGH (High packing;120 μm): 270 Pa  JGH (Very high packing;120 μm): 520 Pa  JGH (65 μm): 480 Pa  JGH (120 μm): 340 Pa  JGH (200 μm): 250 Pa | Packing density and microgel size | ^[43]^ |
| HA | No | - | JGH (1 wt.%): 77 ± 3 Pa  JGH (2 wt.%): 234 ± 2 Pa | NorHA concentration | ^[123]^ |
| Agarose | No | - | JGH (0.5%):175-1000Pa  JGH (1%): 2-3000 Pa  JGH (2%):438 -8000Pa | Agarose concentration | ^[73]^ |
| κ-Carrageenan | No | - | Low packing density: 642-1591 Pa  Medium packing density: 3164 Pa  High packing density: 3420-3928 Pa | Packing density | ^[74]^ |
| GelMA and cardiac ECM | No | - | JGH: 3.21 ± 0.99 Pa  JGH (hMSC 1day): 35.79 ± 6.83 Pa  JGH (hMSC 3day): 40.78 ± 7.34 Pa | Cells growth | ^[92]^ |
| HA and chitosan | No | - | JGH (0.15 M NaCl): ~ 30,000 Pa  JGH (0.5 M NaCl): ~4,000 Pa  JGH (1.5 M NaCl): ~3,000 Pa | Polyelectrolyte concentration | ^[87]^ |

**Note:** Bulk refers to bulk hydrogel; MAP indicates microporous annealed particle; and JGH indicates jammed granular hydrogel.

**Reference:**

1. Liu, Y. Suarez-Arnedo, A. Caston, E., et al., "Exploring the Role of Spatial Confinement in Immune Cell Recruitment and Regeneration of Skin Wounds" *Advanced Materials* (2023): 35, e2304049.

2. de Rutte, J. Koh, J. and D. Di Carlo, "Scalable High‐Throughput Production of Modular Microgels for In Situ Assembly of Microporous Tissue Scaffolds" *Advanced Functional Materials* (2019): 29, 1900071.

3. Lowen, J. Bond, G. Griffin, K., et al., "Multisized Photoannealable Microgels Regulate Cell Spreading, Aggregation, and Macrophage Phenotype through Microporous Void Space" *Advanced Healthcare Materials* (2023): 12, e2202239.

4. Casella, A. Lowen, J. Griffin, K., et al., "Conductive Microgel Annealed Scaffolds Enhance Myogenic Potential of Myoblastic Cells" *Advanced Healthcare Materials* (2024): 13, e2302500.

5. Roam, J. Yan, Y. Nguyen, P., et al., "A modular, plasmin-sensitive, clickable poly(ethylene glycol)-heparin-laminin microsphere system for establishing growth factor gradients in nerve guidance conduits" *Biomaterials* (2015): 72, 112.

6. Dumont, C. Carlson, M. Munsell, M., et al., "Aligned hydrogel tubes guide regeneration following spinal cord injury" *Acta Biomaterialia* (2019): 86, 312.

7. Xin, S. Chimene, D. Garza, J. Gaharwar, A. and D. Alge, "Clickable PEG hydrogel microspheres as building blocks for 3D bioprinting" *Biomaterials Science* (2019): 7, 1179.

8. Krattiger, L. Emiroglu, D. Pravato, S., et al., "Microfluidic Platforms to Screen Granular Hydrogel Microenvironments for Tissue Regeneration" *Advanced Functional Materials* (2024): 34, 2310507.

9. Emiroglu, D. Singh, A. Marco-Dufort, B., et al., "Granular Biomaterials as Bioactive Sponges for the Sequestration and Release of Signaling Molecules" *Advanced Healthcare Materials* (2024): 13, e2400800.

10. Dilara B., Aleksandar, B. Dalia, D., et al. "Building block properties govern granular hydrogel mechanics through contact deformations" *Science advances* (2022): 8, eadd8570.

11. Feliciano, A. Alaoui Selsouli, Y. Habibovic, P., et al., "Granular polyrotaxane microgels as injectable hydrogels for corneal tissue regeneration" *Biomaterials Science* (2024): 12, 4993.

12. Fang, J. Koh, J. Fang, Q., et al., "Injectable Drug-Releasing Microporous Annealed Particle Scaffolds for Treating Myocardial Infarction" *Advanced Functional Materials* (2020): 30, 2004307.

13. Koh, J. Griffin, D. R. Archang, M., et al., "Enhanced In Vivo Delivery of Stem Cells using Microporous Annealed Particle Scaffolds" *Small* (2019): 15, e1903147.

14. Mayer, D. P. Nelson, M. E. Andriyanova, D., et al., "A novel microporous biomaterial vaccine platform for long-lasting antibody mediated immunity against viral infection" *Journal of Controlled Release* (2024): 370, 570.

15. T Tang, R. Shang, L. Scumpia, P. and D. Di Carlo, "Injectable Microporous Annealed Crescent-Shaped (MAC) Particle Hydrogel Scaffold for Enhanced Cell Infiltration" *Advanced Healthcare Materials* (2024): 13, e2302477.

16. Caldwell, A. Campbell, G. Shekiro, K. and K. Anseth, "Clickable Microgel Scaffolds as Platforms for 3D Cell Encapsulation" *Advanced Healthcare Materials* (2017): 6, 1700254.

17. Pruett, L. Kenny, H. Swift, W., et al., "De novo tissue formation using custom microporous annealed particle hydrogel provides long-term vocal fold augmentation" *NPJ Regenerative Medicine* (2023): 8, 10.

18. Widener, A. Roberts, A. and E. Phelps, "Single versus dual microgel species for forming guest-host microporous annealed particle PEG-MAL hydrogel" *Journal of Biomedical Materials Research Part A* (2023): 111, 1379.

19. Pruett, L. Koehn, H. Martz, T., et al., "Development of a microporous annealed particle hydrogel for long-term vocal fold augmentation" *Laryngoscope* (2020): 130, 2432.

20. Ross, B. Kent, R., III Saunders, R., et al., "Building-Block Size Mediates Microporous Annealed Particle Hydrogel Tube Microenvironment Following Spinal Cord Injury" *Advanced Healthcare Materials* (2024): 13, e2302498.

21. Pruett, L. Taing, A. Singh, N. Peirce, S. and D. Griffin, "In silico optimization of heparin microislands in microporous annealed particle hydrogel for endothelial cell migration" *Acta Biomaterialia* (2022): 148, 171.

22. Widener, A. Duraivel, S. Angelini, T. and E. Phelps, "Injectable Microporous Annealed Particle Hydrogel Based on Guest-Host-Interlinked Polyethylene Glycol Maleimide Microgels" *Advanced NanoBiomed Research* (2022): 2, 2200030.

23. Roosa, C. Lempke, S. Hannan, R., et al., "Conjugation of IL-33 to Microporous Annealed Particle Scaffolds Enhances Type 2-Like Immune Responses In Vitro and In Vivo" *Advanced Healthcare Materials* (2024): 13, e2400249.

24. Pfaff, B. Pruett, L. Cornell, N., et al., "Selective and Improved Photoannealing of Microporous Annealed Particle (MAP) Scaffolds" *ACS Biomaterials Science & Engineering* (2021): 7, 422.

25. Claxton, N. Luse, M. Isakson, B. and C. Highley, "Engineering Granular Hydrogels without Interparticle Cross-Linking to Support Multicellular Organization" *ACS Biomaterials Science & Engineering* (2024): 10, 7594.

26. Tigner, T. Dampf, G. Tucker, A., et al., "Clickable Granular Hydrogel Scaffolds for Delivery of Neural Progenitor Cells to Sites of Spinal Cord Injury" *Advanced Healthcare Materials* (2024): 13, e2303912.

27. Recalde Phillips, S. Perez-Ponce, K. Ruben, E., et al., "Impact of Annealing Chemistry on the Properties and Performance of Microporous Annealed Particle Hydrogels" *Biomacromolecules* (2024): 25, 5798.

28. Xin, S. Gregory, C. A. Alge, D. L., "Interplay between degradability and integrin signaling on mesenchymal stem cell function within poly(ethylene glycol) based microporous annealed particle hydrogels" *Acta Biomaterialia* (2020): 101, 227.

29. Qi, H. Ghodousi, M. Du, Y., et al., "DNA-directed self-assembly of shape-controlled hydrogels" *Nature Communications* (2013): 4, 2275.

30. Du, H. Cont, A. Steinacher, M. and E. Amstad, "Fabrication of Hexagonal-Prismatic Granular Hydrogel Sheets" *Langmuir* (2018): 34, 3459.

31. Li, C. Wood, D. Hsu, C. and S. Bhatia, "DNA-templated assembly of droplet-derived PEG microtissues" *Lab on a Chip* (2011): 11, 2967.

32. Isaac, A. Jivan, F. Xin, S., et al., "Microporous Bio-orthogonally Annealed Particle Hydrogels for Tissue Engineering and Regenerative Medicine" *ACS Biomaterials Science & Engineering* (2019): 5, 6395.

33. Jung, S. Meyer, F. Hornig, S., et al., "On-Chip Fabrication of Colloidal Suprastructures by Assembly and Supramolecular Interlinking of Microgels" *Small* (2024): 20, e2303444.

34. Harada, A. Kobayashi, R. Takashima, Y. Hashidzume, A. and H. Yamaguchi, "Macroscopic self-assembly through molecular recognition" *Nature Chemistry* (2011): 3, 34.

35. Hirsch, M. Charlet, A. Amstad, E., "3D Printing of Strong and Tough Double Network Granular Hydrogels" *Advanced Functional Materials* (2020): 31, 2005929.

36. Milani, A. Freemont, A. Hoyland, J. Adlam, D. and B. Saunders, "Injectable doubly cross-linked microgels for improving the mechanical properties of degenerated intervertebral discs" *Biomacromolecules* (2012): 13, 2793.

37. Zhang, C. Cheng, Z. Zhou, Y., et al., "The novel hyaluronic acid granular hydrogel attenuates osteoarthritis progression by inhibiting the TLR-2/NF-kappaB signaling pathway through suppressing cellular senescence" *Bioengineering & Translational Medicine* (2023): 8, e10475.

38. Karam, J. Singer, B. Miwa, H., et al., "Molecular weight of hyaluronic acid crosslinked into biomaterial scaffolds affects angiogenic potential" *Acta Biomaterialia* (2023): 169, 228.

39. Sideris, E. Griffin, D. Ding, Y., et al., "Particle Hydrogels Based on Hyaluronic Acid Building Blocks" *ACS Biomaterials Science & Engineering* (2016): 2, 2034.

40. Puiggali-Jou, A. Asadikorayem, M. Maniura-Weber, K. and M. Zenobi-Wong, "Growth factor-loaded sulfated microislands in granular hydrogels promote hMSCs migration and chondrogenic differentiation" *Acta Biomaterialia* (2023): 166, 69.

41. Nih, L. Sideris, E. Carmichael, S. T. Segura, T., "Injection of Microporous Annealing Particle (MAP) Hydrogels in the Stroke Cavity Reduces Gliosis and Inflammation and Promotes NPC Migration to the Lesion" *Advanced Materials* (2017): 29, 1606471.

42. Tanner, G. Schiltz, L. Narra, N. Figueiredo, M. and T. Qazi, "Granular Hydrogels Improve Myogenic Invasion and Repair after Volumetric Muscle Loss" *Advanced Healthcare Materials* (2024): 13, e2303576.

43. Qazi, T. Muir, V. and J. Burdick, "Methods to Characterize Granular Hydrogel Rheological Properties, Porosity, and Cell Invasion" *ACS Biomaterials Science & Engineering* (2022): 8, 1427.

44. Qazi, T. Wu, J. Muir, V., et al., "Anisotropic Rod-Shaped Particles Influence Injectable Granular Hydrogel Properties and Cell Invasion" *Advanced Materials* (2022): 34, e2109194.

45. Stornello, D. Kim, J. Chen, Z. Heaton, K. and T. Qazi, "Controlling Microparticle Aspect Ratio via Photolithography for Injectable Granular Hydrogel Formation and Cell Delivery" *ACS Biomaterials Science & Engineering* (2025): 11, 1242.

46. Montes, D. Saha, S. Taglione, A., et al., "Tuning the Morphological Properties of Granular Hydrogels to Control Lymphatic Capillary Formation" *Advanced Materials Interfaces* (2025): 202401037.

47. Di Caprio, N. Hughes, A. and J. Burdick, "Programmed shape transformations in cell-laden granular composites" Science advances (2025): 11, eadq5011.

48. Nakamura, K. Di Caprio, N. and J. Burdick, "Engineered Shape-Morphing Transitions in Hydrogels Through Suspension Bath Printing of Temperature-Responsive Granular Hydrogel Inks" *Advanced Materials* (2024): 36, e2410661.

49. Caprio, N. Davidson, M. Daly, A. and J. Burdick, "Injectable MSC Spheroid and Microgel Granular Composites for Engineering Tissue" *Advanced Materials* (2024): 36, e2312226.

50. Mealy, J. Chung, J. Jeong, H., et al., "Injectable Granular Hydrogels with Multifunctional Properties for Biomedical Applications" *Advanced Materials* (2018): 30, e1705912.

51. Anderson, A. Caston, E. Riley, L., et al., "Engineering the Microstructure and Spatial Bioactivity of MAP Scaffolds Instructs Vasculogenesis In Vitro and Modifies Vessel Formation In Vivo" *Advanced Functional Materials* (2024): 35, 2400567.

52. Muir, V. Qazi, T. Shan, J. Groll, J. and J. Burdick, "Influence of Microgel Fabrication Technique on Granular Hydrogel Properties" *ACS Biomaterials Science & Engineering* (2021): 7, 4269.

53. Muir, V. Qazi, T. Weintraub, S., et al., "Sticking Together: Injectable Granular Hydrogels with Increased Functionality via Dynamic Covalent Inter-Particle Crosslinking" *Small* (2022): 18, e2201115.

54. Matsunaga, Y. Morimoto, Y. and S. Takeuchi, "Molding cell beads for rapid construction of macroscopic 3D tissue architecture" *Advanced Materials* (2011): 23, H90.

55. Li, Y. Bai, X. Ren, C. Ma, Y. and Y. Liu, "Construction of injectable collagen-microgel/tannic acid/nano-hydroxyapatite granular hydrogel and evaluation of its potential in wound healing" *Journal of Bioactive and Compatible Polymers* (2023): 38, 325.

56. Li, S. Niu, D. Fang, H., et al., "Tissue adhesive, ROS scavenging and injectable PRP-based 'plasticine' for promoting cartilage repair" *Regenerative Biomaterials* (2024): 11, rbad104.

57. Imparato, G. Urciuolo, F. Casale, C. and P. Netti, "The role of microscaffold properties in controlling the collagen assembly in 3D dermis equivalent using modular tissue engineering" *Biomaterials* (2013): 34, 7851.

58. Nair, S. Basu, S. Sen, B., et al., "Colloidal Gels with Tunable Mechanomorphology Regulate Endothelial Morphogenesis" *Scientific Reports* (2019): 9, 1072.

59. ) Molley, T. Hung, T. and K. Kilian, "Cell-Laden Gradient Microgel Suspensions for Spatial Control of Differentiation During Biofabrication" *Advanced Healthcare Materials* (2022): 11, e2201122.

60. Ataie, Z. Kheirabadi, S. Zhang, J., et al., "Nanoengineered Granular Hydrogel Bioinks with Preserved Interconnected Microporosity for Extrusion Bioprinting" *Small* (2022): 18, e2202390.

61. Carvalho, B. Nakayama, A. Miwa, H., et al., "Gelatin methacryloyl granular scaffolds for localized mRNA delivery" *Aggregate (Hoboken)* (2024): 5, e464.

62. Jaberi, A. Kedzierski, A. Kheirabadi, S., et al., "Engineering Microgel Packing to Tailor the Physical and Biological Properties of Gelatin Methacryloyl Granular Hydrogel Scaffolds" *Advanced Healthcare Materials* (2024): 13, e2402489.

63. Sheikhi, A. de Rutte, J. Haghniaz, R., et al., "Modular microporous hydrogels formed from microgel beads with orthogonal thermo-chemical responsivity: Microfluidic fabrication and characterization" *MethodsX* (2019): 6, 1747.

64. Ataie, Z. Horchler, S. Jaberi, A., et al., "Accelerating Patterned Vascularization Using Granular Hydrogel Scaffolds and Surgical Micropuncture" *Small* (2024): 20, e2307928.

65. Chang, C. Y. Nguyen, H. Frahm, E. Kolaczyk, K. and C. Lin, "Triple click chemistry for crosslinking, stiffening, and annealing of gelatin-based microgels" *RSC Applied Polymers* (2024): 2, 656.

66. Cai, B. Zou, Q. Zuo, Y., et al., "Injectable Gel Constructs with Regenerative and Anti-Infective Dual Effects Based on Assembled Chitosan Microspheres" *ACS Applied Materials & Interfaces* (2018): 10, 25099.

67. Tedesco, M. Di Lisa, D. Massobrio, P., et al., "Soft chitosan microbeads scaffold for 3D functional neuronal networks" *Biomaterials* (2018): 156, 159.

68. a) Jeon, O. Bin Lee, Y. Hinton, T. Feinberg, A. and E. Alsberg, "Cryopreserved cell-laden alginate microgel bioink for 3D bioprinting of living tissues" *Materials Today Chemistry* (2019): 12, 61; b) Jeon, O. Lee, Y. B. Jeong, H., et al., "Individual cell-only bioink and photocurable supporting medium for 3D printing and generation of engineered tissues with complex geometries" *Materials Horizons* (2019): 6, 1625.

69. Hu, Y. Mao, A. S. Desai, R., et al., "Controlled self-assembly of alginate microgels by rapidly binding molecule pairs" *Lab on a Chip* (2017): 17, 2481.

70. Surman, F. Asadikorayem, M. Weber, P. Weber, D. and M. Zenobi-Wong, "Ionically annealed zwitterionic microgels for bioprinting of cartilaginous constructs" *Biofabrication* (2024): 16, 025004.

71. Van Tomme, S. van Nostrum, C. Dijkstra, M. De Smedt, S. and W. Hennink, "Effect of particle size and charge on the network properties of microsphere-based hydrogels" *European Journal of Pharmaceutics and Biopharmaceutics* (2008): 70, 522.

72. Bulut, S. Gunther, D. Bund, M., et al., "Cellular Architects at Work: Cells Building their Own Microgel Houses" *Advanced Healthcare Materials* (2024): 13, e2302957.

73. Britchfield, G. and A. Daly, "Bioprinting in granular support hydrogels - characterizing the role of particle morphology and packing density" *Biofabrication* (2025): 17, 025004..

74. Hen, N. Josef, E. Davidovich-Pinhas, M. Levenberg, S. and H. Bianco-Peled, "On the Relation between the Viscoelastic Properties of Granular Hydrogels and Their Performance as Support Materials in Embedded Bioprinting" *ACS Biomaterials Science & Engineering* (2024): 10, 6734.

75. Merindol, R. Loescher, S. Samanta, A. and A. Walther, "Pathway-controlled formation of mesostructured all-DNA colloids and superstructures" *Nature Nanotechnology* (2018): 13, 730.

76. Im, P. Shin, H. and J. Kim, "Tilapia-Derived Granular Hydrogel as a 3D Scaffold Promoting Rapid Wound Healing" *Biomacromolecules* (2024): 25, 1153.

77. Chen, J. Li, Q. Li, H., et al., "Injectable acellular matrix microgel assembly with stem cell recruitment and chondrogenic differentiation functions promotes microfracture-based articular cartilage regeneration" *Bioactive Materials* (2025): 44, 220.

78. Lee, H. Davis, R., Jr. Wang, T., et al., "Dynamically Cross-Linked Granular Hydrogels for 3D Printing and Therapeutic Delivery" *ACS Applied Bio Materials* (2023): 6, 3683.

79. a) Suturin, A. Kruger, A. Neidig, K., et al., "Annealing High Aspect Ratio Microgels into Macroporous 3D Scaffolds Allows for Higher Porosities and Effective Cell Migration" *Advanced Healthcare Materials* (2022): 11, e2200989; b) Rommel, D. Mork, M. Vedaraman, S., et al., "Functionalized Microgel Rods Interlinked into Soft Macroporous Structures for 3D Cell Culture" *Advance Science (Weinh)* (2022): 9, e2103554.

80. Li, F. Truong, V. Fisch, P., et al., "Cartilage tissue formation through assembly of microgels containing mesenchymal stem cells" *Acta Biomaterialia* (2018): 77, 48.

81. Wang, S. Wang, K. Cao, W., et al., "Treg-enhancing and immunomodulating microgel scaffold promotes cell ingrowth and heart function recovery post-acute myocardial infarction in vivo" *Chemical Engineering Journal* (2024): 497, 154933.

82. Darling, N. Xi, W. Sideris, E., et al., "Click by Click Microporous Annealed Particle (MAP) Scaffolds" *Advanced Healthcare Materials* (2020): 9, e1901391.

83. Truong, N. Kurt, E. Tahmizyan, N., et al., "Microporous annealed particle hydrogel stiffness, void space size, and adhesion properties impact cell proliferation, cell spreading, and gene transfer" *Acta Biomaterialia* (2019): 94, 160.

84. Zhang, X. Li, Y. He, D., et al., "An effective strategy for preparing macroporous and self-healing bioactive hydrogels for cell delivery and wound healing" *Chemical Engineering Journal* (2021): 425, 130677.

85. Zhu, Y. Sun, Y. Rui, B., et al., "A Photoannealed Granular Hydrogel Facilitating Hyaline Cartilage Regeneration via Improving Chondrogenic Phenotype" *ACS Applied Materials & Interfaces* (2022): 14, 40674.

86. Feng, Q. Li, Q. Wen, H., et al., "Injection and Self‐Assembly of Bioinspired Stem Cell‐Laden Gelatin/Hyaluronic Acid Hybrid Microgels Promote Cartilage Repair In Vivo" *Advanced Functional Materials* (2019): 29, 1906690.

87. Amirsadeghi, A. Mahdavi, S. Jager, P. Kamperman, M. and J. Es Sayed, "3D-Printable Granular Hydrogel Composed of Hyaluronic Acid-Chitosan Hybrid Polyelectrolyte Complex Microgels" *Biomacromolecules* (2025).

88. Kong, J. Yao, Z. C. Stelzel, J. L., et al., "Granular Nanofiber-Hydrogel Composite-Programmed Regenerative Inflammation and Adipose Tissue Formation" *Advanced Healthcare Materials* (2025): 14, e2403094.

89. Zhang, J. Zeng, Y. Heng, Y., et al., "Enzyme‐Assisted Activation Technique for Producing Versatile Hydrogel Microparticle Scaffolds with High Surface Chemical Reactivity" *Advanced Functional Materials* (2024): 34, 202400858.

90. Zhang, J. Sun, X. Heng, Y., et al., "Transforming Cell-Drug Interaction through Granular Hydrogel-Mediated Delivery of Polyplex Nanoparticles for Enhanced Safety and Extended Efficacy in Gene Therapy" *ACS Applied Materials & Interfaces* (2024): 16, 39784.

91. Zhang, J. Wang, Y. Liu, Y., et al., "Reinforced Granular Hydrogels Scaffolds with Tunable Physicochemical Properties for Advanced Skin Tissue Engineering" *Advanced Science (Weinh)* (2025), e2415634.

92. Shaik, R. Brown, J. Xu, J., et al., "Cardiac Matrix-Derived Granular Hydrogel Enhances Cell Function in 3D Culture" *ACS Applied Materials & Interfaces* (2024): 16, 58346.

93. Li, Y. Song, W. Kong, L. He, Y. Li, H., "Injectable and Microporous Microgel-Fiber Granular Hydrogel Loaded with Bioglass and siRNA for Promoting Diabetic Wound Healing" *Small* (2024): 20, e2309599.

94. Kedzierski, A. Kheirabadi, S. Jaberi, A., et al., "Engineering the Hierarchical Porosity of Granular Hydrogel Scaffolds Using Porous Microgels to Improve Cell Recruitment and Tissue Integration" *Advanced Functional Materials* (2025): 35, 2417704.

95. D'Elia, A. Jones, O. Canziani, G., et al., "Injectable Granular Hydrogels Enable Avidity-Controlled Biotherapeutic Delivery" *ACS Biomaterials Science & Engineering* (2024): 10, 1577.

96. Highley, C. Song, K. Daly, A. and J. Burdick, "Jammed Microgel Inks for 3D Printing Applications" *Advanced Science (Weinh)* (2019): 6, 1801076.

97. Hsu, R. Chen, P. Fang, J., et al., "Adaptable Microporous Hydrogels of Propagating NGF-Gradient by Injectable Building Blocks for Accelerated Axonal Outgrowth" *Advanced Science (Weinh)* (2019): 6, 1900520.

98. Song, T. Zhao, F. Yan, L., et al., "Structure driven bio-responsive ability of injectable nanocomposite hydrogels for efficient bone regeneration" *Biomaterials* (2024): 309, 122601.

99. Mansson, L. de Wild, T. Peng, F., et al., "Preparation of colloidal molecules with temperature-tunable interactions from oppositely charged microgel spheres" *Soft Matter* (2019): 15, 8512.

100. Asadikorayem, M. Surman, F. Weber, P. Weber, D. and M. Zenobi-Wong,"Zwitterionic Granular Hydrogel for Cartilage Tissue Engineering" *Advanced Healthcare Materials* (2024): 13, e2301831.

101. Kang, Y. Liu, X. Wang, J., et al., "Rod‐Shaped Microgel Scaffolds with Interconnective Pores and Oxygen‐generating Functions Promote Skin Wound Healing and Alleviate Hypertrophic Scar Formation" *Advanced Functional Materials* (2025): 35, 2413678.

102. Liu, H. Huang, C. Chiang, M., et al., "Sustained Release of Nitric Oxide-Mediated Angiogenesis and Nerve Repair by Mussel-Inspired Adaptable Microreservoirs for Brain Traumatic Injury Therapy" *Advanced Healthcare Materials* (2024): 13, e2302315.

103. Ayala, A. Christ, G. and D. Griffin, "Cell-scale porosity in microporous annealed particle (MAP) scaffolds modulates immune response and promotes formation of innervated muscle fibers in volumetric muscle loss injuries" *bioRxiv* (2024), 2024.05.31.596879.

104. Coronel, M. Martin, K. Hunckler, M., et al., "Hydrolytically Degradable Microgels with Tunable Mechanical Properties Modulate the Host Immune Response" *Small* (2022): 18, e2106896.

105. Xin, S. Dai, J. Gregory, C. Han, A. and D. Alge, "Creating Physicochemical Gradients in Modular Microporous Annealed Particle Hydrogels via a Microfluidic Method" *Advanced Functional Materials* (2020): 30, 1907102.

106. Zamanian, B. Masaeli, M. Nichol, J. W., et al., "Interface-directed self-assembly of cell-laden microgels" *Small* (2010): 6, 937.

107. Asadikorayem, M. Brunel, L. Weber, P. Heilshorn, S. and M. Zenobi-Wong, "Porosity dominates over microgel stiffness for promoting chondrogenesis in zwitterionic granular hydrogels" *Biomaterials Science* (2024): 12, 5504.

108. Mahdieh, Z. Cherne, M. Fredrikson, J., et al., "Granular Matrigel: restructuring a trusted extracellular matrix material for improved permeability" *Biomedical Materials* (2022): 17, 045020.

109. Deo, K. Murali, A. Tronolone, J., et al., "Granular Biphasic Colloidal Hydrogels for 3D Bioprinting" *Advanced Healthcare Materials* (2024): 13, e2303810.

110. Di Caprio, N. Hughes, A. and J. Burdick, "Programmed shape transformations in cell-laden granular composites" *Science advances* (2025): 11, eadq5011.

111. Flegeau, K. Puiggali-Jou, A. and M. Zenobi-Wong, "Cartilage tissue engineering by extrusion bioprinting utilizing porous hyaluronic acid microgel bioinks" *Biofabrication* (2022): 14, 034105.

112. Kurt, E. and T. Segura, "Nucleic Acid Delivery from Granular Hydrogels" *Advanced Healthcare Materials* (2022): 11, e2101867.

113. Sheikhi, A. Lisa, D. Rutte, J., et al., "Microengineered Emulsion-to-Powder Technology for the High-Fidelity Preservation of Molecular, Colloidal, and Bulk Properties of Hydrogel Suspensions" *ACS Applied Polymer Materials* (2019): 1, 1935.

114. Sheikhi, A. de Rutte, J. Haghniaz, R., et al., "Microfluidic-enabled bottom-up hydrogels from annealable naturally-derived protein microbeads" *Biomaterials* (2019): 192, 560.

115. Kuang, G. Zhang, Q. Li, W. and Y. Zhao, "Biomimetic Tertiary Lymphoid Structures with Microporous Annealed Particle Scaffolds for Cancer Postoperative Therapy" *ACS Nano* (2024): 18, 9176.

116. Xu, F. Wu, C. Rengarajan, V., et al., "Three-dimensional magnetic assembly of microscale hydrogels" *Advanced Materials* (2011): 23, 4254.

117. Asadikorayerm, M. Weber, P. Surman, F. Puiggali-Jou, A. Zenobi-Wong, M., "Foreign Body Immune Response to Zwitterionic and Hyaluronic Acid Granular Hydrogels Made with Mechanical Fragmentation" *Advanced Healthcare Materials* (2025): 14, e2402890.

118. Anderson, A. Nicklow, E. and T. Segura, "Particle fraction is a bioactive cue in granular scaffolds" *Acta Biomaterialia* (2022): 150, 111.

119. Seymour, A. Kilian, D. Navarro, R. Hull, S. and S. Heilshorn, "3D printing microporous scaffolds from modular bioinks containing sacrificial, cell-encapsulating microgels" *Biomaterials Science* (2023): 11, 7598.

120. Liu, Y. Suarez-Arnedo, A. Riley, L., et al., "Spatial Confinement Modulates Macrophage Response in Microporous Annealed Particle (MAP) Scaffolds" *Advanced Healthcare Materials* (2023): 12, e2300823.

121. Xin, S. Wyman, O. and D. Alge, "Assembly of PEG Microgels into Porous Cell-Instructive 3D Scaffolds via Thiol-Ene Click Chemistry" *Advanced Healthcare Materials* (2018): 7, e1800160.

122. Wilson, K. Perez, S. Naffaa, M. Kelly, S. and T. Segura, "Stoichiometric Post-Modification of Hydrogel Microparticles Dictates Neural Stem Cell Fate in Microporous Annealed Particle Scaffolds" *Advanced Materials* (2022): 34, e2201921.

123. Mendes, B. Daly, A. Reis, R., et al., "Injectable hyaluronic acid and platelet lysate-derived granular hydrogels for biomedical applications" *Acta Biomaterialia* (2021): 119, 101.
